# Photoactivatable mRNA 5′ Cap Analogs for RNA‐Protein Crosslinking

**DOI:** 10.1002/advs.202400994

**Published:** 2024-07-24

**Authors:** Marcin Warminski, Katarzyna Grab, Kacper Szczepanski, Tomasz Spiewla, Joanna Zuberek, Joanna Kowalska, Jacek Jemielity

**Affiliations:** ^1^ Division of Biophysics Institute of Experimental Physics Faculty of Physics University of Warsaw Pasteura 5 Warsaw 02‐093 Poland; ^2^ Doctoral School of Exact and Natural Sciences University of Warsaw Zwirki i Wigury 93 Warsaw 02‐089 Poland; ^3^ Centre of New Technologies University of Warsaw Banacha 2c Warsaw 02‐097 Poland

**Keywords:** 5′ cap, cap‐binding proteins, mRNA, photoaffinity labeling, photocrosslinking

## Abstract

Chemical modification of messenger RNA (mRNA) has paved the way for advancing mRNA‐based therapeutics. The intricate process of mRNA translation in eukaryotes is orchestrated by numerous proteins involved in complex interaction networks. Many of them bind specifically to a unique structure at the mRNA 5′‐end, called 5′‐cap. Depending on the 5′‐terminal sequence and its methylation pattern, different proteins may be involved in the translation initiation and regulation, but a deeper understanding of these mechanisms requires specialized molecular tools to identify natural binders of mRNA 5′‐end variants. Here, a series of 8 new synthetic 5′‐cap analogs that allow the preparation of RNA molecules with photoreactive tags using a standard in vitro transcription reaction are reported. Two photoreactive tags and four different modification sites are selected to minimize potential interference with cap‐protein contacts and to provide complementary properties regarding crosslinking chemistry and molecular interactions. The tailored modification strategy allows for the generation of specific crosslinks with model cap‐binding proteins, such as eIF4E and Dcp2. The usefulness of the photoreactive cap analogs is also demonstrated for identifying the cap‐binding subunit in a multi‐protein complex, which makes them perfect candidates for further development of photoaffinity labeling probes to study more complex mRNA‐related processes.

## Graphical abstract

A deeper understanding of mRNA translation mechanisms requires new molecular tools to identify binders of different mRNA 5′‐end variants. A series of trinucleotide 5′‐cap analogs that allow for the straightforward preparation of RNA molecules with photoreactive tags capable of forming specific crosslinks with model cap‐binding proteins are reported. Such RNAs have also proved useful for identifying the cap‐binding subunit in multi‐protein complexes.

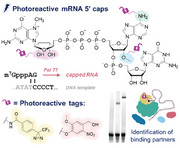

## 1 Introduction

**Figure 1:**
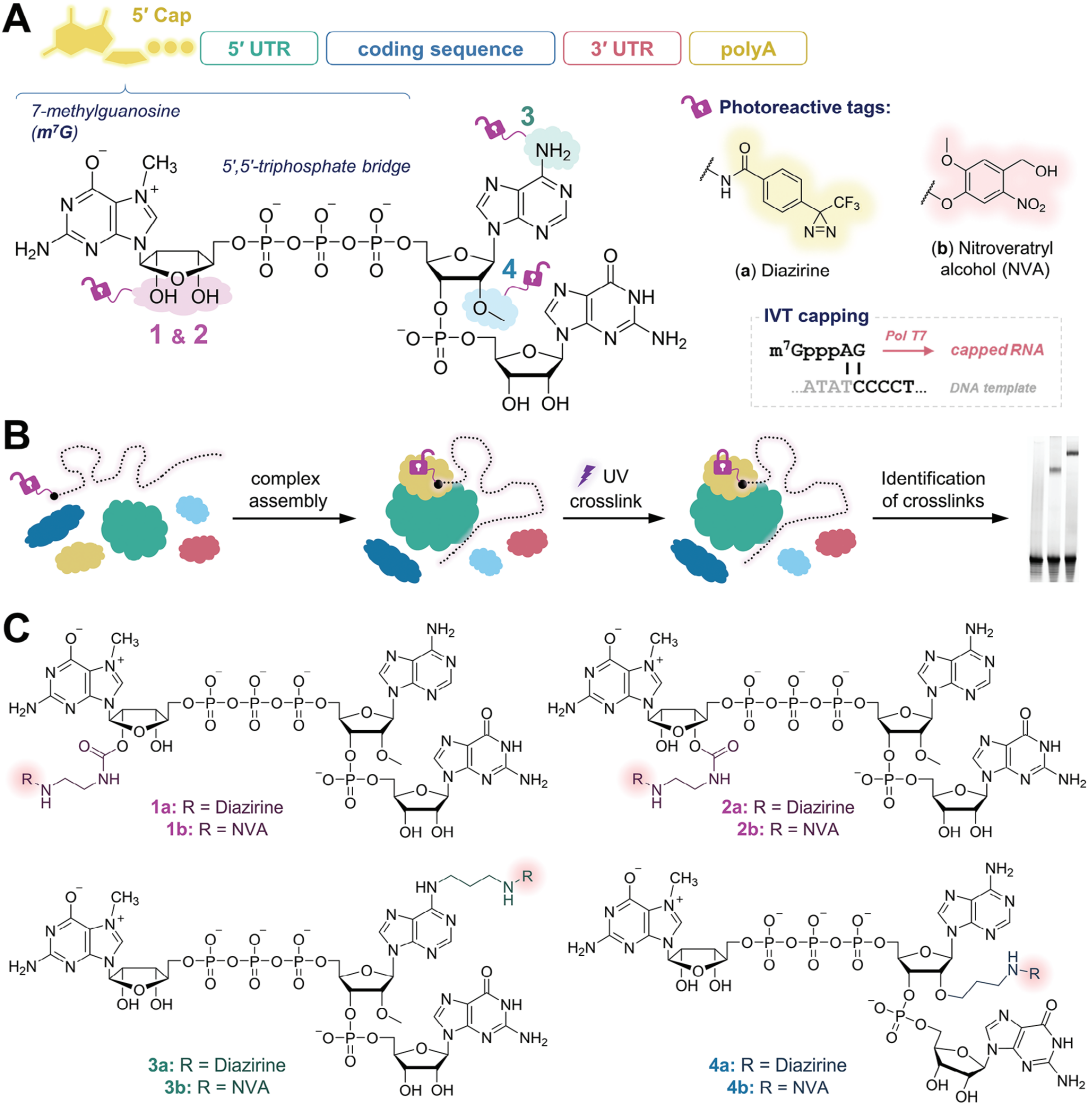
Design of the photoreactive trinucleotide 5′ cap analogs. A) Chemical structure of the trinucleotide 5′ cap analog (m^7^GpppA_m_pG) and its functionalization sites studied in this work (marked with colors and numbers corresponding to the compound designations). B) Schematic diagram of the use of photoreactive mRNAs to study cap‐protein interaction networks. C) Chemical structure of photoreactive trinucleotide cap analogs studied in this work.

Messenger RNA (mRNA) plays a central role in gene expression and is targeted by numerous proteins regulating protein biosynthesis. It has also emerged as a highly attractive and flexible platform for novel therapeutic strategies, including preventive vaccination (viral diseases), immunotherapeutics (cancer), or protein replacement therapies,^[^ ^1^ ^]^ which have already proven their effectiveness during the COVID‐19 pandemic. The key structural feature that distinguishes eukaryotic mRNA molecules from other types of RNA and determines their biological function is a unique structure called 5′ cap (**Figure** **1**A). The 5′ cap protects mRNA from premature degradation and controls its cellular fate by interacting with several proteins, including CBC (regulation of nuclear export), eIF4E (translation initiation), Dcp2 (cap removal leading to mRNA degradation), and some other more specialized proteins. Many of the cap‐binding proteins (CBPs) are actually components of large macromolecular complexes (e.g., eIF4E is a part of eIF4F, eIF3d is a part of eIF3, and Dcp2 is accompanied by several enhancers such as Dcp1, EDC3, PNRC2, etc.), which adds another layer of mRNA regulation.^[^ ^2^ ^]^ As a result, mRNA translation may be differentially regulated depending on the 5′ end sequence and methylation pattern. Besides the canonical translation initiation mediated by the cap‐eIF4E interactions, several alternative mechanisms have been identified for specific pools of mRNAs. These include both cap‐independent mechanisms (e.g., internal ribosome entry sites – IRES),^[^ ^3^ ^]^ and some cap‐dependent mechanisms involving eIF3 (mRNAs with specific 5′ UTRs)^[^ ^4^ ^]^ or Gemin5 (mRNA with 5′‐terminal oligopyrimidine tracts, 5′ TOP).^[^ ^5^ ^]^ It is also debated whether the natural modification of cap‐adjacent adenosine (m^6^A_m_) has a specific reader that determines its unclear biological function.^[^ ^6^, ^7^ ^]^ Further progress in this field appears to be hampered by the limited access to molecular tools suitable for studying mRNA interactions in their natural environment.

To address this problem, we turned our attention to photoaffinity probes, which have proven to be extremely useful in similar research areas. Through the conjugation of bioactive molecules with photoreactive tags and subsequent generation of highly reactive species, they enable the formation of covalent crosslinks with adjacent molecules, thereby facilitating the identification of interacting partners, particularly proteins. The process is conveniently triggered by light, offering gentle conditions that enable straightforward spatial and temporal control. Light‐inducible processes have also been successfully employed for photocaging nucleotides^[^ ^8^ ^]^ and controlling the translational activity of mRNAs, in either one‐way turn‐on mode^[^ ^9^ ^]^ or as reversible photoswitches.^[^ ^10^ ^]^ UV‐C light (254 nm) has been used for decades to form covalent crosslinks between natural nucleic acids and the proteins that bind them,^[^ ^11^ ^]^ and has been implemented as a critical step in a so‐called CLIP (crosslinking and immunoprecipitation) protocol,^[^ ^12^ ^]^ useful for the identification of natural binding partners in biological systems. However, such short‐wavelength UV light damages nucleic acids, therefore the more advanced CLIP techniques (e.g., PAR‐CLIP) use modified nucleobases, such as 5‐iodouridine, 4‐thiouracil, or 6‐thioguanosine, which absorb at longer wavelengths (>310 nm).^[^ ^13^ ^]^ Usually, the modifications are randomly distributed throughout the RNA chain, but 6‐thioguanosine has been introduced site‐selectively into the 5′‐end of mRNA by enzymatic incorporation of the modified dinucleotide m_2_^7,2′‐O^Gppp^6S^G cap analog and successfully applied to map the histone H4 cap‐binding pocket.^[^ ^14^ ^]^ Unfortunately, this strategy does not allow modification of adenosine, which is a common transcription start site nucleotide and its methylation is likely to be an important regulatory mechanism. In a more general approach, several external photoactivated tags, namely aryl azide, benzophenone, and diazirine, have been incorporated into short capped RNAs (at *N*7 positon of cap) to prepare the photoaffinity labeling (PAL) probes.^[^ ^15^ ^]^ However, each of these modifications significantly impaired binding to eIF4E, which made them translationally inactive. Here, we have rationally designed and synthesized a series of functionalized mRNA 5′ cap analogs and evaluated them as molecular tools that allow the preparation of photoreactive RNA molecules using standard in vitro transcription reaction. The resulting RNAs formed covalent bonds with model cap‐binding proteins and their complexes in a cap‐specific manner, demonstrating their usefulness for studying the 5′ cap interaction network (Figure 1B).

## 2 Results and Discussion

### 2.1 Design and Synthesis of Photoreactive Cap Analogs

To simplify the preparation of photoreactive mRNAs, we focused on small molecule 5′ cap analogs that can be incorporated into RNA of any sequence using a standard in vitro transcription (IVT) protocol. This requirement is perfectly met by the m^7^GpppA_m_pG structure (Figure 1A), which was previously identified as the most efficient IVT primer for T7 RNA polymerase^[^ ^7^ ^]^ and contains the 2′‐*O*‐methylation of adenosine – an important discriminator of *self*‐RNA.^[^ ^16^ ^]^ Another key aspect was selecting the appropriate chemistry and positions for attaching the photoreactive tags. On the one hand, the label should not interfere with the natural interactions between the cap and the CPBs,^[^ ^17^ ^]^ and on the other hand, it must be placed close to the interaction site to allow cap‐specific covalent crosslinking. For this reason, we limited the possible modification sites to the first two nucleotides, i.e., m^7^G and A, and selected three different positions of the cap structure (Figure 1A), which could provide complementary properties in terms of intermolecular interactions. For the conjugation of cap analogs with photoreactive tags, we decided to use the *N*‐hydroxysuccinimide (NHS) activation strategy, since it uses easily accessible reagents and produces a very compact amide moiety. Accordingly, all the trinucleotide cap analogs were functionalized with a primary amine group linked via a short alkyl spacer (Figure 1C) to ensure some flexibility of the linkage and the carboxyl derivatives of the photoreactive tags were activated into NHS esters.

The first attachment site was the ribose of 7‐methylguanosine (Figure 1A, shown in purple), which we had previously studied in the context of mRNA labeling and demonstrated that such modified transcripts undergo cap‐specific translation and Dcp2/Nudt16‐catalyzed decapping.^[^ ^18^ ^]^ The synthetic route involved solid‐phase synthesis of the 5′‐phosphorylated dinucleotide pA_m_pG using the phosphoramidite approach,^[^ ^7^ ^]^ followed by activation of the 5′ phosphate and ZnCl_2_‐mediated coupling with a functionalized 7‐methylguanosine diphosphate derivative L2_N_‐m^7^GDP, prepared as described previously (Figure S1A, Supporting Information).^[^ ^19^ ^]^ This strategy afforded a mixture of 2′‐*O‐* and 3′‐*O*‐substituted cap analogs **1** and **2**, which was isolated by ion exchange chromatography in 58% yield. The regioisomers were separated by RP HPLC after conjugation with the photoreactive tags.

Our next candidate was the *N* ^6^ position of the adenosine nucleobase (Figure 1A, shown in green), which has also been shown to be well tolerated by several CBPs, including eIF4E, DcpS, and CBC.^[^ ^20^ ^]^ It could be considered as an extension of the natural m^6^A_m_ cap, which appears to have a beneficial or at least neutral effect on mRNA translation and does not interfere with decapping by Dcp2 in vitro.^[^ ^6^, ^7^, ^21^ ^]^ The corresponding compound **3** was obtained in a ZnCl_2_‐mediated coupling reaction between *P*‐imidazolide of 7‐methylguanosine 5′‐diphosphate (m^7^GDP‐Im) and the dinucleotide 5′‐phosphate pA_m_^L3N^pG and isolated in 72% yield (Figure S1B, Supporting Information). The dinucleotide was prepared by solid‐phase synthesis using *N*6‐aminopropyladenosine phosphoramidite accessible by direct alkylation of A_m_ phosphoramidite under phase‐transfer conditions.^[^ ^22^ ^]^

Another natural methylation site within the 5′ cap is a 2′‐*O* position of the first transcribed nucleotide (adenosine in this case) forming the cap‐1 structure (Figure 1A, shown in blue). Its biological function is based on the disruption of mRNA binding by several innate immune receptors, such as IFIT1 and RIG‐I,^[^ ^16^ ^]^ but it seems to have negligible effect on the interactions with eIF4E and Dcp2.^[^ ^7^ ^]^ To the best of our knowledge, extensions of these methyl substituents have not been tested before, except for the mention of enzymatic hexynylation of short capped RNA by the CMTr1 enzyme.^[^ ^23^ ^]^ The synthetic pathway for the 2′‐*O*‐aminopropyladenosine cap **4** was analogous to the synthesis of compound **3** (46% yield of the coupling reaction; Figure S1C, Supporting Information) and the corresponding phosphoramidite was synthesized as described in the literature.^[^ ^24^ ^]^

**Figure 2:**
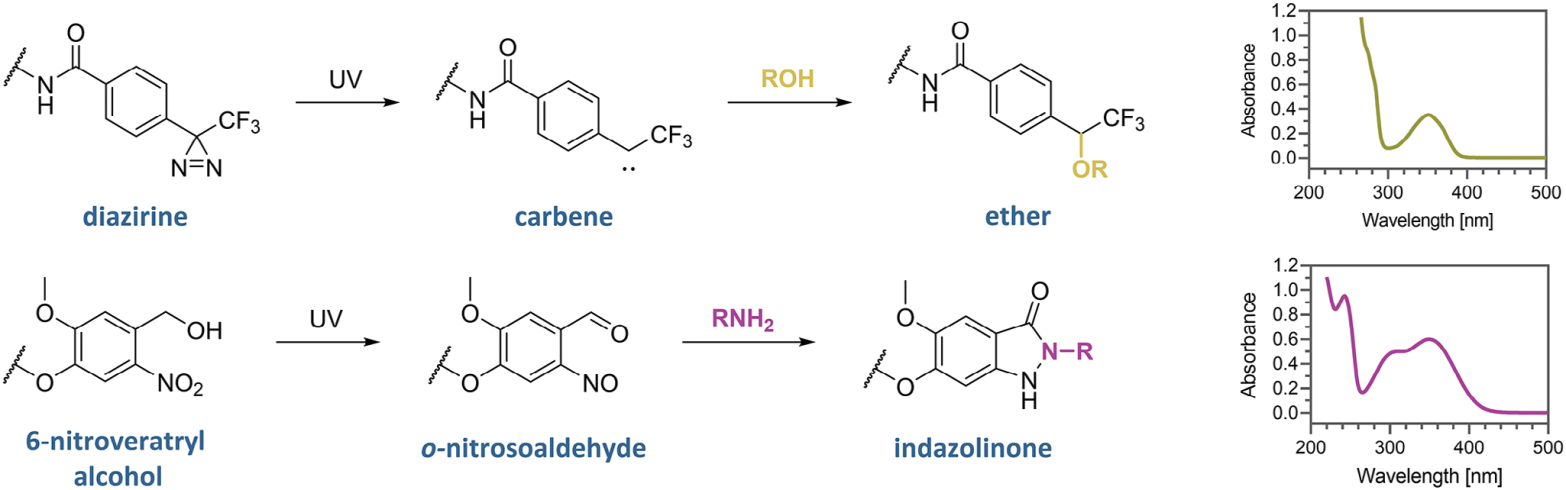
Photoactivation reactions and UV–vis spectra of diazirine and 6 nitroveratryl alcohol.

The choice of photoreactive groups was mainly dictated by their photochemical properties, especially the excitation wavelength, since the high‐energy UV‐B and UV‐C radiation causes damage to RNA molecules. One of the most commonly used photoactivatable compounds in this context are diazirines, which upon excitation at 350–370 nm generates unstable carbenes, reactive toward nucleophilic centers, mainly ─OH groups (present in Ser, Thr, and Tyr sidechains; **Figure** **2**). We decided to use the commercially available 3‐(4‐carboxyphenyl)‐3‐trifluoromethyl diazirine, which offers a good balance between reactivity and specificity.^[^ ^25^ ^]^ The compound was converted to the NHS ester and conjugated with trinucleotides **1–4** to give a series of photoreactive cap analogs **1a–4a** (Figure 1C; Figure S2, Supporting Information) in 50–62% yields.

Since the carbene moiety reacts readily with water molecules, this strategy may not be very efficient for proteins with solvent‐exposed binding sites typical of nucleic acid‐binding proteins. To address this potential problem, we also tested another class of photoreactive molecules – the 6‐nitroveratryl alcohol (NVA) derivatives.^[^ ^26^ ^]^ They have attracted the most interest as photocleavable groups, but the product of the UV‐induced reaction is a 2‐nitrosobenzaldehyde that reacts quite selectively with primary amines (present in Lys) to form stable indazolinones in a Davis‐Beirut reaction (Figure 2; Figure S3C, Supporting Information).^[^ ^27^ ^]^ Based on the published protocol,^[^ ^28^ ^]^ we synthesized the derivative of 6‐nitroveratryl alcohol bearing a carboxyl‐terminated linker and conjugated it with trinucleotides **1–4** to afford a series of photoreactive cap analogs **1b–4b** (Figure 1C; Figure S2, Supporting Information) in 45–60% yields.

**Figure 3:**
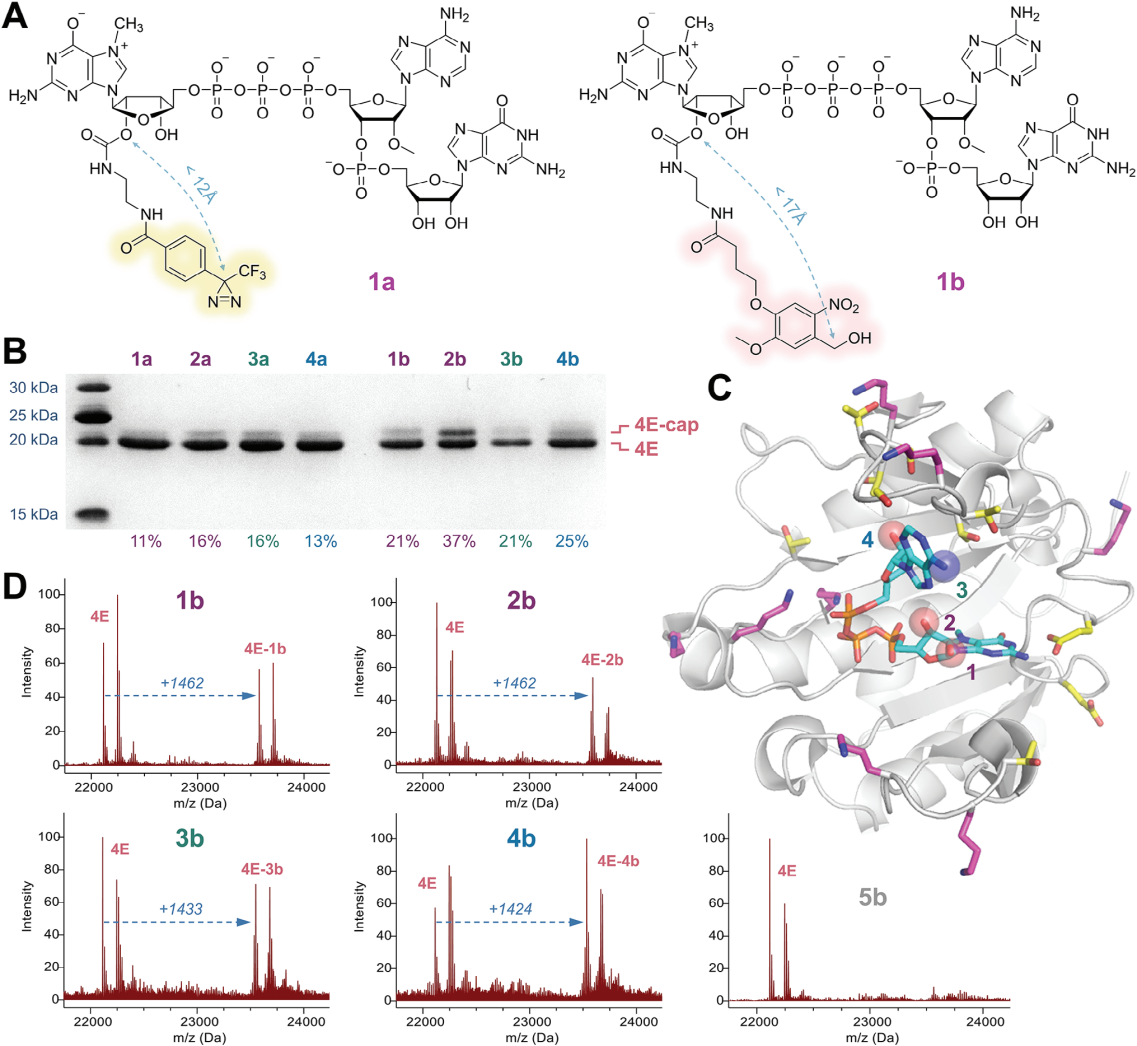
Optimization of photocrosslinking reaction between trinucleotide cap analogs **1a/b–4a/b** and model protein eIF4E. A) Estimation of the distance between photoreactive center and label attachment site in compounds **1a** and **1b**. B) SDS PAGE analysis of photocrosslinking reaction mixtures; C) X‐ray crystal structure of the m^7^GpppA/eIF4E complex (pdb: 1WKW). Cap functionalization sites were denoted with semitransparent spheres. The sidechains of lysine residues (magenta) and serine/threonine/glutamic acid (yellow) within the range accessible by the tag were displayed as sticks. D) Deconvoluted mass spectra of the reaction mixtures. The two peaks of eIF4E protein (M and M+131) result from incomplete processing of the N‐terminal Met by *E. coli* Methionine Aminopeptidase^[^ ^30^ ^]^ and are also present in the spectrum of non‐irradiated protein.

Simple molecular modeling showed that the reactive center generated from diazirine can reach no further than 11–12 Å from the cap attachment site, while the aldehyde group generated from NVA can reach no further than 16–17 Å (**Figure** **3**A). For both tags, the accessible range is significantly smaller than the diameter of ≈20 kDa globular proteins, such as eIF4E (average diameter of ≈40Å), which should provide both sufficient selectivity and high probability of reaching an appropriate functional group on the surface of proteins.

### 2.2 Optimization of UV‐Initiated Activation

Next, we characterized the spectroscopic and photochemical properties of our trinucleotide cap analogs **1a–4a** and **1b–4b** to find the optimal conditions for UV‐induced activation of the tags. To this end, we recorded UV–vis spectra of all compounds dissolved in phosphate buffer pH 7 (see Compounds characterization in ESI). Except for the strong absorption band at ≈260 nm, characteristic for m^7^GpppA_m_pG nucleobases, we observed a weaker band at longer wavelengths, reminiscent of tag excitation. In the case of diazirine‐labeled compounds **1a–4a**, the band was centered ≈350 nm and its intensity was ≈80‐fold lower than the maximum of the band corresponding to nucleobases. The UV spectra of analogs **1b–4b** showed a more intense band with a maximum ≈360 nm, which was only 4 times lower than that of the nucleotide chromophore.

Then, we exposed each analog to the UV light (6 W tube lamp, 365 nm) and recorded the UV–vis spectra of the samples after 30 and 60 min of irradiation. We observed the disappearance of the bands at 350–360 nm within 30 min without any significant changes upon further irradiation. The RP HPLC analysis of the irradiated samples confirmed the complete conversion of the starting material. Each of the diazirine‐labeled cap analogs **1a–4a** produced only one compound, which was identified by MS as the product of the reaction between carbene and water molecule (Figure S3A, Supporting Information). In the case of the NVA‐labeled analogs **1b–4b**, we observed a mixture of products with the *o*‐nitrosoaldehyde as the major one (Figure S3B, Supporting Information).

A very low intensity of the diazirine excitation band for analogs **1a–4a** hindered the analysis of the photoreaction kinetics, but we were able to estimate the half‐life of the free diazirine carboxylic acid upon UV irradiation to be ca. 15 min. The compounds were found to be relatively stable in daylight or laboratory light, with a half‐life value of ≈40 h (≈2% decomposition per hour). The similar analysis of nitroveratryl carboxylate at different wavelengths showed a more complex changes of the spectrum, reflecting the reaction mechanism. Only the first, relatively fast step – the tautomerization of *o*‐nitrobenzyl alcohol into the *aci*‐nitro form – requires UV photons, while the subsequent reactions can proceed in the dark (Figure S4B, Supporting Information).^[^ ^29^ ^]^ To estimate the apparent rate of these reactions, we irradiated the solution of compound **4b** in phosphate buffer pH 7.0 for 3 min with UV LEDs (369 nm, 2 × 3 W) and then monitored the changes in UV–vis absorption at several wavelengths corresponding to the largest changes (Figure S4A, Supporting Information). A one‐phase exponential model with a half‐life of 10.9 min provided a very good fit to the experimental data (R^2^ > 0.998 for all curves). An analogous experiment performed in HEPES buffer pH 8.0 yielded a half‐life value of 6.9 min, while in TRIS pH 8.0 the half‐life was 5.1 min (Figure S4A, Supporting Information). After reaching a plateau, all samples were irradiated for further 2 min, but no further changes in the UV–vis spectra were observed, indicating that the reaction was complete. The RP HPLC analysis of the TRIS‐buffered solution confirmed that all of the cap was converted into **4b**‐TRIS indazolinone and indazole conjugates (Figure S3C, Supporting Information).

### 2.3 UV‐Crosslinking to Model Protein

Having determined the minimum irradiation time required to activate each class of cap analogs, we moved on to the actual photocrosslinking experiments. We started with the model protein – a recombinant mouse eukaryotic translation initiation factor 4E (eIF4E), which is 99% identical to its human ortholog. The protein was mixed with a 10‐fold excess of the trinucleotide cap analogs and irradiated at 365 nm for 30 min. The SDS‐PAGE analysis of the resulting mixture clearly showed the formation of a cap‐protein conjugate that migrated slightly slower than the parent protein (Figure 3B). The densitometric analysis of the stained gel yielded estimated crosslinking efficiencies of 11–16% for diazirine‐labeled analogs and 21–37% for NVA derivatives. The addition of a fresh portion of the photoreactive cap with another round of irradiation did not result in a significant improvement in the crosslinking yield (Figure S6, Supporting Information).

The lower photocrosslinking yield observed for analogs **1a–4a** is probably due to the competing reaction of carbene and water molecules, as the cap‐binding site of the eIF4E protein is highly exposed to solvent (Figure 3C). The *o*‐nitrosoaldehydes generated from analogs **1b–4b** are stable in aqueous solutions and have more time to encounter the lysine sidechain on the protein surface. On the other hand, the chemical stability of the aldehydes might lead to non‐specific crosslinking that does not require the formation of the protein‐cap complex. To verify this, we synthesized compound **5b** (NVA‐L2_N_‐GpppA_m_pG) – the analog of compound **1b**, which lacks the *N*7 methyl group and thus should not bind to eIF4E effectively. MS analysis of a mixture of compound **5b** and eIF4E protein in HEPES buffer irradiated at 365 nm showed a significant amount of the covalent conjugate, but we were able to prevent its formation by performing the reaction in TRIS buffer (Figure S5A, Supporting Information). The TRIS molecule contains a primary amine group –NH_2_ acting as a scavenger for photoactivated NVA derivatives that are not bound to the protein (Figure S3C, Supporting Information). Under the same conditions, compounds **1b–4b** crosslinked to eIF4E in ca. 35–50% (Figure 3D).

Finally, to find the optimal pH value for photocrosslinking reactions, the analogs **1a/b–4a/b** were irradiated in the presence of eIF4E (tenfold excess of the cap) in different buffers and the resulting mixtures were analyzed by SDS‐PAGE. For the NVA‐labeled analogs **1b–4b**, the photocrosslinking efficiency showed a clear S‐shaped dependence on pH (higher efficiency at higher pH), with the inflection point between pH 7 and 8, whereas for the diazirine‐labeled analogs **1a–4a**, no significant differences between samples were observed (Figure S5B, Supporting Information). Importantly, we did not observe any crosslinks in the samples containing 1% SDS as a protein denaturing agent. We further confirmed the cap‐specific nature of the crosslinking reaction by irradiating the mixture of photoreactive analogs and eIF4E protein in the presence of a photostable cap (m^7^GpppA_m_pG) as a competitor (Figure S6, Supporting Information). As expected, for both diazirine and nitroveratryl analogs, we observed a decrease in crosslinking yield with increasing concentration of the m^7^GpppA_m_pG (0, 0.5, 1, 2, 10‐fold excess over photoreactive cap).

### 2.4 Enzymatic Synthesis of Capped RNAs

Next, we tested whether compounds **1a/b–4a/b** are efficient in vitro transcription primers suitable for the preparation of RNAs capped with photoactivatable analogs. The T7 RNA polymerase‐catalyzed IVT reactions were performed from the DNA template containing the φ6.5 promoter followed by a sequence of 35 nucleotides using a threefold excess of the trinucleotide over GTP. The products were purified by RP HPLC and their quality was verified by PAGE (Figure S7A, Supporting Information). The observed heterogeneity of the RNAs (the bands above and below the main band) results from abortive initiation and termination of the transcription reaction,^[^ ^31^ ^]^ but should not affect the photocrosslinking yield. Most importantly, all the cap analogs modified within the ribose of 7‐methylguanosine (**1a/b**, **2a/b**) were efficiently incorporated into RNA (80–90% capping according to RP HPLC analysis versus ≈90% typical for unmodified m^7^GpppA_m_pG), whereas *N*6 modified analogs **3a/b** and the 2′‐*O*‐alkylated analogs **4a/b** were slightly less efficient IVT primers (43–86% capping). In addition, the latter ones produced mixtures of correctly and reverse‐capped RNAs, which were separated by RP HPLC and subjected to decapping with Dcp2 to determine cap orientation (Figure S7B, Supporting Information). The correctly capped transcripts were decapped quite efficiently to RNAs that were 1 nucleotide shorter (m^7^GDP cleaved off), whereas the incorrectly capped transcripts were decapped very slowly to produce shorter RNAs (pApG cleaved off; Figure S7C, Supporting Information).

Two cap analogs modified within the ribose of 7‐methylguanosine (**1b** and **2a**), which were the most efficient IVT primers, were also incorporated into full‐length mRNA encoding Firefly luciferase and their translational activity was tested in A549 and JAWS II cell lines (Figure S8, Supporting Information).

Both modified mRNAs produced reporter protein at levels comparable to m^7^GpppA_m_pG‐capped mRNA, suggesting that the photoreactive tags, as intended, do not significantly interfere with cap‐dependent translation initiation. The diazirine‐labeled mRNA was even translated at a slightly higher rate, especially in A549 cells, but the effect was not statistically significant in JAWS II. Interestingly, UV irradiation of A549 cell cultures 30 min after mRNA transfection resulted in a decrease in protein levels produced from both modified mRNAs, while for the natural mRNA it was unaffected. The decrease seems to be more pronounced in the case of the NVA‐labeled mRNA, possibly due to its crosslinking with the transfection reagent (as it contains a primary amine group), which was not fully released, although in general UV exposure increased the variability of the data.

### 2.5 UV‐Crosslinking of Capped RNAs

With the full set of photoreactive short capped RNAs in hand, we performed crosslinking experiments with the two major cap‐binding proteins: eIF4E (translation factor) and Dcp2 (decapping enzyme). The SDS‐PAGE analysis followed by protein staining revealed some additional slower‐migrating but highly smeared bands (data not shown), most likely corresponding to protein‐RNA conjugates, but it was difficult to estimate the yield of the reactions. Therefore, we switched to TBE‐PAGE analysis and visualized RNAs instead, which provided additional information about the stability of the transcripts under UV irradiation.

Again, we started with **1a**‐RNA_35_ and eIF4E as a model cap‐binding protein to optimize the cross‐linking conditions. A solution of RNA and eIF4E was irradiated with UV light (369 nm, 3 W LED), and the samples were collected for PAGE analysis at different time points (Figure S9, Supporting Information). After 5 min ≈15% of the RNA was crosslinked to the protein, and the conversion reached a plateau at ≈20% after 15 min of irradiation. Importantly, we did not observe any significant degradation of RNA upon UV irradiation, even after 2 h.

**Figure 4:**
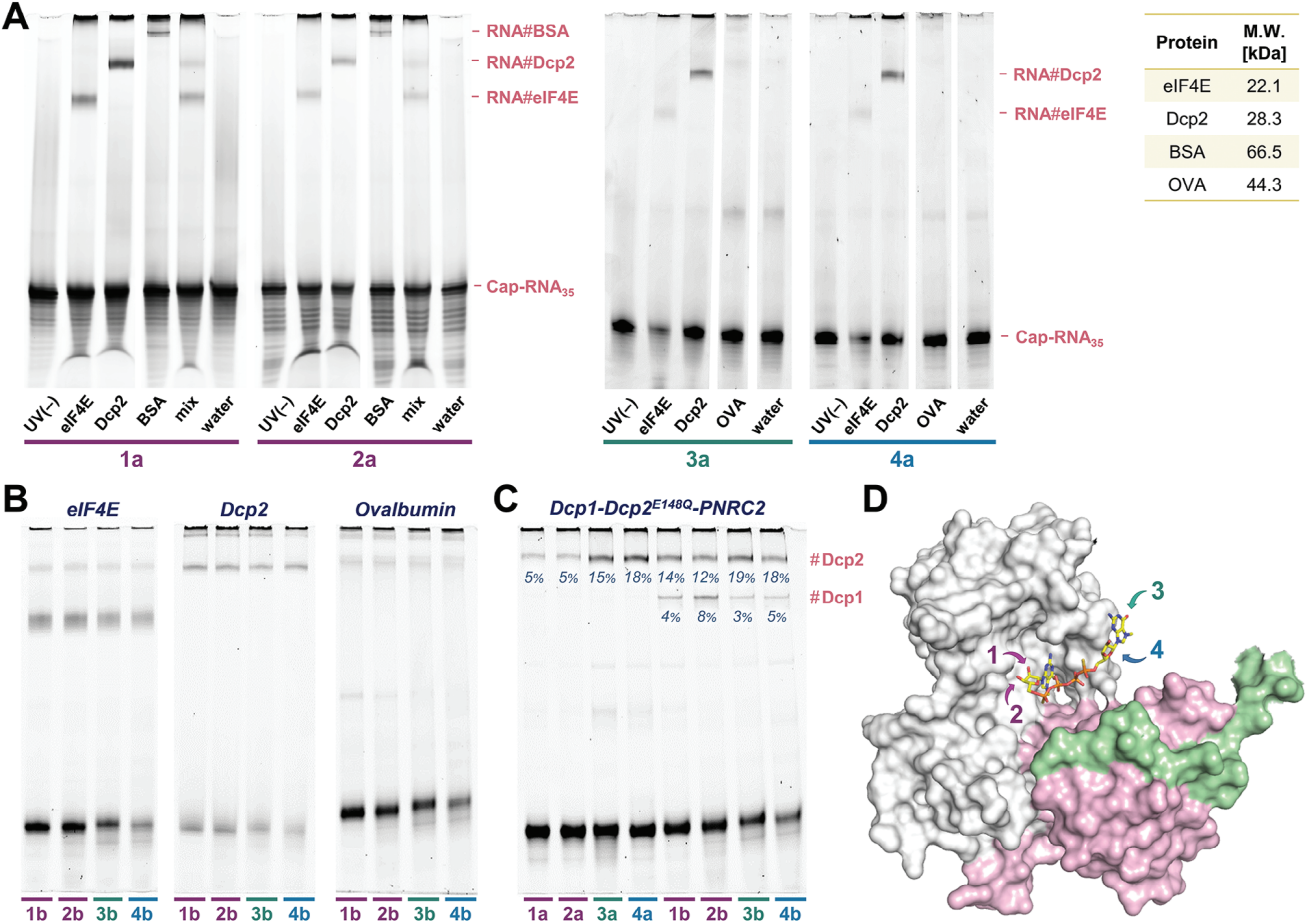
TBE‐PAGE analysis of photocrosslinking reactions between model proteins and RNAs capped with photoreactive analogs. A) Analysis of RNAs capped with diazirine‐labeled analogs (**1a–4a**); B) Analysis of RNAs capped with NVA‐labeled analogs (**1b–4b**); C) Analysis of cap interactions with multiprotein complex Dcp1‐Dcp2‐PNRC2 using RNAs capped with photoreactive analogs; D) X‐Ray crystal structure of Dcp1‐Dcp2‐PNRC2 complex with dinucleotide inhibitor (pdb: 5kq4).

We then used the optimized conditions to crosslink our diazirine‐labeled RNAs with several cap‐binding proteins and tested the specificity of the reaction. TBE‐PAGE analysis of the mixtures irradiated for 15 min showed clear bands corresponding to RNA‐eIF4E and RNA‐Dcp2 crosslinks (**Figure** **4**A). We also observed crosslinking to BSA protein (bovine serum albumin), probably due to its oligonucleotide‐binding properties,^[^ ^32^ ^]^ but it was not visible in the sample with a mixture of eIF4E, Dcp2, and BSA proteins at equal concentrations. In further experiments, we switched to ovalbumin (OVA) as a negative control and confirmed that it does not crosslink to diazirine‐labeled RNAs. eIF4E crosslinked to RNAs capped with analogs **1a** and **2a** (modified within the m^7^G ribose) with ≈20% efficiency and with RNAs capped with analogs **3a** and **4a** (labeled at the adenosine) with less than 10% efficiency, while Dcp2 generally crosslinked more efficiently (15–20%), with a slight preference for the **2a**‐RNA (30%). This observation reflects the different shapes of the cap‐binding pockets of the two proteins, resulting in different contact areas and solvent exposures of the label in their complexes. For NVA‐labeled RNAs, we observed a generally higher crosslinking efficiency with a slight preference for the Dcp2 enzyme, but the reactions were less specific than for diazirine derivatives, as suggested by faint bands corresponding to crosslinks with ovalbumin (Figure 4B).

Finally, we wanted to test whether our photoreactive RNAs could be used to identify the cap‐binding subunits in multiprotein complexes. To this end, we irradiated each RNA in the presence of a ternary Dcp1‐Dcp2‐PNRC2 complex (catalytically inactive mutant) and analyzed the mixtures by TBE‐PAGE (Figure 4C). The diazirine derivatives crosslinked exclusively to the Dcp2 portion, whereas for NVA‐RNAs we observed two bands corresponding to RNA‐Dcp2 and RNA‐Dcp1. Interestingly, the relative intensity of these bands differed depending on the labeling site, with the highest #Dcp1/#Dcp2 ratio for **2b**‐RNA and the lowest for **3b**‐RNA. This result is consistent with the X‐ray structure of the decapping complex bound to the dinucleotide inhibitor (Figure 4D). The cap structure is mainly bound to Dcp2 (white in Figure 4D), making it a preferred target for short‐lived tags, but some of the residues point to Dcp1 (pink in Figure 4D) and longer‐lived species could also modify this subunit.

### 2.6 Photoaffinity Labeling (PAL) Probes

**Figure 5:**
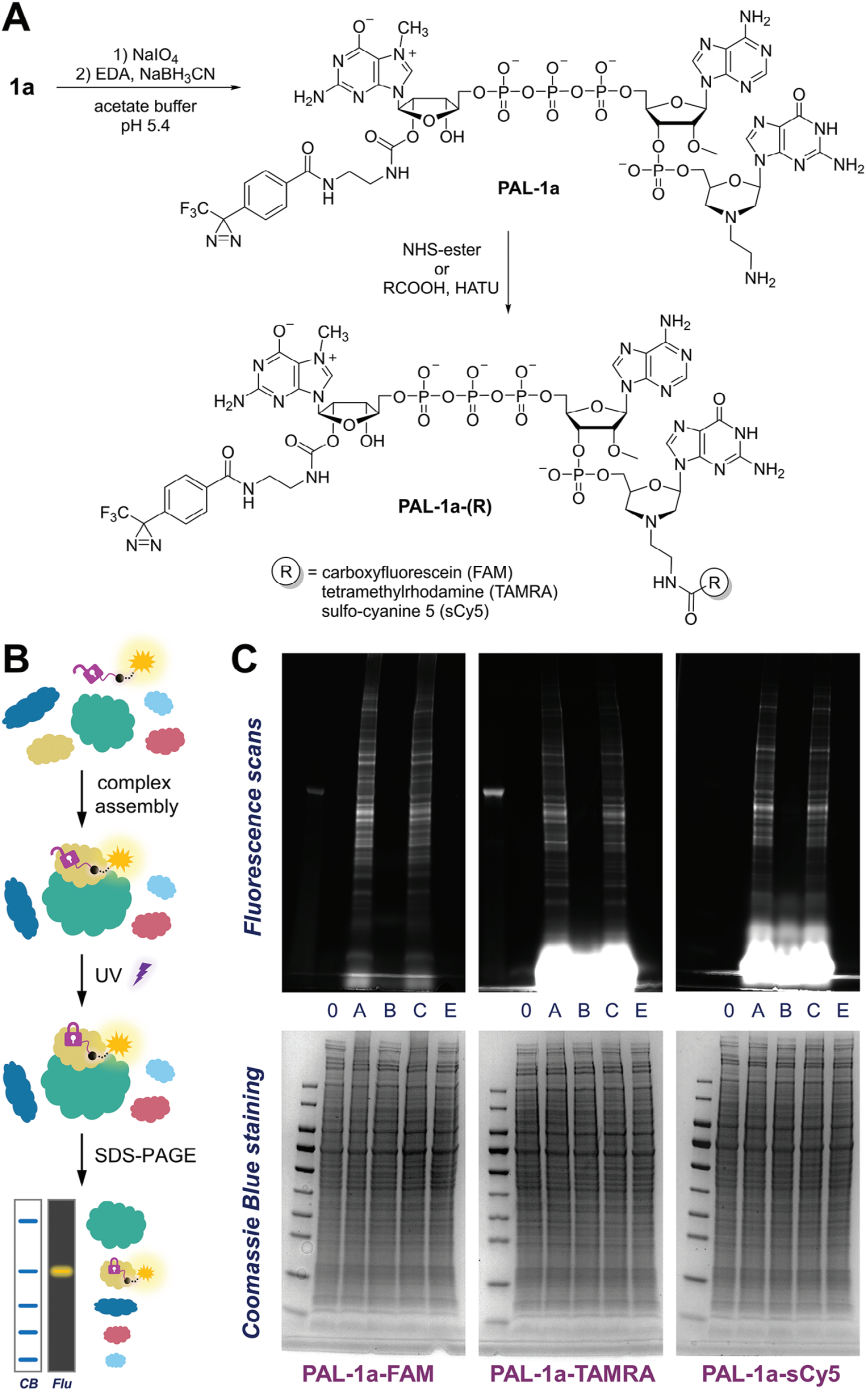
Evaluation of photoaffinity labeling (PAL) probes in HEK293F extracts. A) Chemical synthesis of fluorescent PAL probes used in this study; B) schematic idea of the experiment; C) SDS‐PAGE analysis of the resulting mixtures: *lanes 0*: non‐irradiated HEK293F cell extracts; *A*: a mixture of PAL probe and cell extract irradiated at 369 nm for 30 min; *B*: non‐irradiated mixture of PAL probe and cell extract; *C*: a mixture of PAL probe and cell extract preincubated with m^7^GpppA_m_pG, irradiated for 30 min; *E*: HEK293F cell extracts irradiated for 30 min.

To visualize cap‐binding proteins in a more complex system, such as cell extract (**Figure** **5**B), we converted one of our photoreactive cap analogs into photoaffinity labeling (PAL) probes. To this end, analog **1a** was subjected to periodate oxidation followed by reductive amination (Figure 5A),^[^ ^33^ ^]^ and the resulting 3′‐amino‐functionalized cap **PAL‐1a** was labeled with different fluorophores (carboxyfluorescein – FAM, tetramethylrhodamine – TAMRA, and sulfo‐cyanine 5 – sCy5). Each PAL probe was incubated with HEK293F cell extract and irradiated at 369 nm for 30 min. The mixtures were analyzed by SDS‐PAGE and the gels were imaged on a fluorescence scanner and then stained with Coomassie Blue (Figure 5C). As negative controls, we prepared samples without PAL probe (0 – non‐irradiated, E – irradiated) and the non‐irradiated mixture of cell extract and PAL probe (sample B). To verify the specificity of the crosslinks, we also included samples with a tenfold excess of m^7^GpppA_m_pG as a competitor (sample C). Fluorescence imaging showed several bands, but only in the samples containing PAL probes and irradiated with UV light, while Coomassie staining showed no differences between the samples. Notably, the intensity of the fluorescence signals was slightly decreased in the samples containing cell extract preincubated with a photostable competitor, and the pattern of fluorescent bands did not differ much between PAL probes with different fluorophores. All this suggests a cap‐specific nature of the observed crosslinks, which is critical for the application of our PAL probes in these types of studies.

## 3 Conclusion

In conclusion, we designed and synthesized 8 new photoreactive 5′ cap analogs suitable for the preparation of photoreactive mRNAs by in vitro transcription under standard conditions. Four different cap modification sites were selected to minimize potential interference with cap‐protein contacts and to provide complementary properties in terms of intermolecular interactions. All the analogs and short RNAs capped with them were evaluated positively in photocrosslinking reactions with model proteins, including translation initiation factor eIF4E and decapping enzyme Dcp2. The photocrosslinking yields reached up to 30%, which is much higher than for the previously reported *N*7‐guanosine derivatives,^[^ ^15^ ^]^ suggesting that the chosen modification sites are indeed compatible with model cap‐binding proteins. Our chemical approach to modifying the cap structure is also highly versatile in terms of synthetic scale, range of photoreactive tags attached, and compatibility with other cap modifications (both natural and synthetic). We have demonstrated the usefulness of our photoreactive RNAs for identifying the cap‐binding subunit in a multiprotein complex, making them an attractive starting point for the synthesis of photoaffinity labeling (PAL) probes to study more complex mRNA‐related processes. As a proof of concept, analog **1a** was modified at the 3′‐end with several fluorophores and irradiated in the presence of HEK293F cell extracts. SDS‐PAGE analysis of the resulting mixtures revealed several proteins that were covalently modified with a fluorophore in a cap‐specific manner. Despite the recent success of mRNA‐based antiviral vaccines, a better understanding of the cellular mechanisms of translation regulation is needed to extend the therapeutic potential of RNAs to other areas of medicine, such as cancer immunotherapy or protein replacement therapies. We believe that the cap‐based PAL probes would provide powerful tools for unraveling the intricate biological roles of different 5′‐end variants of natural transcripts, as well as for identifying the interactome of exogenous therapeutic mRNAs.

## 4 Experimental Section

### General Information

Solvents, chemical reagents, and starting materials were purchased from commercial sources. The *N*6‐phthalimidopropyladenosine and 2′‐*O*‐phthalimidopropyladenosine phosphoramidites were synthesized as described earlier.^[^ ^22^, ^24^ ^]^ The following modified mononucleotides were synthesized according to the previously published procedures: *N*7‐methylguanosine 5′‐*O*‐diphosphate *P*‐imidazolide (m^7^GDP‐Im),^[^ ^34^ ^]^ 2′‐*O*/3′‐*O*‐aminoethylcarbamoylguanosine 5′‐*O*‐diphosphate (L2_N_‐GDP) and 2′‐*O*/3′‐*O*‐aminoethylcarbamoyl‐*N*7‐methylguanosine 5′‐*O*‐diphosphate (L2_N_‐m^7^GDP).^[^ ^19^ ^]^

All the non‐labeled compounds were isolated from reaction mixtures by ion‐exchange chromatography on DEAE Sephadex A‐25 (HCO_3_^−^ form). The column was washed with water, loaded with the reaction mixture and then washed thoroughly with water until the eluate did not form a visible precipitate with 1% AgNO_3_ solution. Nucleotides were eluted using a linear gradient of triethylammonium bicarbonate (TEAB) in deionized water and the fractions were collected based on the absorption at 260 nm. After evaporation of the eluate under reduced pressure with repeated additions of 96% ethanol and then acetonitrile, the nucleotides were isolated as triethylammonium salts. The labeled trinucleotide cap analogs isolated using semi‐preparative RP‐HPLC with Gemini NX‐C18 (Phenomenex) RP HPLC column (150 × 10 mm, 5 µm, 110 Å, flow rate 5.0 mL min^−1^) with UV detection at 254 nm and isolated from the eluate by freeze–drying.

The synthesis yields were calculated based on the optical density (mOD = absorbance of the solution × volume in mL) of combined fractions measured in 0.1 m phosphate buffer pH 7 at 260 nm. The following extinction coefficient values were assumed in the calculations: ε = 27.1 L mmol^−1^ cm^−1^ for all dinucleotides containing A and G nucleobases (compounds **6** and **7**), ε = 32.0 L mmol^−1^ cm^−1^ for all trinucleotide cap analogs (**1–4**, **1a–4a**, **1b–4b**). The structure and homogeneity of each compound were confirmed by re‐chromatography by RP‐HPLC and high‐resolution mass spectrometry with electrospray ionization (HRMS‐ESI) in negative ions mode. Analytical HPLC was performed using a Gemini NX‐C18 HPLC column (4.6 × 150 mm, 3 µm, 110 Å, flow rate 1.0 mL min^−1^) with a 0–50% linear gradient of methanol in 0.05 m ammonium acetate buffer (pH 5.9) in 15 min (conditions A) or in 7.5 min (conditions B), or a 0–100% linear gradient of acetonitrile in 0.05 m ammonium acetate buffer (pH 5.9) in 15 min (conditions C), and UV detection at 254 nm. High‐resolution mass spectra were recorded with LTQ Orbitrap Velos (Thermo Scientific, high resolution). NMR spectra were recorded at 25 °C with a Bruker Avance III HD spectrometer at 500.24 MHz (^1^H NMR) using a 5 mm PABBO BB/19F‐1H/D Z‐GRD probe. The raw NMR data were processed using MestReNova v 12.0.2‐20910 Software. The UV–vis spectra were recorded using a Shimadzu UV‐1800 spectrophotometer (slit width: 1 nm, sampling interval: 1 nm) and plotted using Prism 8 (GraphPad Software Inc., San Diego, CA). UV‐crosslinking reactions were performed using either a filtered lamp with a UV tube (6 W, 365 nm, VL‐6.LC, Vilber) or power LEDs (2 × 3 W, 369 nm, EP‐U4545K‐A3‐369, Epileds). Cap‐eIF4E crosslinks were analyzed by RP HPLC (stationary phase: C18, mobile phase: water, acetonitrile, 0.1% formic acid) with ESI‐MS detection (Waters Synapt G2).

### Synthesis of Cap Analogs **1** and **2** (L2_N_‐m^7^GpppA_m_pG)

Step 1: Dinucleotide 5′‐phosphate pA_m_pG (synthesized as described earlier;^[^ ^7^ ^]^ 3840 mOD_260nm_, 0.142 mmol) was dissolved in DMF (2.8 mL) followed by addition of imidazole (154 mg, 2.27 mmol, 16 equivalents), 2,2′‐dithiodipiridine (187 mg, 0.85 mmol, 6 equivalents), triethylamine (59.4 µL, 0.42 mmol, 3 equivalents) and triphenylphosphine (223 mg, 0.85 mmol, 6 equivalents). The mixture was stirred at room temperature for 48 h and then the product was precipitated by the addition of a 0.05 m solution of sodium perchlorate (174 mg, 1.42 mmol, 10 equivalents) in acetonitrile (30 mL). The precipitate was centrifuged at 4 °C, washed with cold acetonitrile (20 mL) 3 times and dried under reduced pressure to give a sodium salt of dinucleotide *P*‐imidazolide Im‐pA_m_pG (114 mg, 0.111 mmol, 78%), which was used for coupling reaction without additional purification. Step 2: Im‐pA_m_pG (21.2 mg, 26.5 µmol) was added to a solution of 2′‐*O*/3′‐*O*‐carbamoyl‐*N*7‐methylguanosine 5′‐*O*‐diphosphate^[^ ^19^ ^]^ (L2_N_‐m^7^GDP, 23.7 mg, 31.8 µmol, 1.2 equivalents) and ZnCl_2_ (57.7 mg, 424 µmol, 16 equivalents) in DMSO (530 µL) and the mixture was stirred for 3 h. The reaction was quenched by the addition of a solution of Na_2_EDTA (158 mg, 424 µmol, 16 equivalents) and NaHCO_3_ (79 mg) in water (7.9 mL), and the product was isolated by ion‐exchange chromatography on DEAE Sephadex (gradient elution 0–1.2 m TEAB) to afford after evaporation the triethylammonium salt of regioisomeric mixture of 1 and 2 (L2_N_‐m^7^GpppA_m_pG, 490 mOD_260nm_, 26.5 mg, 15.3 µmol, 58%). RP‐HPLC (conditions A): R_t_ = 6.587, 6.679 min; HR‐MS ESI: m/z calc. for C_35_H_48_N_17_O_25_P_4_^−^ [M‐H]^−^: 1230.19632, found: 1230.19631.

### Synthesis of Cap Analog **3** (m^7^Gppp^L3N^A_m_pG)

Step1: Synthesis of the dinucleotide was performed on a solid support loaded with 5′‐*O*‐DMT‐2′‐*O*‐TBDMS‐rG^iBu^ (162 mg, loading 308 µmol g^−1^, PrimerSupport 5G, GE Healthcare) using a ÄKTA Oligopilot plus 10 synthesizer (GE Healthcare). In the coupling step, 3.0 equivalents of 5′‐*O*‐DMT‐2′‐*O*‐Me‐*N*6‐phenoxyacetyl‐*N*6‐phthalimidopropyl‐adenosine 3′‐*O*‐phosphoramidite (obtained as described earlier^[^ ^22^ ^]^) or biscyanoethyl phosphoramidite and 0.30 m 5‐(benzylthio)−1‐*H*‐tetrazole in acetonitrile were recirculated through the column for 15 min. A solution of 3% (v/v) dichloroacetic acid in toluene was used as a detritylation reagent, 0.05 m iodine in pyridine/water (9:1) for oxidation, 20% (v/v) *N*‐methylimidazole in acetonitrile as Cap A and a mixture of 40% (v/v) acetic anhydride and 40% (v/v) pyridine in acetonitrile as Cap B. After the last cycle of synthesis, dinucleotides, still attached to the solid support, were treated with 20% (v/v) diethylamine in acetonitrile to remove 2‐cyanoethyl protecting groups. Finally, the solid support was washed with acetonitrile and dried with argon. The product was cleaved from the solid support and deprotected with AMA (methylamine/ammonium hydroxide 1:1 (v/v), 5 mL, 30 °C, 3 h), evaporated to dryness and redissolved in DMSO (100 µL). The TBDMS groups were removed using triethylammonium trihydrofluoride (TEA·3HF; 125 µL, 65 °C, 3 h), and then the mixture was cooled down and diluted with 0.25 m NaHCO_3_ in water (20 mL). The product was isolated by ion‐exchange chromatography on DEAE Sephadex (gradient elution 0–0.7 m TEAB) to afford after evaporation triethylammonium salt of dinucleotide 6 (p^L3N^A_m_pG; 400 mOD_260nm_, 14.8 µmol). *RP‐HPLC* (conditions A): *R*_t_ = 9.200 min; HR‐MS ESI: m/z calc. for C_24_H_34_N_11_O_14_P_2_^−^ [M‐H]^−^: 762.17674, found: 762.17676; Step 2: The dinucleotide **6** (p^L3N^A_m_pG; 400 mOD_260nm_, 14.8 µmol) and *P*‐imidazolide of 7‐methylguanosine 5′‐*O*‐diphoshate^[^ ^34^ ^]^ (m^7^GDP‐Im, 18.5 mg, 29.5 µmol, 2 equivalents) were suspended in DMSO (300 µL) and ZnCl_2_ (40.1 mg, 295 µmol, 20 equivalents) was added. The reaction mixture was stirred for 3 days and then a solution of Na_2_EDTA (137 mg, 369 µmol, 25 equivalents) and NaHCO_3_ (69 mg) in water (6.9 mL) was added. The product was isolated by ion‐exchange chromatography on DEAE Sephadex (gradient elution 0–1.0 m TEAB) to afford after evaporation the triethylammonium salt of **3** (m^7^Gppp^L3N^A_m_pG, 340 mOD_260nm_, 16.0 mg, 10.6 µmol, 72%). RP‐HPLC (conditions A): R_t_ = 7.759 min. HR‐MS ESI: m/z calc. for C_35_H_49_F_3_N_16_O_24_P_4_^−^ [M‐H]^−^: 1201.20616, found: 1201.20370.

### Synthesis of Cap Analog **4** (m^7^GpppA_L3N_pG)

Step 1: The synthesis was performed analogously to the procedure described above for **6** (p^L3N^A_m_pG), using 2′‐*O*‐phthalimidopropyl adenosine phosphoramidite obtained as described in the literature.^[^ ^24^ ^]^ The procedure yielded triethylammonium salt of **7** (pA_L3N_pG, 1350 mOD_260nm_, 49.8 µmol). RP‐HPLC (conditions B): *R*_t_ = 5.553 min; HR‐MS ESI: m/z calc. for C_23_H_32_N_11_O_14_P_2_^−^ [M‐H]^−^: 748.16109, found: 748.16118; Step 2: The dinucleotide **7** (pA_L3N_pG; 1350 mOD_260nm_, 49.8 µmol) and *P*‐imidazolide of 7‐methylguanosine 5′‐*O*‐diphoshate^[^ ^34^ ^]^ (m^7^GDP‐Im, 62.6 mg, 99.6 µmol, 2 equivalents) were suspended in DMF (1.0 mL) and ZnCl_2_ (136 mg, 1.0 mmol, 20 equivalents) was added. The reaction mixture was stirred for 5 days and then a solution of Na_2_EDTA (426 mg, 1.15 mmol, 23 equivalents) and NaHCO_3_ (213 mg) in water (21 mL) was added. The product was isolated by ion‐exchange chromatography on DEAE Sephadex (gradient elution 0–0.9 m TEAB) to afford after evaporation the triethylammonium salt of **4** (m^7^GpppA_L3N_pG, 727 mOD_260nm_, 22.7 µmol, 46%). RP‐HPLC (conditions B): *R*_t_ = 5.012 min; HR‐MS ESI: m/z calc. for C_34_H_47_N_16_O_24_P_4_^−^ [M‐H]^−^: 1187.19051, found: 1187.19086.

### Synthesis of Cap Analog 5 (L2_N_‐GpppApG)

The synthesis was performed analogously to the procedure described above for **1** and **2** (L2_N_‐m^7^GpppA_m_pG), using Im‐pA_m_pG (30.0 mg, 33.2 µmol), ZnCl_2_ (72.3 mg, 532 µmol, 16 equivalents) and L2_N_‐GDP (48.4 mg, 66.4 µmol, 2 equivalents, obtained as described previously^[^ ^19^ ^]^) instead of L2_N_‐m7GDP. The procedure yielded triethylammonium salt of 5 as a regioisomeric mixture (795 mOD_260nm_, 20.3 µmol, 61%). RP‐HPLC (conditions A): *R*_t_ = 6.959 min; HR‐MS ESI: m/z calc. for C_34_H_46_N_17_O_25_P_4_^−^ [M‐H]^−^: 1216.18067, found: 1216.18194.

### Synthesis of Photoactivatable Cap Analogs **1a–4a** and **1b–5b**—Labeling of Trinucleotide Cap Analogs **1**–**4**—General Procedure

The trinucleotide cap analog (1 equivalent) was dissolved in DMSO (to 0.05 m) with triethylamine (2 equivalents) and a 0.4 m solution of the NHS ester in DMSO (2 equivalents) was added. The mixture was vortexed for 24 h, then diluted with 0.05 m CH_3_COONH_4_ buffer pH 5.9 (10 times volume of DMSO) and extracted with ethyl acetate. The product was isolated from the aqueous phase by RP HPLC (a linear gradient of acetonitrile in aqueous CH_3_COONH_4_ buffer pH 5.9) to give—after repeated freeze–drying from water—an ammonium salt of trinucleotide cap **1a–4a** or **1b–5b**. The synthesis scales, yields, HPLC, and HRMS data for particular cap analogs are summarized in **Table** **1**.

**Table 1:** Summary of the synthesis scales, yields, HPLC, and HRMS data for labeled trinucleotide cap analogs.

No	Cap analog	Label	Scale^a)^ [µmol]	Yield^b)^ [µmol]	RP HPLC R_t_ ^c)^ [min]	m/z _calcd._	m/z _found_
**1a**	L2_N_‐m^7^GpppA_m_pG (**1+2**)	Diazirine	4.69	0.95	10.522	1442.21607	1442.21680
**2a**				1.54	10.696	1442.21607	1442.21316
**3a**	m^7^Gppp^L3N^A_m_pG (**3**)	Diazirine	5.31	2.63	13.598	1413.22590	1413.22499
**4a**	m^7^GpppA_L3N_pG (**4**)	Diazirine	7.57	4.71	10.566	1399.21025	1399.21004
**1b**	L2_N_‐m^7^GpppA_m_pG (**1+2**)	6‐Nitroveratryl alcohol	7.50	2.91	7.855	1497.27061	1497.27065
**2b**				1.55	7.956	1497.27061	1497.27159
**3b**	m^7^Gppp^L3N^A_m_pG (**3**)	6‐Nitroveratryl alcohol	5.31	2.39	8.408	1468.28044	1468.28101
**4b**	m^7^GpppA_L3N_pG (**4**)	6‐Nitroveratryl alcohol	7.57	4.53	7.566	1454.26479	1454.26525
**5b**	L2_N_‐GpppA_m_pG (**5**)	6‐Nitroveratryl alcohol	6.21	3.35	7.775	1483.25496	1483.25446

^a)^ Based on the amount of cap analog used for the synthesis;

^b)^ After RP HPLC;

^c)^ Conditions B – see General information.

### Synthesis of Photoaffinity Labeling Probe **PAL‐1a**

To an aqueous solution of cap analog **1a** (5 mm, 284 µL), a 50 mm solution of sodium periodate in 1 m sodium acetate buffer pH 5.4 (114 µL, 2 equivalents) was added and the mixture was stirred at RT in darkness for 20 min. Then, a 75 mm solution of ethylenediamine in 1 m sodium acetate buffer pH 5.4 (95 µL, 5 equivalents) was added, followed by 75 mm solution of NaBH_3_CN in DMF (379 µL, 20 equivalents), and the reaction mixture was stirred at RT for 1 h. The product was isolated by semi‐preparative RP HPLC (a linear gradient 0–30% of acetonitrile in 50 mm ammonium acetate pH 5.9 within 90 min) in 32% yield. RP‐HPLC (conditions C): *R*_t_ = 6.795 min; HR‐MS ESI: m/z calc. for C_46_H_57_F_3_N_21_O_24_P_4_^−^ [M‐H]^−^: 1468.27934, found: 1468.27940.

### Synthesis of Fluorescent Photoaffinity Labeling Probes—General Procedure A: NHS Tagging

To a 5 mm solution of cap analog **PAL‐1a** in water (46 µL), TEA (1 uL), and a 200 mm solution of NHS ester in DMSO (5.7 µL, 10 equivalents) were added and the reaction mixture was stirred at RT for 1 h. The product was isolated by RP HPLC on analytical column (conditions B, see General Information).

### Synthesis of Fluorescent Photoaffinity Labeling Probes—General Procedure B: HATU Tagging

To a pre‐cooled (−22 °C) 200 mm solution of carboxylic acid in DMF (1.1 µL, 2 equivalents of R‐COOH), pre‐cooled solutions of DIPEA (0.6 µL, 400 mm, 2 equivalents) and HATU (0.6 µL, 400 mm, 2 equivalents) in DMF were added and the mixture was kept at −22 °C for 30 min. Then, the activated acid was added to a 2.5 mm solution of **PAL‐1a** in 250 mm phosphate buffer pH 7 / DMSO 1:1_v/v_ mixture (46 µL) and the reaction mixture was stirred at RT for 1 h. The product was isolated by RP HPLC on analytical column (conditions B, see General Information).

**PAL‐1a‐FAM** and **PAL‐1a‐TAMRA** were synthesized according to the General Procedure A. **PAL‐1a‐sCy5** was synthesized according to the General Procedure B.

### Synthesis of 6‐Nitroveratryl Alcohol NHS‐Ester

The 6‐nitroveratryl alcohol derivative was synthesized according to the literature protocols for analogous compounds,^[^ ^28^, ^35^ ^]^ starting from vanillin.


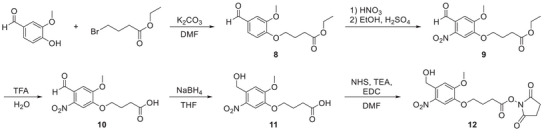


### Synthesis of 3‐Methoxy‐4‐[3‐(Ethoxycarbonyl)Propyloxy]Benzalhedyde (**8**)

Vanillin (5.00 g, 32.9 mmol), ethyl 4‐bromobutyrate (4.95 mL, 32.9 mmol), and K_2_CO_3_ (9.10 g, 65.7 mmol) were suspended in DMF (33 mL) and stirred at room temperature for 24 h. The mixture was then poured into water (500 mL) and stirred for 2 h. The precipitate was filtered off and dried under reduced pressure to give compound **8** (9.02 g, 32.8 mmol, 99%) as a white solid. ^1^H NMR (500 MHz, d‐DMSO, 25 °C): δ = 9.83 (s, 1H), 7.53 (dd, ^3^J_H,H_ = 8.2 Hz, ^3^J_H,H_ = 1.8 Hz, 1H), 7.39 (d, ^3^J_H,H_ = 1.8 Hz, 1H), 7.16 (d, ^3^J_H,H_ = 8.2 Hz, 1H), 4.10 (t, ^3^J_H,H_ = 6.5 Hz, 2H), 4.06 (q, ^3^J_H,H_ = 7.1 Hz, 2H), 3.83 (s, 3H), 2.46 (t, ^3^J_H,H_ = 7.3 Hz, 2H), 2.00 (m, 2H), 1.18 (t, ^3^J_H,H_ = 7.1 Hz, 3H) ppm; ^13^C NMR (126 MHz, d‐DMSO, 25 °C): δ = 191.4, 172.4, 153.4, 149.3, 129.7, 126.0, 112.1, 109.7, 67.5, 59.9, 55.6, 30.0, 24.0, 14.1 ppm.

### Synthesis of 3‐Methoxy‐4‐[3‐(Ethoxycarbonyl)Propyloxy]−6‐Nitrobenzalhedyde (**9**)

To a concentrated HNO_3_ (45 mL) cooled in an ice bath (2–3 °C), the compound **8** (4.50 g, 16.9 mmol) was added in small portions over 20 min. Then the ice bath was removed, and the mixture was stirred at room temperature. After 3 h the mixture was poured into water/ice (ca. 500 mL) and the precipitate was filtered, washed with water (100 mL) and dried under reduced pressure. The yellow solid was dissolved in ethanol (100 mL), 95% of H_2_SO_4_ (200 µL) was added and the mixture was refluxed for 5 h and left to cool down. The solvent was evaporated to dryness, the solid was redissolved in DCM (10 mL) and flash‐chromatographed on silica‐gel column (80 g, elution with 100% DCM) in two portions, to give – after evaporation of appropriate fractions – compound **9** (1.99 g, 6.38 mmo, 38%) as a yellow solid. ^1^H NMR (500 MHz, CDCl_3_, 25 °C): δ = 10.44 (s, 1H), 7.61 (s, 1H), 7.41 (s, 1H), 4.22 (t, ^3^J_H,H_ = 5.9 Hz, 2H), 4.17 (q, ^3^J_H,H_ = 6.9 Hz, 2H), 4.00 (s, 3H), 2.56 (t, ^3^J_H,H_ = 6.9 Hz, 2H), 2.23 (m, 2H), 1.27 (m, 3H) ppm; ^13^C NMR (126 MHz, CDCl_3_, 25 °C): δ = 187.8, 172.7, 153.5, 151.7, 143.8, 125.5, 109.9, 108.1, 68.7, 60.7, 56.6, 30.4, 24.1, and 14.2 ppm.

### Synthesis of 3‐Methoxy‐4‐(3‐Carboxypropyloxy)−6‐Nitrobenzalhedyde (**10**)

To a solution of compound **9** (1.98 g, 6.36 mmol) in water (15.9 mL), TFA (1.59 mL, 20.8 mmol) was added and the mixture was stirred at 90 °C for 3 h. Then the mixture was cooled down and the precipitated was filtered off, washed with water and dried under reduced pressure to give compound **10** (1.69 g, 5.97 mmol, 94%) as a yellow solid. ^1^H NMR (500 MHz, d‐DMSO, 25 °C): δ = 12.20 (s, 1H), 10.14 (s, 1H), 7.71 (s, 1H), 7.37 (s, 1H), 4.20 (t, ^3^J_H,H_ = 6.5 Hz, 2H), 3.95 (s, 3H), 2.40 (t, ^3^J_H,H_ = 7.3 Hz, 2H), 1.98 (m, 2H) ppm; ^13^C NMR (126 MHz, d‐DMSO, 25 °C): δ = 188.6, 174.0, 152.7, 151.2, 143.6, 124.7, 110.2, 108.3, 68.5, 56.5, 29.9, and 23.9 ppm.

### Synthesis of 3‐Methoxy‐4‐(3‐Carboxypropyloxy)−6‐Nitrobenzyl Alcohol (**11**)

Compound **10** (1.68 g, 5.93 mmol) was suspended in THF (33 mL) at 0 °C and NaBH_4_ (0.34 g, 8.90 mmol) was added in small portions over 10 min. After 24 h the solvent was evaporated, and the residue was dissolved in water and acidified with 35% HCl. The formed precipitate was filtered and evaporated to dryness from methanol to give compound **11** (1.54 g, 5.41 mmol) as a pale‐yellow solid. ^1^H NMR (500 MHz, d‐DMSO, 25 °C): δ = 12.17 (s, 1H), 7.67 (s, 1H), 7.39 (s, 1H), 5.57 (m, 1H), 4.82 (m, 2H), 4.07 (t, ^3^J_H,H_ = 6.5 Hz, 2H), 3.91 (s, 3H), 2.39 (t, ^3^J_H,H_ = 7.3 Hz, 2H), 1.96 (m, 2H) ppm; ^13^C NMR (126 MHz, d‐DMSO, 25 °C): δ = 174.0, 153.7, 146.1, 138.4, 134.2, 109.7, 109.0, 67.9, 60.1, 56.1, 30.0, 24.1 ppm.

### Synthesis of 3‐Methoxy‐4‐(3‐Succinimidylcarboxypropyloxy)−6‐Nitrobenzyl Alcohol (**12**)

Compound **11** (100 mg, 351 µmol) was dissolved in DMF (3.5 mL) and *N*‐hydroxysuccinimide (80.7 mg, 701 µmol), triethylamine (98 µL, 701 µmol), and 1‐ethyl‐3‐(3‐dimethylaminopropyl)carbodiimide (EDC, 134.4 mg, 701 µmol) were added. After 2 h the mixture was diluted with DCM (30 mL) and washed with 1% HCl_aq_ (30 mL). The product was isolated from the organic phase by flash chromatography (4 g silica gel column, elution 0–100% ethyl acetate in hexane), to give – after evaporation of appropriate fractions – compound **12** (95.0 mg, 248 µmol, 71%) as a yellow solid. ^1^H NMR (500 MHz, d‐DMSO, 25 °C): δ = 7.68 (s, 1H), 7.40 (s, 1H), 5.58 (t, ^3^J_H,H_ = 5.4 Hz, 1H), 4.83 (d, ^3^J_H,H_ = 5.4 Hz, 2H), 4.14 (t, ^3^J_H,H_ = 6.4 Hz, 2H), 3.92 (s, 3H), 2.87 (t, ^3^J_H,H_ = 7.4 Hz, 2H), 2.82 (m, 4H), 2.10 (m, 2H) ppm.

### Synthesis and Purification of Short‐Capped RNAs

All capped RNAs were obtained by in vitro transcription on the template which contained a ϕ6.5 promoter sequence (underlined) followed by a 35‐nt‐long sequence (CA GTAATA CGACTC ACTATA GGGGAA GCGGGC ATGCGG CCAGCC ATAGCC GATCA).^[^ ^31^ ^]^ The transcription reaction (100 µL) was carried out under the following conditions: 20 µL 5x Transcription Buffer (ThermoFisher Scientific), 20 mm MgCl_2_, 1 µm oligonucleotide DNA template, 5 mm NTPs (except for GTP, of which the concentration was 2 mm), 5 mm of cap analog, 1U µL^−1^ RiboLock RNase inhibitor (ThermoFisher Scientific), 10 µL of T7 RNA polymerase (1 mg mL^−1^) and MQ water to the final volume. The reaction was incubated for 4 h at 37 °C, followed by 0.5 h incubation with 1U µL^−1^ DNase I (ThermoFisher Scientific) at 37 °C. The reaction was quenched by the addition of 6 µL 0.5 m Na_2_EDTA (pH 8). The obtained RNAs were purified using Monarch RNA Cleanup Kit (50 µg) followed by RP HPLC purification using Phenomenex Clarity Oligo‐RP column (150 × 4.6 mm, 3 µm), HPLC method: eluent A: 100 mm TEAAc, eluent B: 200 mm TEAAc/CAN 1:1_v/v_; 0–22% of eluent B in 20 min. Fractions thus obtained were then freeze‐dried. The quality of RNA was checked on 15% acrylamide/7 m urea/TBE gel with SYBR Gold (Invitrogen) staining.

### Synthesis and Purification of Firefly Luciferase mRNAs

Capped mRNAs were obtained by in vitro transcription on the template that encode Firefly Luciferase (Fluc). The Fluc gene was purchased from Invitrogen (Thermo Fisher Scientific) and cloned into the plasmid pJet vector (Thermo Fisher Scientific). The DNA template for IVT was prepared by AarI digestion (Thermo Fisher Scientific). The transcription reactions were carried out under conditions presented in **Table** **2**. The final volume of all reactions was adjusted with MQ water. Reaction mixtures were incubated for 4 h at 37 °C, followed by 0.5 h incubation with 1U µL^−1^ DNase I (ThermoFisher Scientific) at 37 °C. The reactions were quenched by the addition of 0.1 V 0.5 m Na_2_EDTA (pH 8). The obtained mRNAs were purified using Monarch RNA Cleanup Kit (500 µg) followed by RP HPLC purification using Phenomenex PolimerX column, (150 × 4.1 mm, 3 µm), HPLC method: eluent A: 100 mm TEAAc, eluent B: 200 mm TEAAc/ACN 1:1_v/v_; 0–32.5% of eluent B in 25 min. Fractions thus obtained were then precipitated as sodium salts (3 m NaOAc pH 5.2, isopropanol/ 80% EtOH) and dissolved in water. The quality of mRNAs was checked on 1% agarose gel electrophoresis (TBE, 30 min, 120 V).

**Table 2:** Concentration of the reagents used for in vitro transcription reactions leading to Firefly luciferase mRNAs.

COMPONENT	M^7^GPPPA_m_PG	1B	2A	UNIT
5X TRANSCRIPTION BUFFER	1x	1x	1x	–
MGCL_2_	20	20	24	M
PJET_FLUCA90 DNA TEMPLATE	50	50	50	ng µL^−1^
ATP/CTP/M^1^Ψ	6	6	3	ng µL^−1^
GTP	4	4	2	mM
CAP ANALOG	10	10	6	mM
RIBOLOCK RNASE INHIBITOR	1	1	1	U µL^−1^
PPASE	0.002	0.002	0.002	U µL^−1^
T7 RNA POLYMERASE	0.125	0.125	0.250	mg mL^−1^
FINAL VOLUME	50	50	30	µL

### Protein Expression and Purification

*eIF4E*: Murine eIF4E was expressed in *Escherichia coli*, isolated from insoluble fraction and refolded as described earlier.^[^ ^36^ ^]^ *Dcp2‐Dcp1‐PNRC2*: Human decapping complex PNRC2‐Dcp1/Dcp2 was expressed in *Escherichia coli* and purified as described previously.^[^ ^37^ ^]^ *hDcp2*: Human Dcp2 was expressed in *E. coli* and purified as described previously.^[^ ^38^ ^]^

### UV Activation Kinetics

A 10 mm stock solution of compound **4b** in water was diluted with 500 µL of appropriate buffer (phosphate pH 7.0, HEPES pH 8.0, or TrisHCl pH 8.0) in a UV quartz cuvette. The solution was irradiated with UV Power LEDs (369 nm, 2×3 W, ca. 2 cm from the sample) for 60 s and the cuvette was immediately placed in a UV–vis spectrophotometer. UV–vis spectra were recorded every 1–5 min over 1 h. Absorption values at several wavelengths (280, 315, 360, and 390 nm) were plotted as a function of time and an exponential function was fitted using GraphPad Prism 8.0.1 to estimate the half‐life of the reaction.

### UV‐Crosslinking of Trinucleotide Cap Analogs to eIF4E Protein

eIF4E stock solution (ca. 200 µm) was diluted with an appropriate 0.1 m buffer (Bis‐Tris pH 5.5, Bis‐Tris pH 6.5, Bis‐Tris pH 7.0, Tris pH 7.5, Tris pH 8.0, HEPES pH 7.2, HEPES pH 8.0, or Bicine pH 8.5) to a final concentration of 10 µm and mixed with a 1.7 mm solution of trinucleotide cap analog in 19:1 ratio (0.5 µL of eIF4E + 0.5 µL of cap). The negative control samples “UV(‐)” (2 µL) were collected for PAGE analysis (and stored at RT without additional protection from daylight throughout the experiment) and the rest of the solution was transferred into a 96‐well plate (Swissci 3 lens crystallization plate), incubated for 15 min, and then irradiated with UV tube (6 W, 365 nm, VL‐6.LC, Vilber) for 30 min. All the samples were analyzed by SDS PAGE on 12% gel, stained with Bio‐Safe Coomasie G‐250 (Bio‐Rad), and visualized using GelDoc Go Gel Imaging System (Bio‐Rad). Selected samples were analyzed by HPLC‐MS.

### UV‐Crosslinking of Capped RNA Fragments to Model Proteins

The RNA 5 µm stock solutions (2 µL) were mixed with protein solutions (8 µL; 27.7 µm eIF4E, 31.0 µm Dcp2, 20 µm BSA, or 20 µm ovalbumin) in PCR tubes and incubated for 15 min. The tubes were then irradiated with UV Power LEDs (369 nm, ca. 1 cm from the samples) for 30 min (cumulative time: for 6‐nitroveratryl derivatives 6 cycles of [5 min UV + 5 min dark]; for diazirine derivatives 3 cycles of [10 min UV + 5 min dark]). The negative control samples “UV(‐)” were prepared simultaneously with the irradiated samples and stored at RT without additional protection from daylight throughout the experiment. An aliquot (5 µL) of each sample was analyzed by gel electrophoresis on a 12% acrylamide:bisacrylamide 19:1 / 7 m urea / TBE gel (13 W, 40 min). The RNA was stained with SYBR Gold (Invitrogen) and visualized using a Typhoon FLA 9500 (GE Healthcare). The crosslinking yield was estimated by densitometric analysis of the gels (CLIQS software, TotalLab), as a ratio between the intensity of the band corresponding to RNA‐protein crosslink and the sum of the intensity of bands from crosslinked and free RNAs.

### RNA Decapping Assay

Capped RNAs (0.67 µm) were incubated with hDcp2 (6.7 µm) in 1xNEBuffer 3 (New England BioLabs, 15 µL) for 1 h at 37 °C. The reaction was quenched by freezing in liquid nitrogen. Then, the samples were analyzed by TBE PAGE as described above.

### mRNA Translation Studies in Cultured Mammalian Cells

A549 cells (human epithelial lung carcinoma, ATCC CCL‐185) were cultured in DMEM medium (Gibco) supplemented with 10% FBS (Sigma), GlutaMAX (Gibco), and 1% penicillin/streptomycin (Gibco) at 37 °C with 5% CO_2_. The murine immature dendritic cell line JAWS II (ATCC CRL‐11904) was cultured in RPMI 1640 (Gibco) supplemented with 10% FBS, sodium pyruvate (Gibco), 1% penicillin/streptomycin, and 5 ng mL^−1^ GM‐CSF (PeproTech) at 37 °C with 5% CO_2_. A549 and JAWS II cells (10^4^ per well) were seeded in a 96‐well plate 3 h before transfection in 100 µL of antibiotic‐free medium. Each well was transfected using a mixture of 0.6 µL Lipofectamine MessengerMAX transfection reagent (Invitrogen) and 50 ng of mRNA encoding Firefly luciferase in 10 µL Opti‐MEM (Gibco) for each studied 5′ cap modification. The experiment was conducted over 24 h post‐transfection, with signal collection at 3 time points (4, 8, and 24 h post‐transfection). For each modification and time point in both cell lines, a portion of the cells was exposed to UV radiation. Post‐transfection, the cells were incubated for 30 min at 37 °C with 5% CO_2_, followed by a 15‐min irradiation at 365 nm (UV tube, see General Information). To assess Firefly luciferase expression, plates were centrifuged (1 min, 300 × g, 25 °C), the medium was completely removed, and replaced with 100 µL BrightGlo (Promega) reagent. The cells were incubated for 5 min at each time point and transferred to a white 96‐well plate. Luminescence detection from Firefly luciferase was measured using a Synergy H1 (BioTek) microplate reader.

### Photoaffinity Labeling of HEK293F Cell Extract

HEK293F cell extract (10.715 mg mL^−1^; prepared as described earlier)^[^ ^39^ ^]^ was diluted to 5 mg mL^−1^ with PAL buffer (50 mm HEPES pH 7.4, 100 mm KCl, 0.5 mm EDTA) containing 0.2 mm GTP. An aliquot of the solution (6.4 µL) was incubated with m^7^GpppA_m_pG for 15 min (sample C) and then PAL probe was added to a final concentration of 1 µm (samples A, B, and C). After 15 min incubation at RT, samples A, C, and E were irradiated at 369 nm for 30 min in PCR tubes. All samples were analyzed by SDS‐PAGE on gradient gels (Bio‐Rad 4–20% Mini‐PROTEAN TGX). The gels were imaged for fluorescence of PAL probes (FAM, TAMRA, Cy5) using Typhoon FLA 9500 (GE Healthcare) and then stained with Bio‐Safe Coomasie G‐250 (Bio‐Rad) and visualized using GelDoc Go Gel Imaging System (Bio‐Rad). The composition of each sample is shown in **Table** **3**.

**Table 3:** Composition of the samples used in photoaffinity labeling experiments.

SAMPLE	CELL EXTRACT	M^7^GPPPA_m_PG	PAL PROBE	UV
0	4 mg mL^−1^	–	–	−
A	4 mg mL^−1^	–	1 µm	+
B	4 mg mL^−1^	–	1 µm	−
C	4 mg mL^−1^	10 µm	1 µm	+
E	4 mg mL^−1^	–	–	+

## Conflict of Interest

The authors declare no conflict of interest.

## Supporting information

Supporting Information

## Data Availability

The data that support the findings of this study are available in the supplementary material of this article.
