# Supplementary material for: Photoactivatable mRNA 5′ Cap Analogs for RNA‐Protein Crosslinking
Source: Adv Sci (Weinh). 2024 Jul 24;11(36):2400994. doi: 10.1002/advs.202400994 (PMC11423160; doi:10.1002/advs.202400994)

ADVANCED SCIENCE
Open Access

Supporting Information

for *Adv. Sci.*, DOI 10.1002/advs.202400994

Photoactivatable mRNA 5′ Cap Analogs for RNA-Protein Crosslinking

Marcin Warminski, Katarzyna Grab, Kacper Szczepanski, Tomasz Spiewla, Joanna Zuberek, Joanna Kowalska and Jacek Jemielity*

Supporting information

Photoactivatable mRNA 5' cap analogs for RNA-protein crosslinking

Marcin Warminski, Katarzyna Grab, Tomasz Spiewla, Joanna Kowalska, Jacek Jemielity*

Table of contents

Supplementary figures **2**

Compounds characterization **8**

1+2: L2$_N$-m^{7}GpppA$_m$pG 8
1a: Diaz-L2$_N$-m^{7}GpppA$_m$pG_I1 10
1a_UV: UV-irradiated Diaz-L2$_N$-m^{7}GpppA$_m$pG _I1 13
1b: NVA-L2$_N$-m^{7}GpppA$_m$pG_I1 15
2a: Diaz-L2$_N$-m^{7}GpppA$_m$pG_I2 18
2a_UV: UV-irradiated Diaz-L2$_N$-m^{7}GpppA$_m$pG _I2 21
2b: NVA-L2$_N$-m^{7}GpppA$_m$pG_I2 23
3: m^{7}Gppp^{L3N}A$_m$pG 26
3a: m^{7}Gppp$^{Diaz\text{-}L3N}$A$_m$pG 28
3a_UV: UV-irradiated m^{7}Gppp$^{Diaz\text{-}L3N}$A$_m$pG 31
3b: m^{7}Gppp$^{NVA\text{-}L3N}$A$_m$pG 33
4: m^{7}GpppA$_{L3N}$pG 36
4a: m^{7}GpppA$_{L3N\text{-}Diaz}$pG 38
4b: m^{7}GpppA$_{L3N\text{-}NVA}$pG 41
5: L2$_N$-GpppA$_m$pG 44
5b: NVA-L2N-GpppA$_m$pG 46
PAL-1a: Diaz-L2$_N$-GpppA$_m$pG-EDA 48
6: p^{L3N}A$_m$pG 51
7: pA$_{L3N}$pG 53
8: 3-methoxy-4-[3-(ethoxycarbonyl)propyloxy]benzalhedyde 55
9: 3-methoxy-4-[3-(ethoxycarbonyl)propyloxy]-6-nitrobenzalhedyde 57
10: 3-methoxy-4-(3-carboxypropyloxy)-6-nitrobenzalhedyde 59
11: 3-methoxy-4-(3-carboxypropyloxy)-6-nitrobenzyl alcohol 62
12: 3-methoxy-4-(3-succinimidylcarboxypropyloxy)-6-nitrobenzyl alcohol 64

Supplementary figures

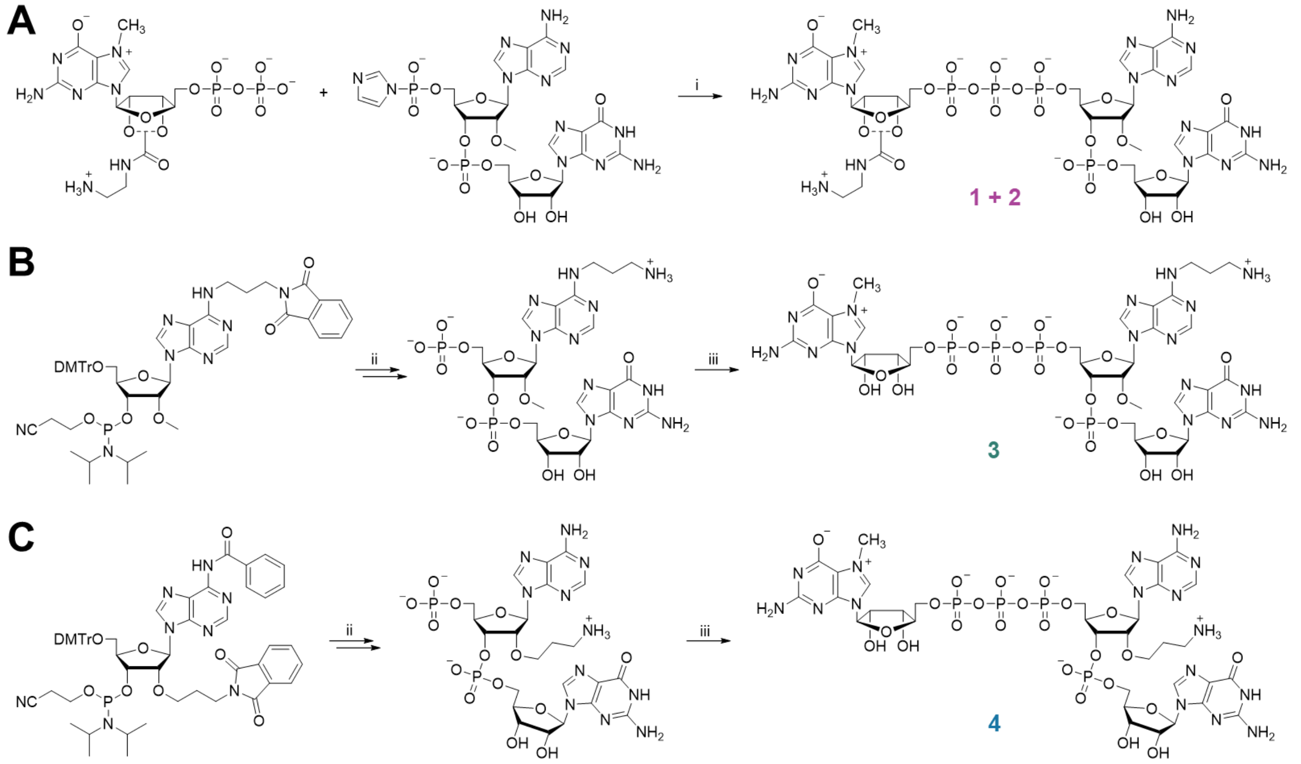

Figure S1. Chemical synthesis of functionalized trinucleotide cap analogs. A) Synthesis of a regioisomeric mixture of analogs **1** and **2**; **B**) Synthesis of analog **3**; **C**) Synthesis of analog **4**. i) $ZnCl_2$, DMSO, RT; ii) solid-phase synthesis on a high-loaded support (see the experimental procedure); iii) m^7GDP-Im, $ZnCl_2$, DMSO or DMF, RT;

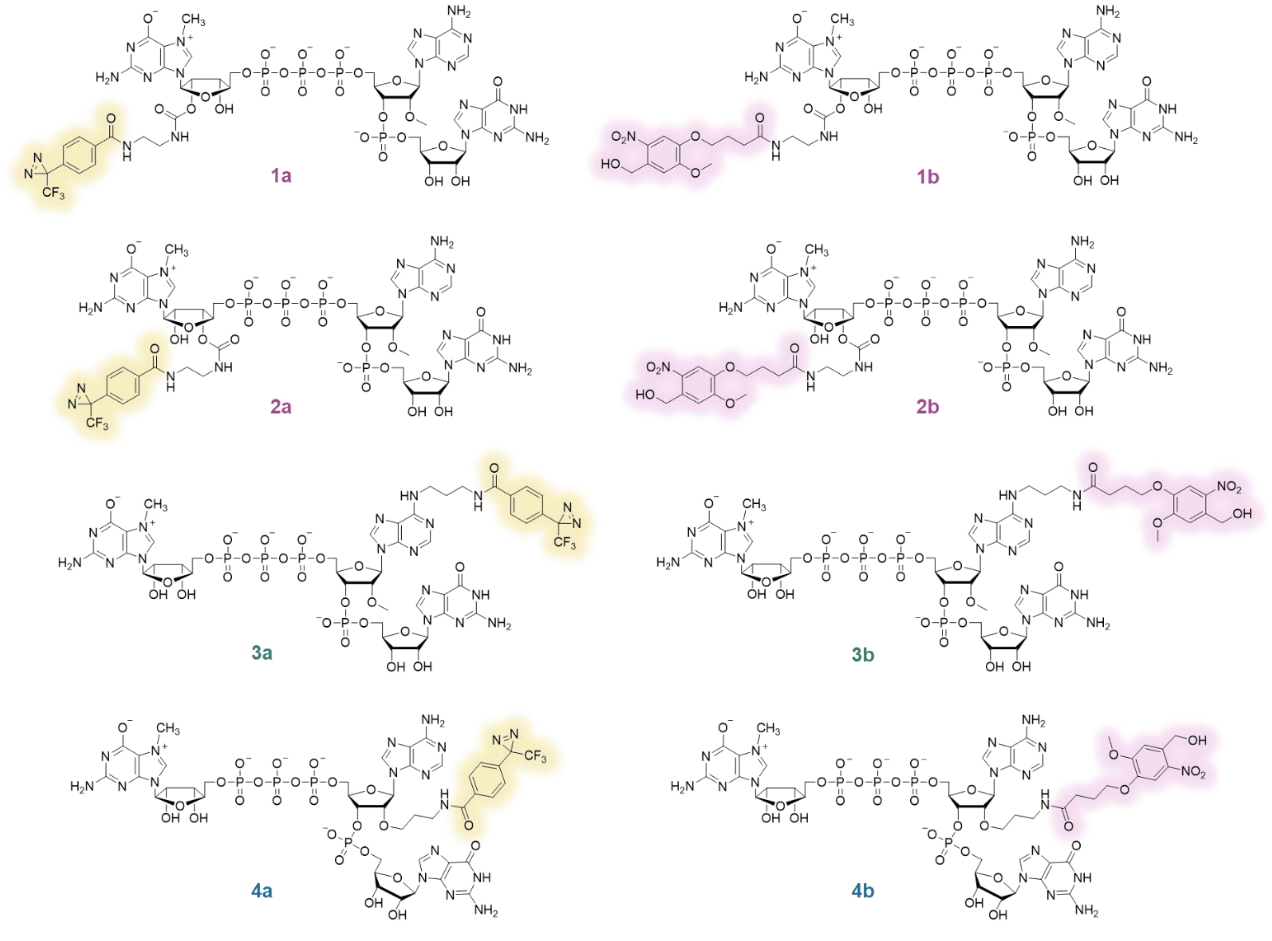

Figure S2. Chemical structures of photoreactive mRNA 5' cap analogs synthesized in this work.

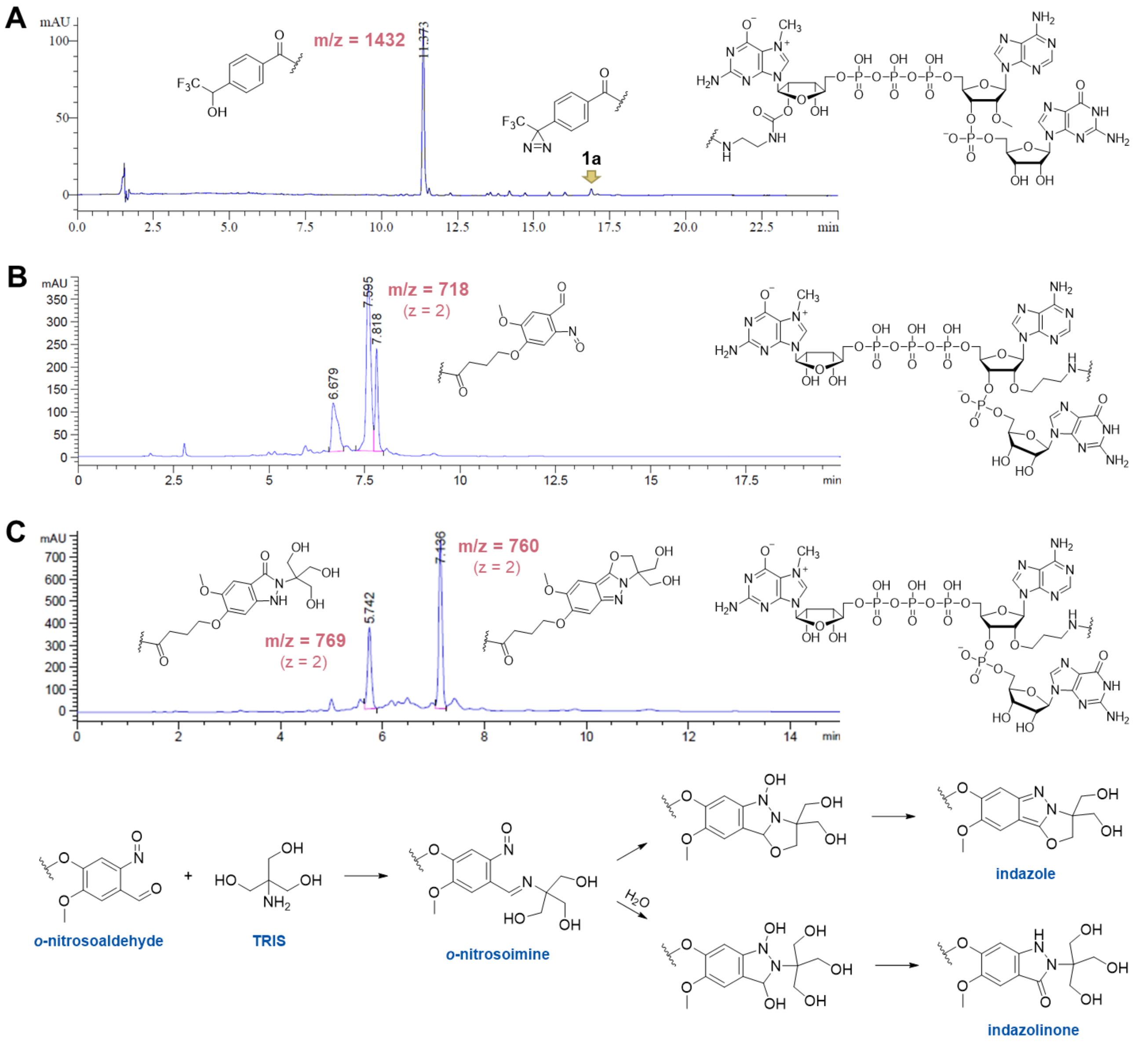

Figure S3. RP-HPLC analysis of photoactivation products. A) RP-HPLC analysis of a **1a** solution in phosphate buffer pH 7 irradiated at 365 nm; **B)** RP-HPLC analysis of a **4b** solution in phosphate buffer pH 7 irradiated at 365 nm; **C)** RP-HPLC analysis of a **4b** solution in TRIS pH 8 irradiated at 365 nm and the two possible reaction mechanisms leading to the observed indazole and indazolinone derivatives.

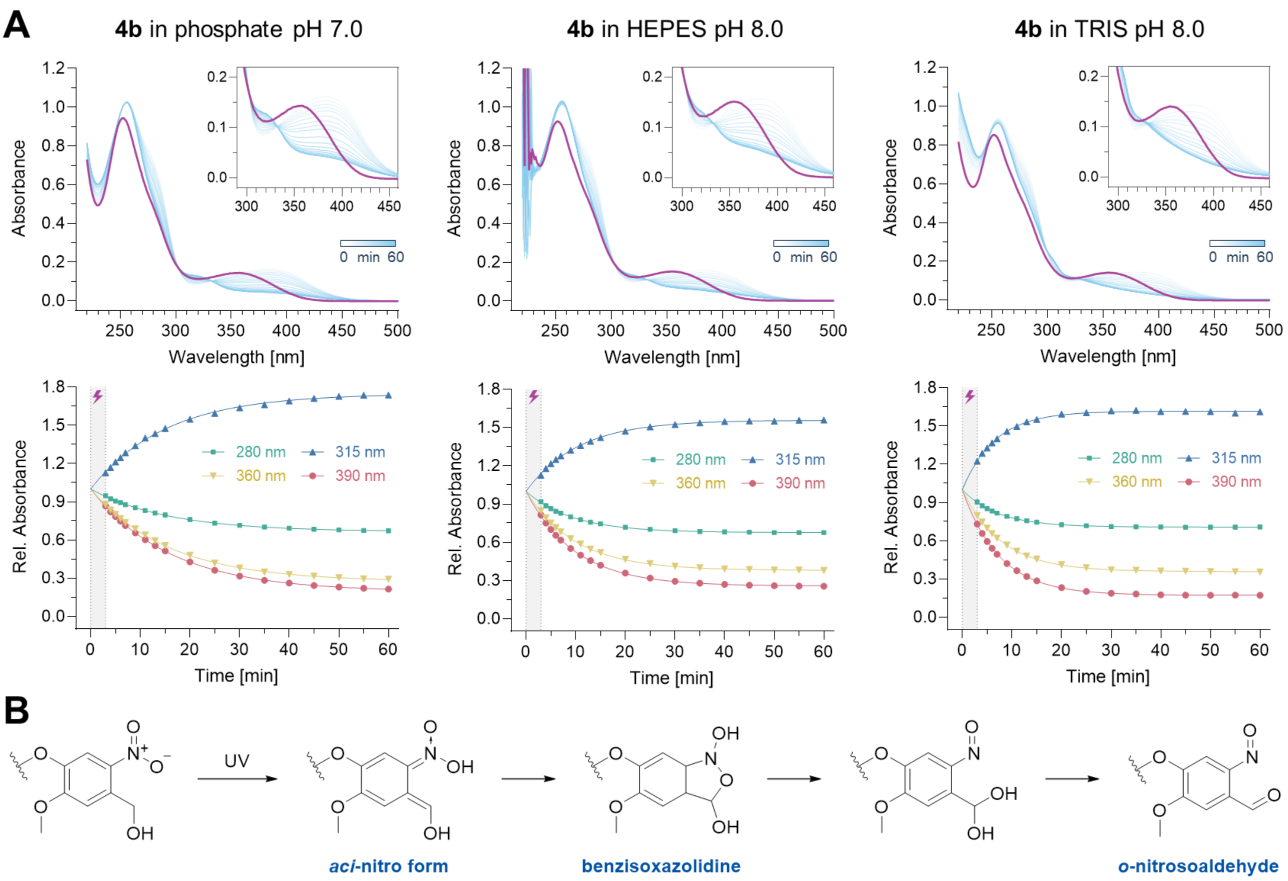

Figure S4. Photochemical properties of cap analog 4b. A) Time-lapsed UV-Vis analysis of the **4b** solutions in various buffers; **B**) Mechanism of photo-induced conversion of nitrobenzyl alcohols intro nitrosoaldehydes.

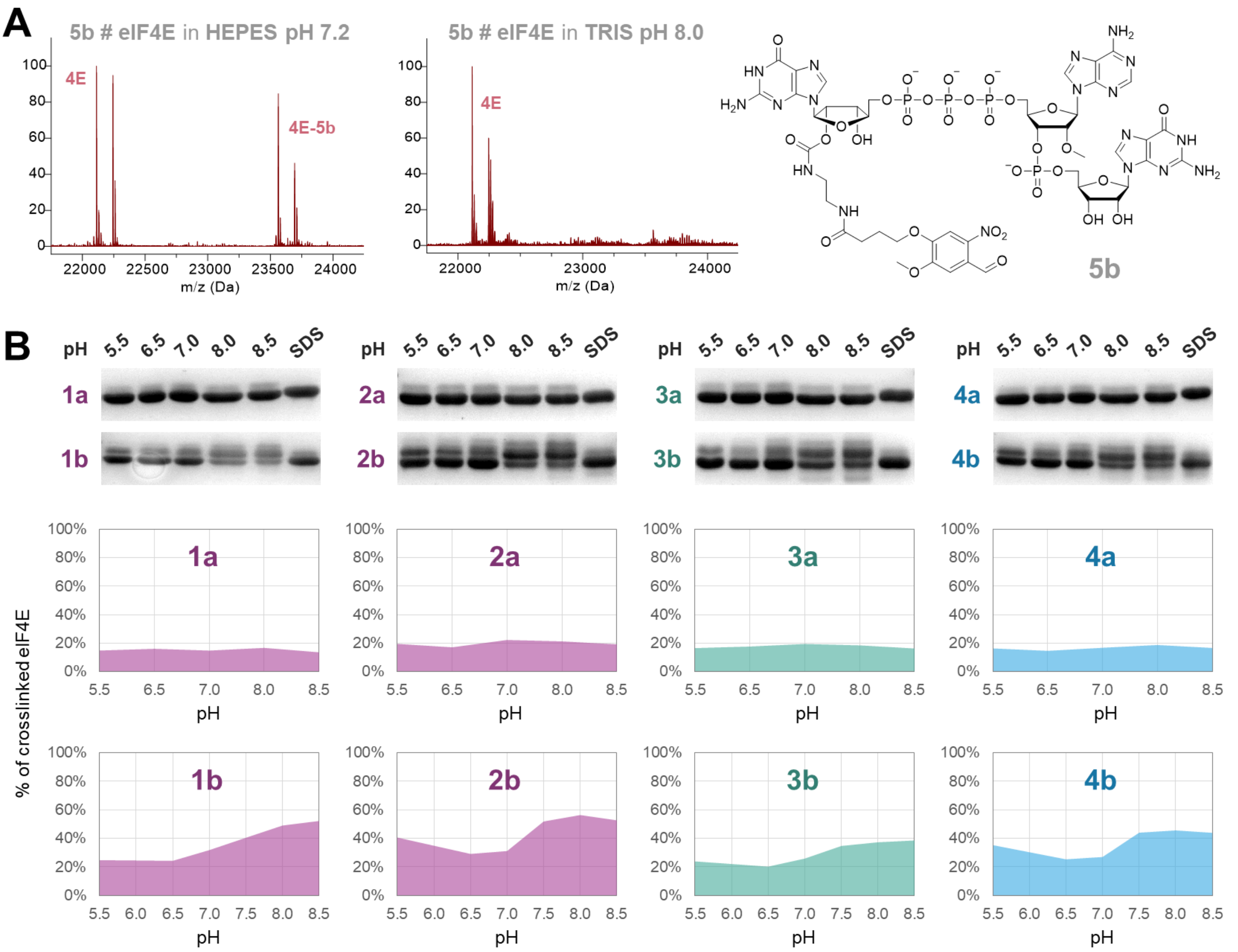

Figure S5. Optimization of pH and buffer type in photocrosslinking reactions between trinucleotide cap analogs 1a–4a and 1b–4b and eIF4E protein. A) Deconvoluted mass spectra of the reaction mixtures. The two peaks of eIF4E protein (M and M+131) result from incomplete processing of the N-terminal Met by E. Coli Methionine Aminopeptidase and are also present in the spectrum of non-irradiated protein. **B)** SDS PAGE analysis of cap-eIF4E photocrosslinking reaction mixtures; Fraction of the crosslinked protein was estimated by densitometric analysis of the bands and plotted against pH value.

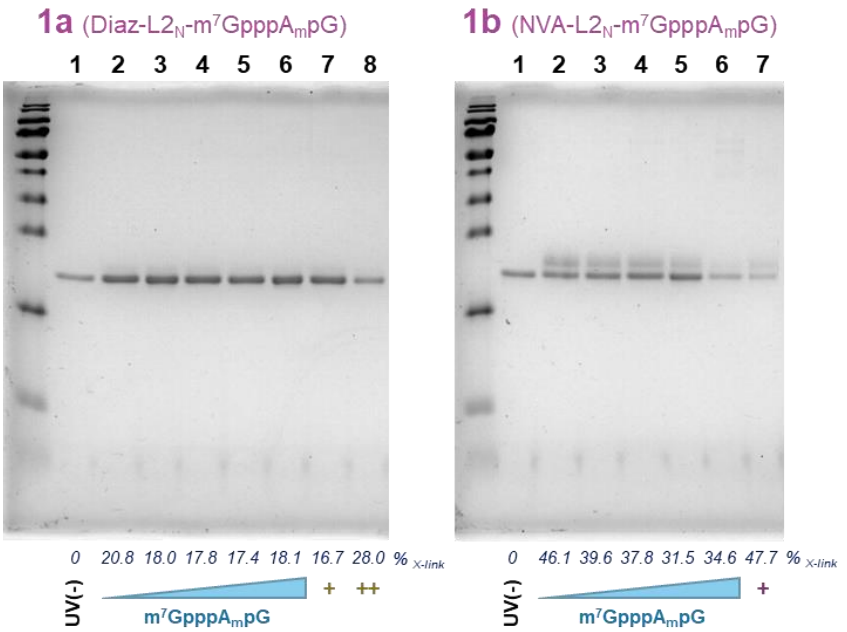

Figure S6. Electrophoretic (SDS PAGE) analysis of the photocrosslinking reaction between photoreactive cap analog and eIF4E protein. Lane 1) No UV irradiation control sample; Lane 2) analog **1a/b** / eIF4E (10:1) irradiated for 15 min; Lanes 3–6) analog **1a/b** / eIF4E / m^{7}GpppA$_m$pG irradiated for 15 min – 0.5, 1, 2, and 10-fold excess of m^{7}GpppA$_m$pG over **1a/b** was used; Lane 7) analog **1a/b** / eIF4E (10:1) irradiated for 15 min followed by addition of a fresh portion of analog **1a/b** (10 eqv.) and irradiation for 15 min; Lane 8) same as lane 7 + one more portion of **1a**;

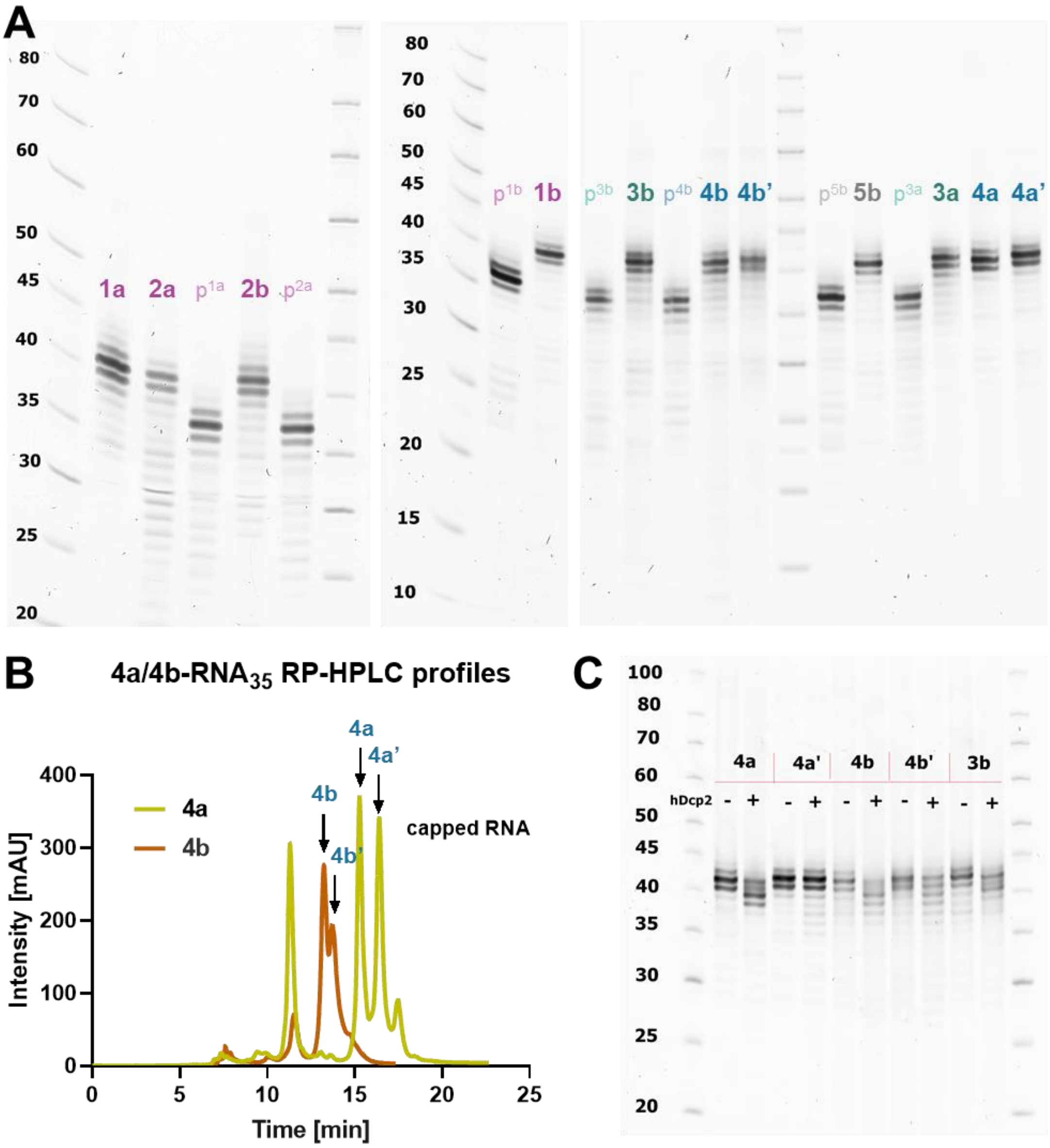

Figure S7. Characterization of short RNAs capped with photoreactive analogs. A) TBE PAGE analysis of RP-HPLC-purified RNAs; **B**) RP-HPLC chromatograms of in vitro transcription products primed with analogs **4a** and **4b**; **C**) TBE PAGE analysis of hDcp2-catalyzed decapping of isomeric RNAs capped with **4a** and **4b** – RNAs with reverse incorporated cap (indicated with 'prime') are degraded slower than the correctly capped RNAs; The RNA sequence according to the DNA template: GGGGA AGCGG GCAUG CGGCC AGCCA UAGCC GAUCA; capped RNAs have an additional modified m7GpppA- at the 5' end.

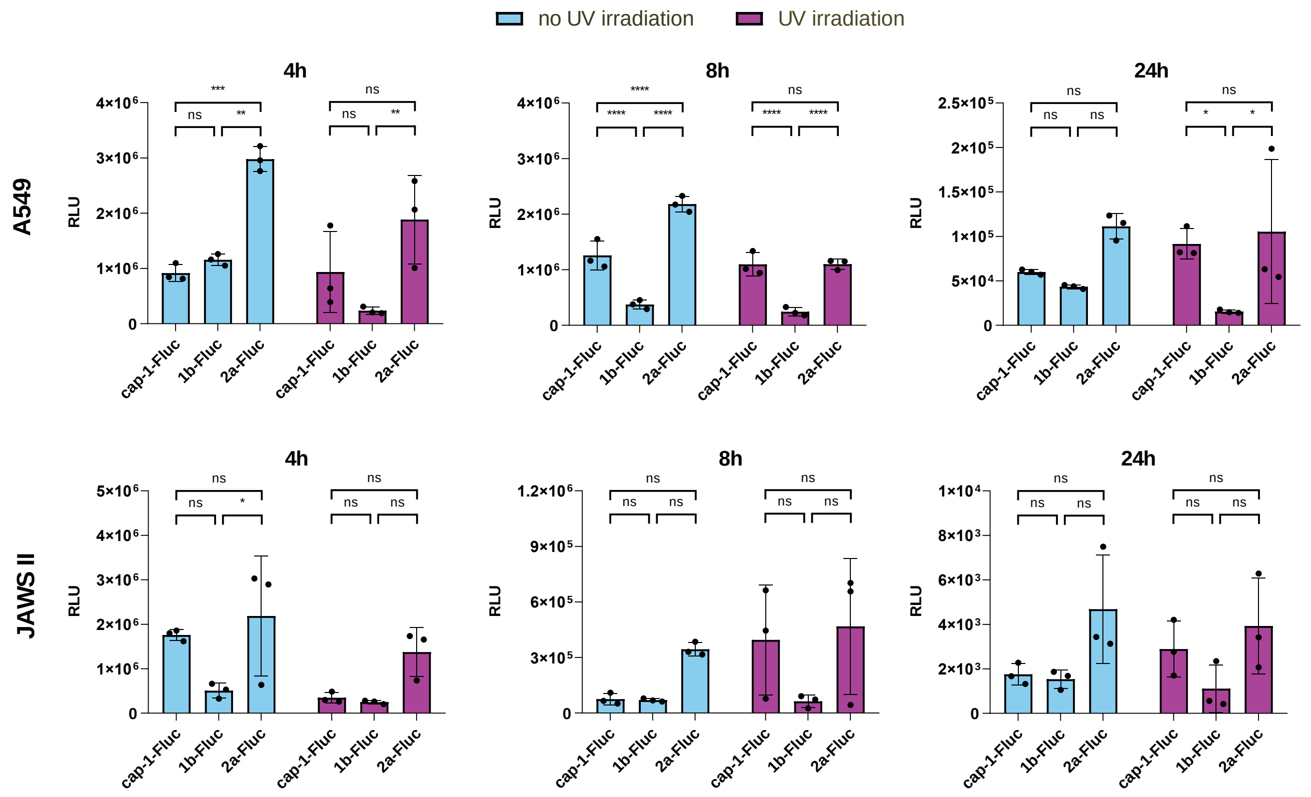

Figure S8. The effect of photoreactive tags on mRNA translation in cultured cells. Firefly luciferase (Fluc)-dependent luminescence of human epithelial lung carcinoma (A549, 10^4 cells per well) and murine immature dendritic (JAWS II, 10^4 cells per well) cells 4 h, 8 h, and 24 h post transfection with 50 ng of Fluc mRNA; data show relative luminescence units (RLU) means ± SD, n = 3, *P < 0.05, **P<0.01, ***P<0.001, ****P < 0.0001, ns – not significant, two-way ANOVA with Tukey's multiple comparison test.

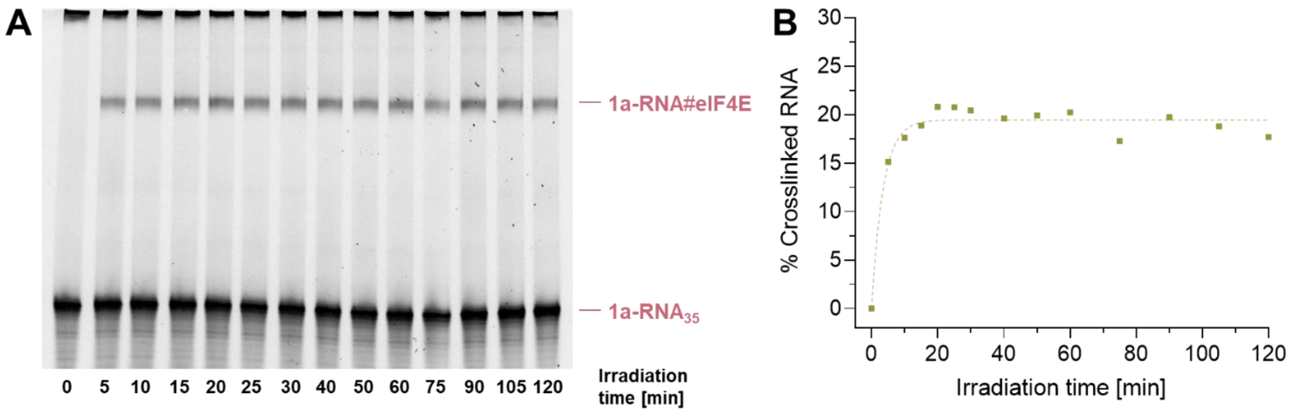

Figure S9. Optimization of irradiation time for 1a-RNA$_{35}$–eIF4E crosslinking. The intensities of the bands corresponding to free RNA and RNA-eIF4E crosslinks were estimated by densitometric analysis of the TBE PAGE gel (**A**) and plotted against irradiation time (**B**).

Compounds characterization

1+2: L2$_N$-m^{7}GpppA$_m$pG

Chemical structure

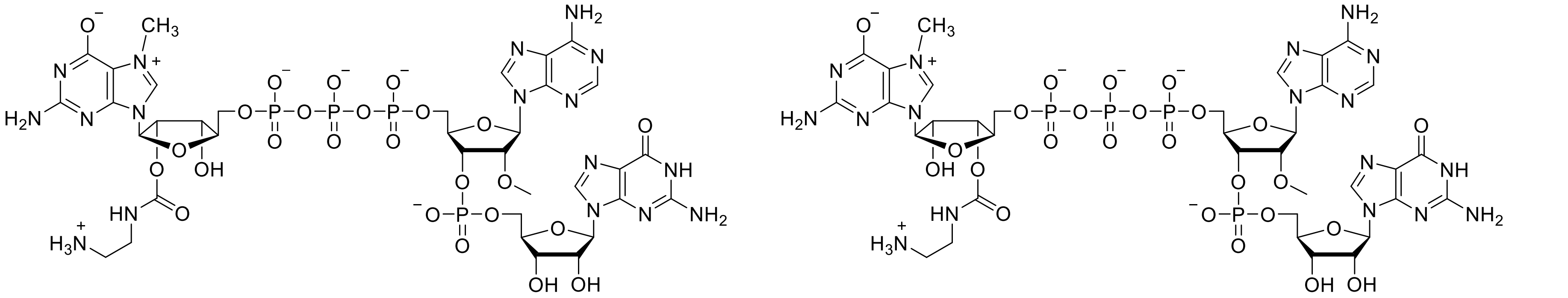

RP HPLC

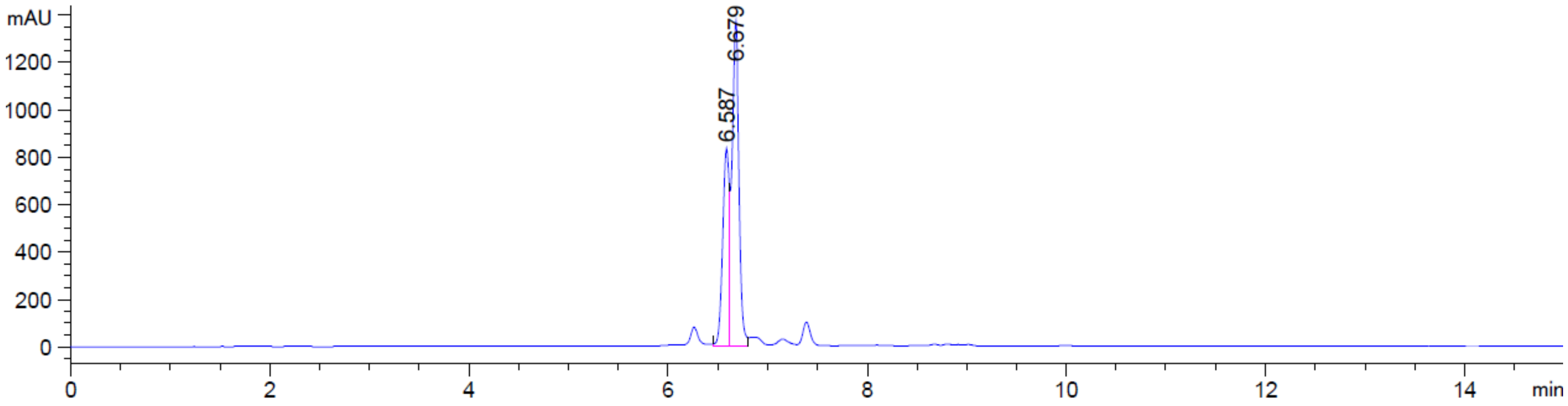

MS (-) ESI

(Calc. $[M-H]^-$ $C_{35}H_{48}N_{17}O_{25}P_4^-$ 1230.19632)

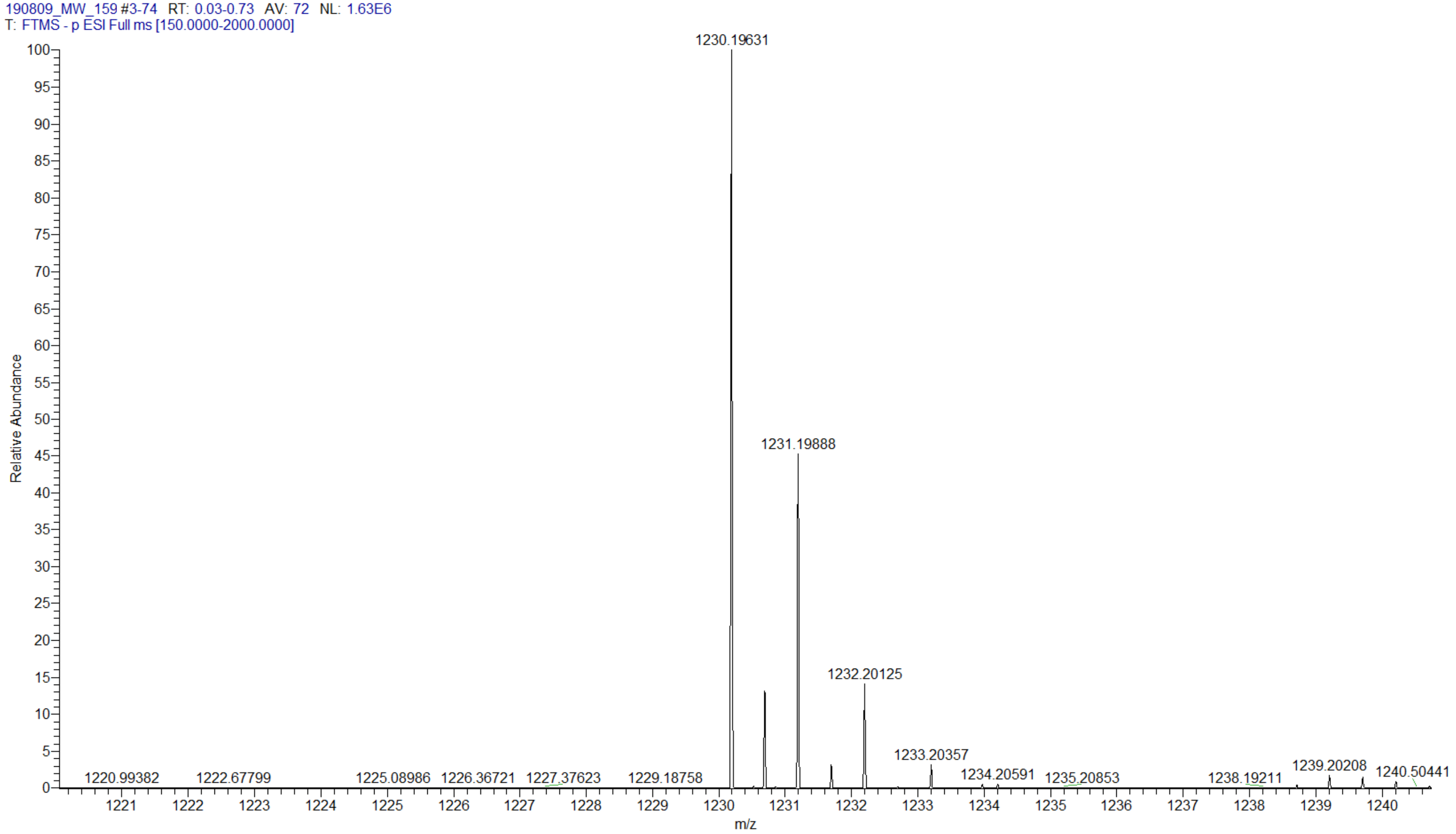

1a: Diaz-L2$_{N}$-m^{7}GpppA$_{m}$pG_I1	
**Chemical structure**	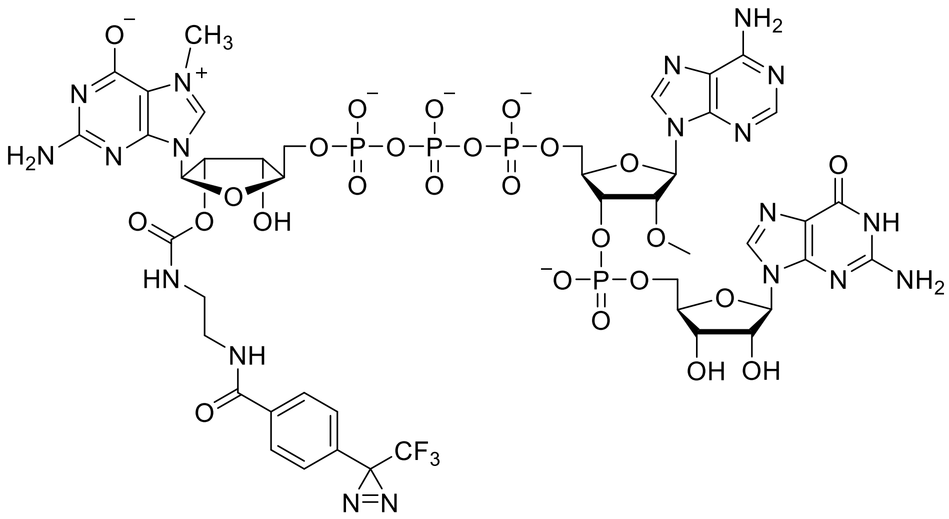
**RP HPLC**	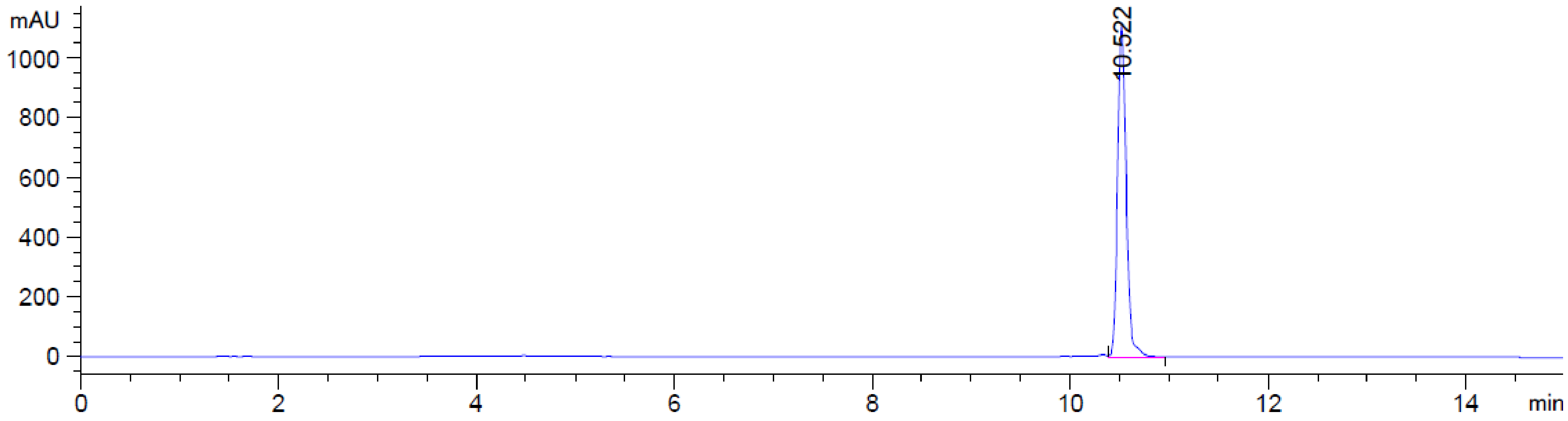

MS (-) ESI

(Calc. $[M-H]^-$ $C_{44}H_{51}F_3N_{19}O_{26}P_4^-$ 1442.21607)

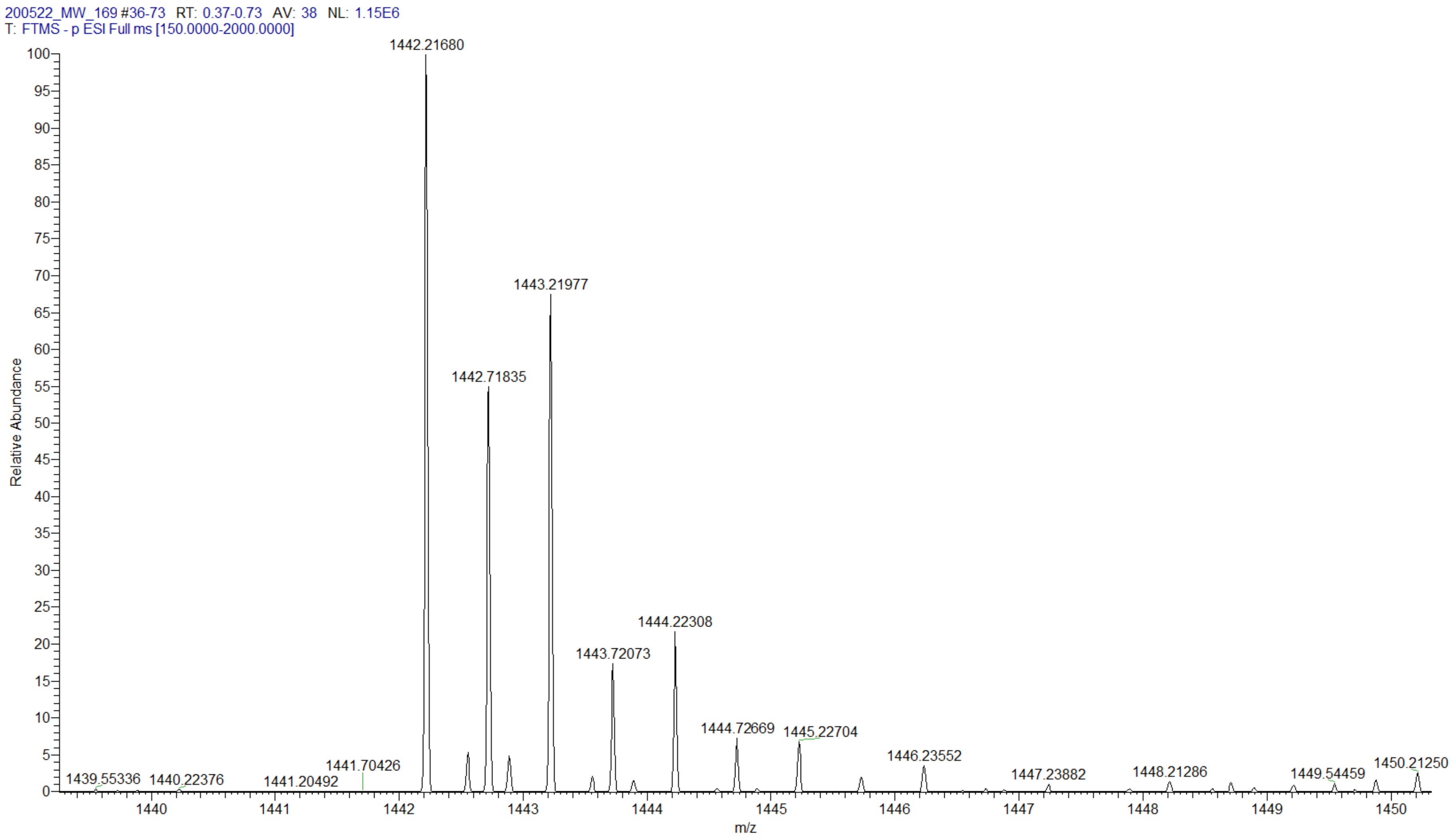

UV-Vis spectrum	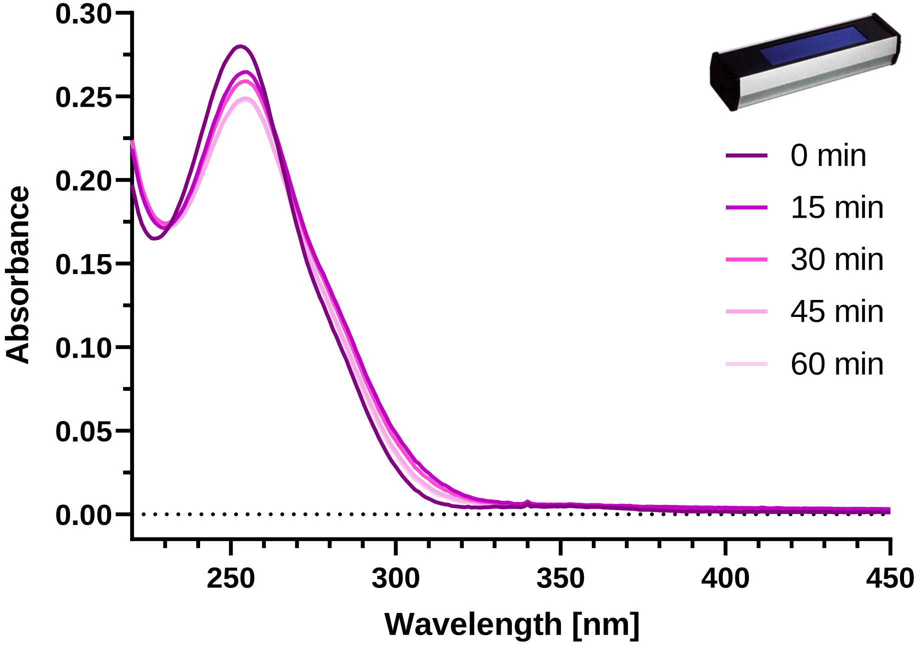

1a_UV: UV-irradiated Diaz-L2$_N$-m^{7}GpppA$_m$pG _I1	
**Chemical structure**	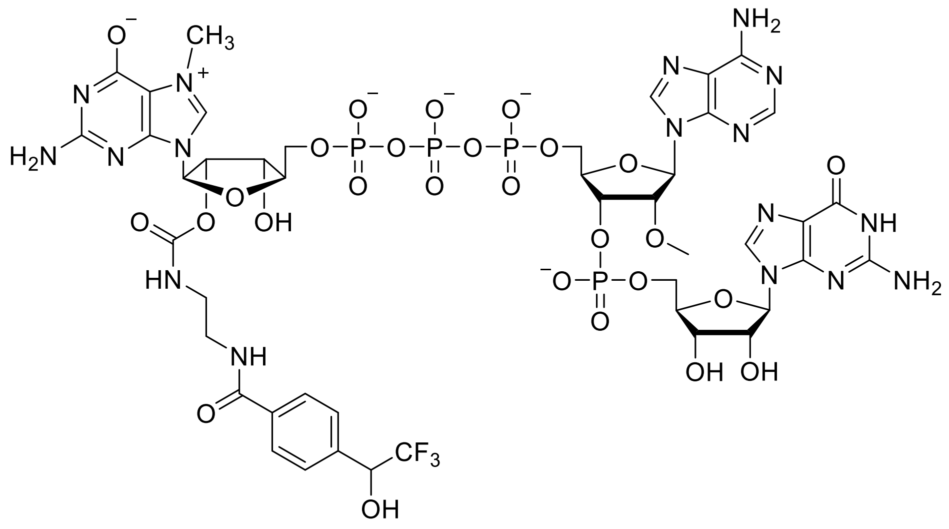
**RP HPLC**	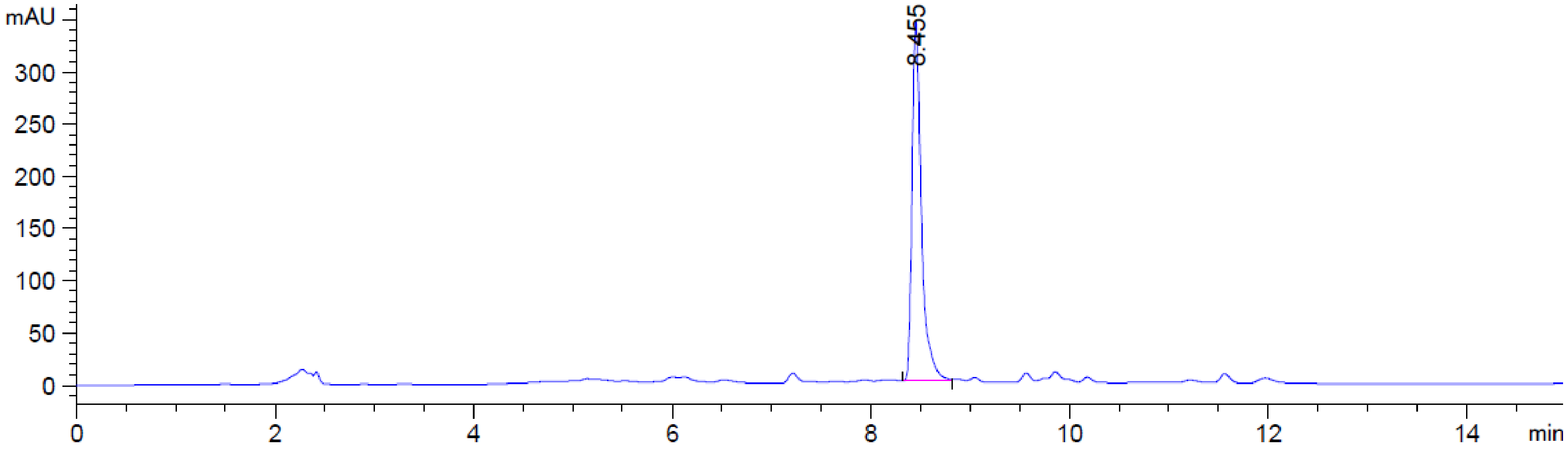

MS (-) ESI

(Calc. $[M-H]^-$ $C_{44}H_{53}F_3N_{17}O_{27}P_4^-$ 1432.22048)

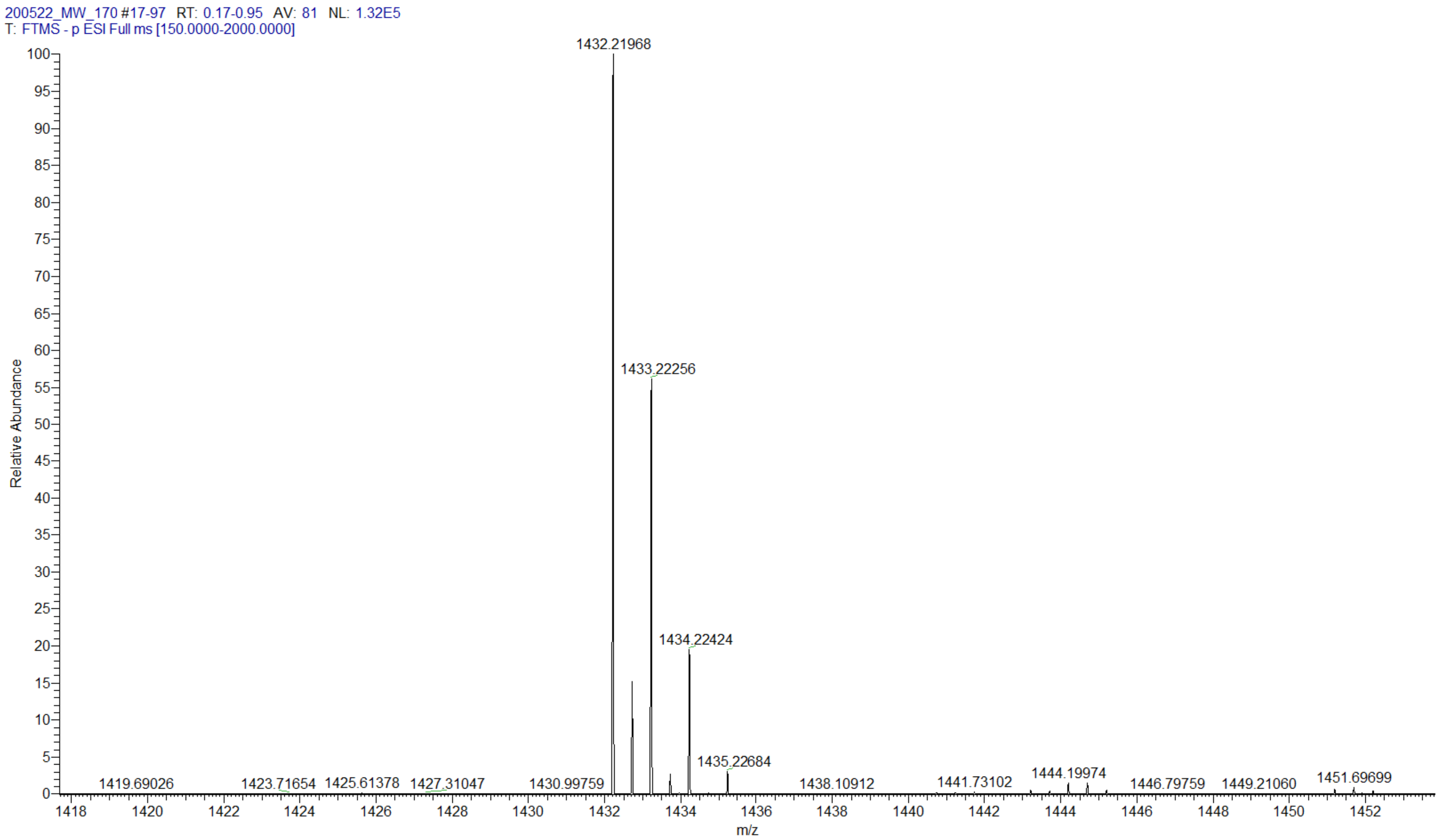

1b: NVA-L2$_N$-m^{7}GpppA$_m$pG_I1

**Chemical structure**	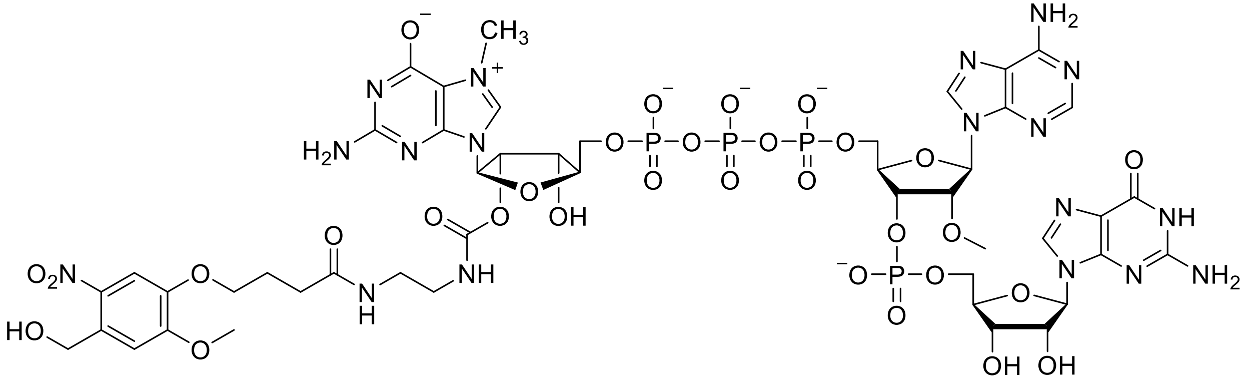
**RP HPLC**	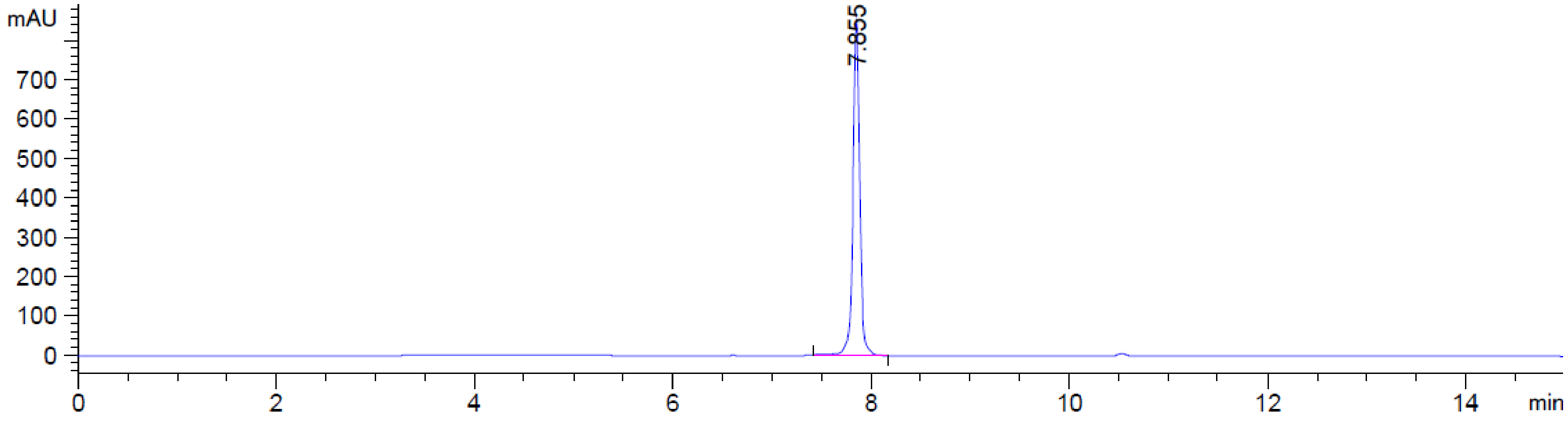

MS (-) ESI

(Calc. [M-H]$^-$ $C_{47}H_{61}N_{18}O_{31}P_4^-$ 1497.27061)

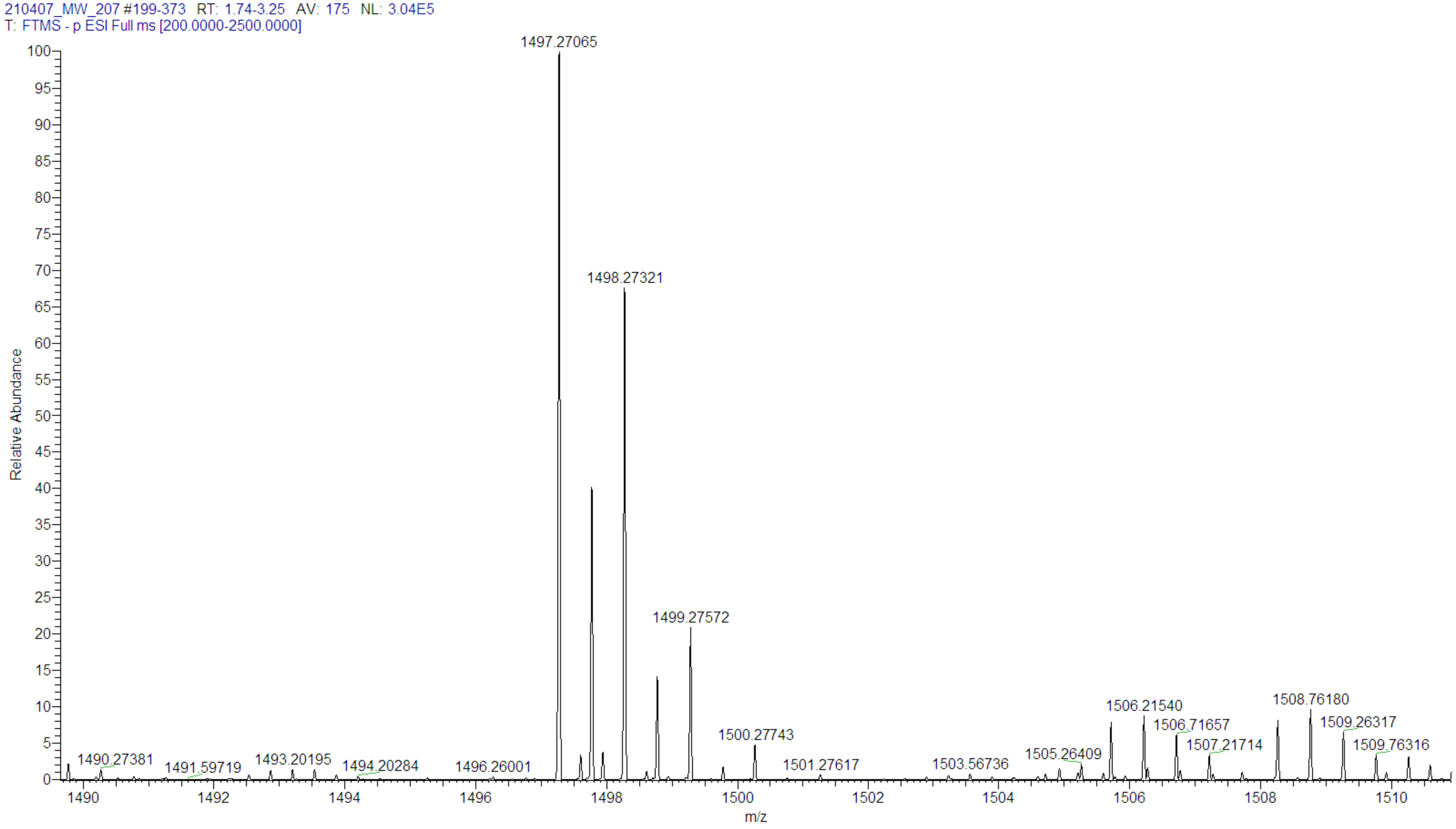

UV-Vis spectrum	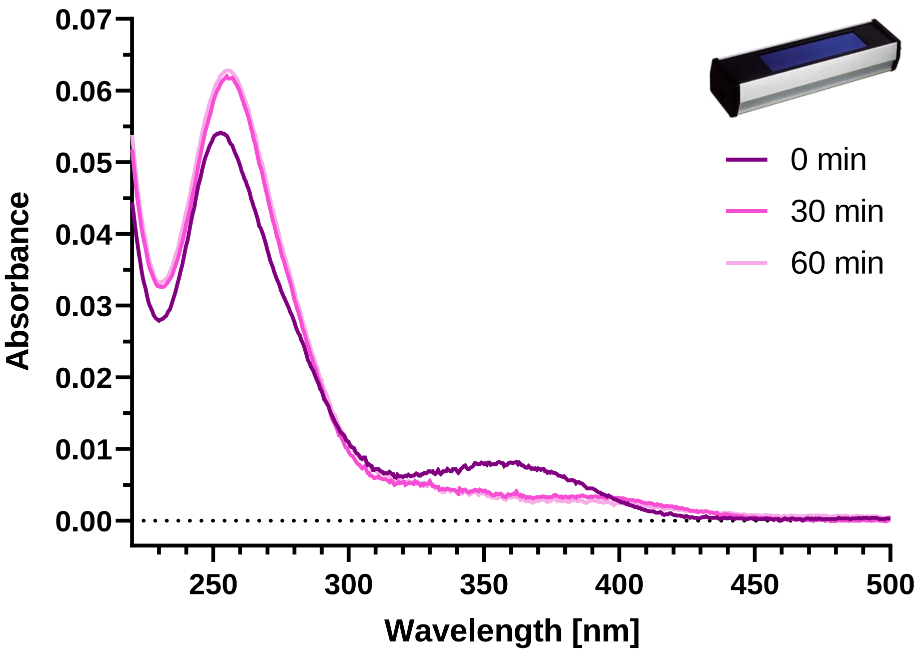

2a: Diaz-L2$_N$-m^{7}GpppA$_m$pG_I2

Chemical structure

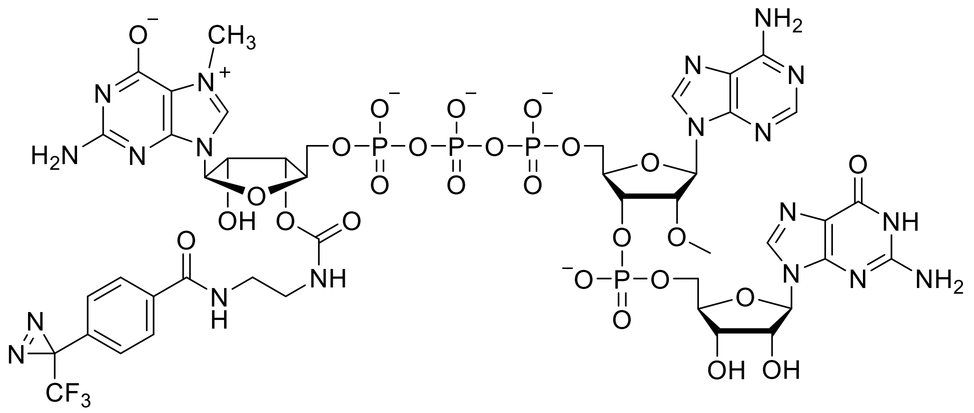

RP HPLC

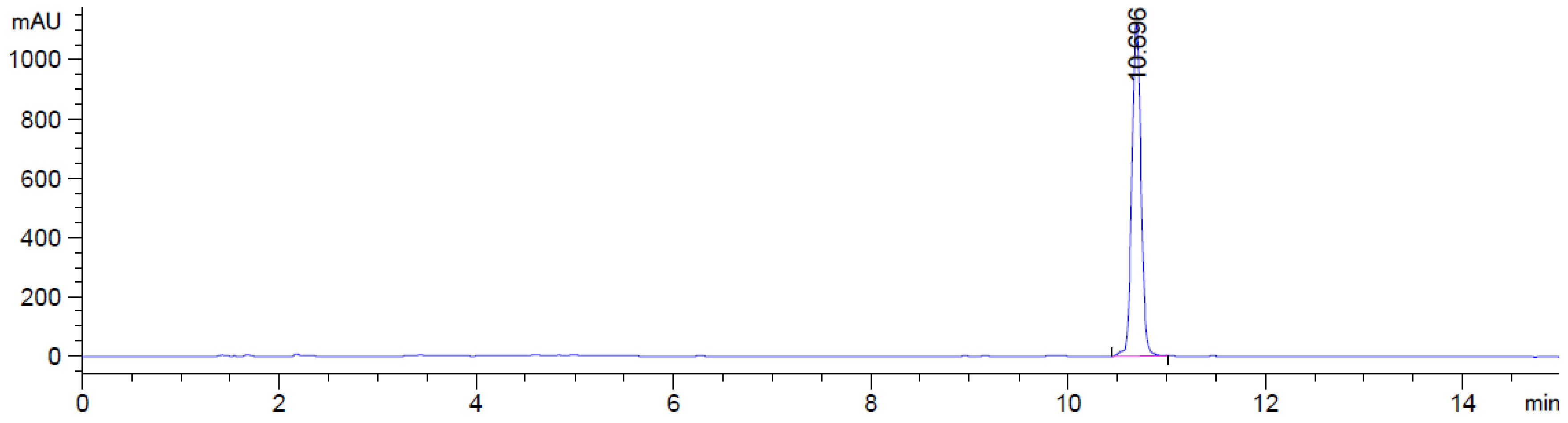

MS (-) ESI

(Calc. $[M\text{-}H]^-$ $C_{44}H_{51}F_3N_{19}O_{26}P_4^-$ 1442.21607)

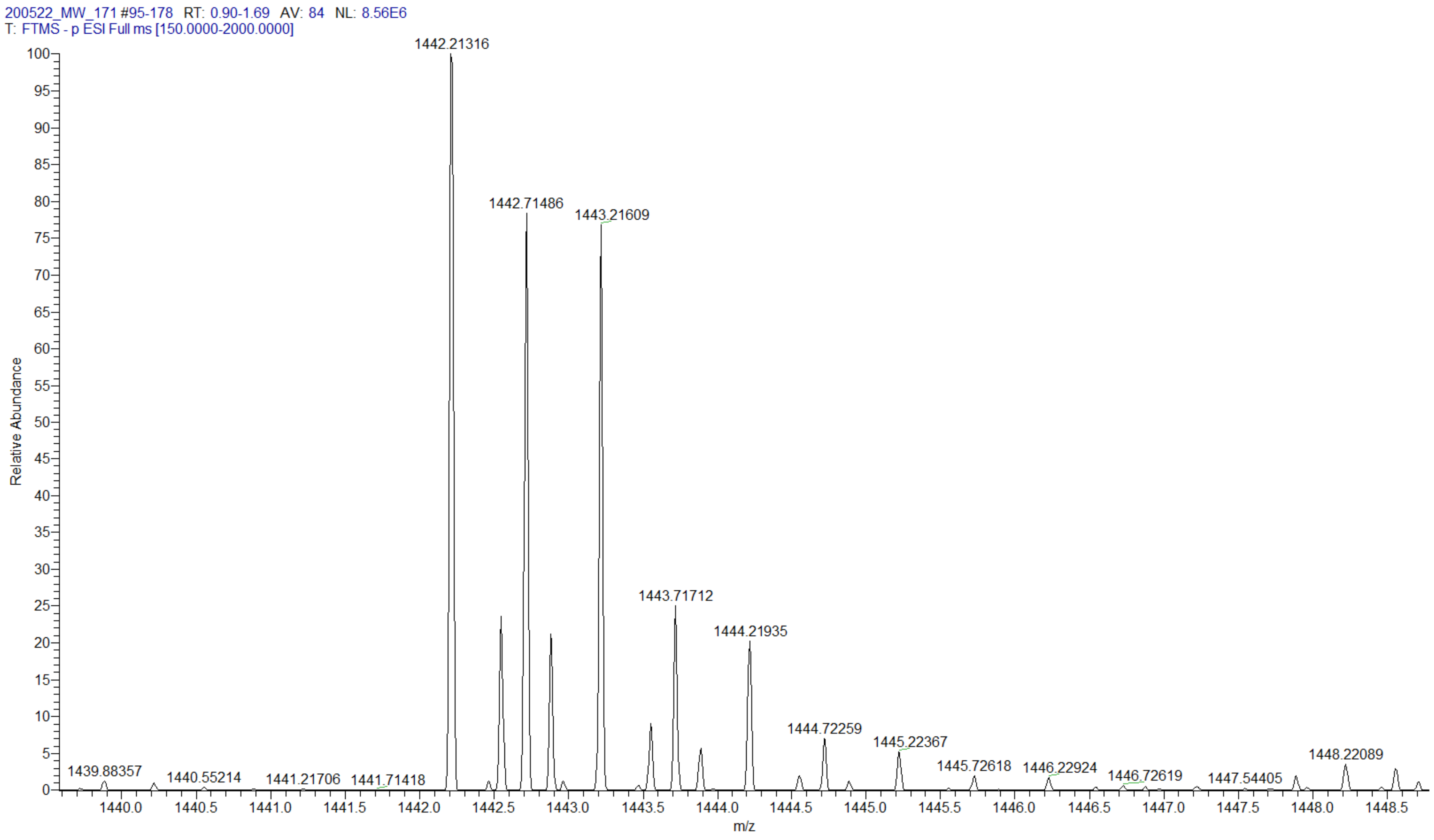

UV-Vis spectrum	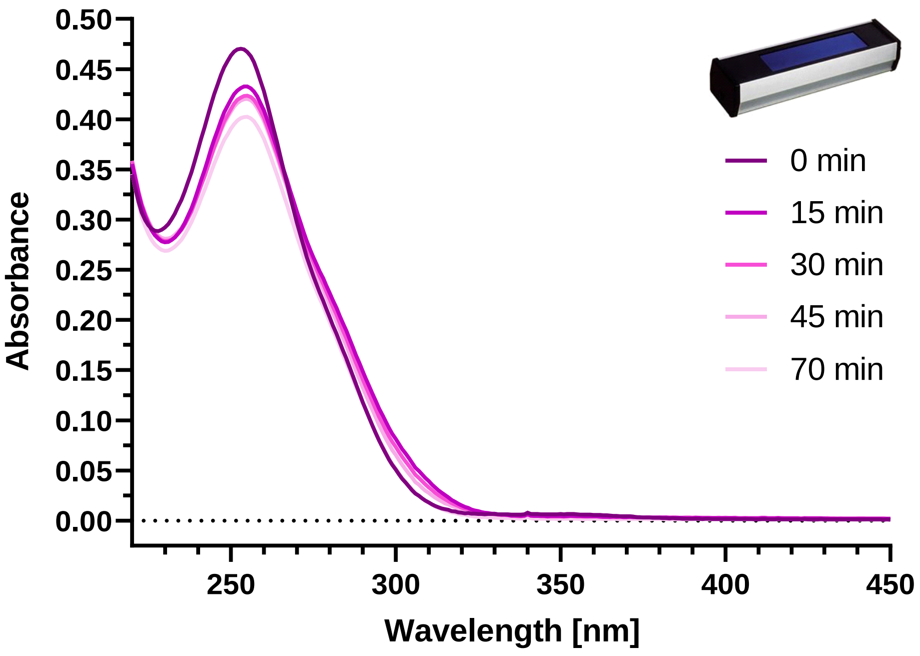

2a_UV: UV-irradiated Diaz-L2$_{N}$-m^{7}GpppA$_{m}$pG _I2	
**Chemical structure**	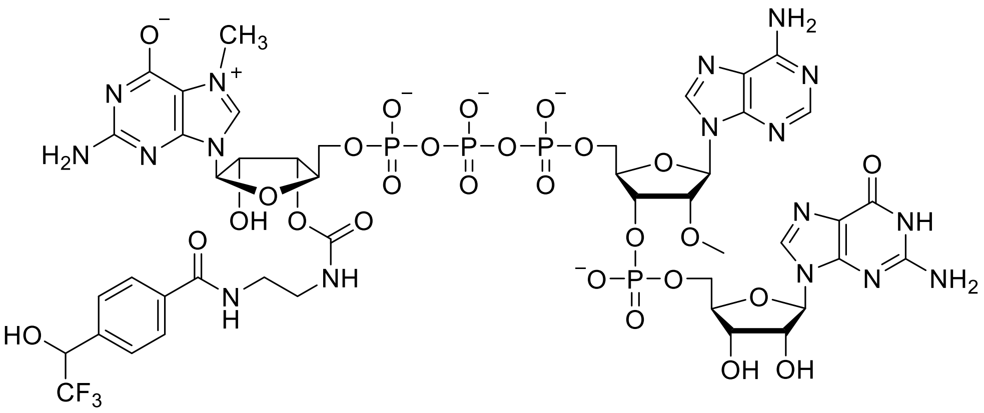
**RP HPLC**	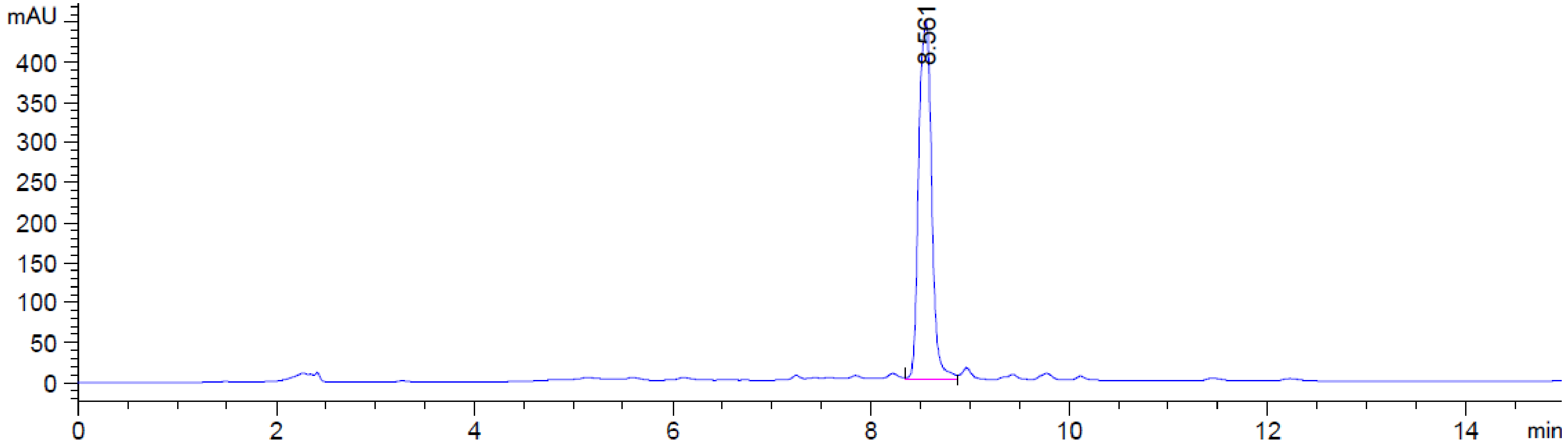

MS (-) ESI

(Calc. [M-H]$^-$ $C_{44}H_{53}F_3N_{17}O_{27}P_4^-$ 1432.22048)

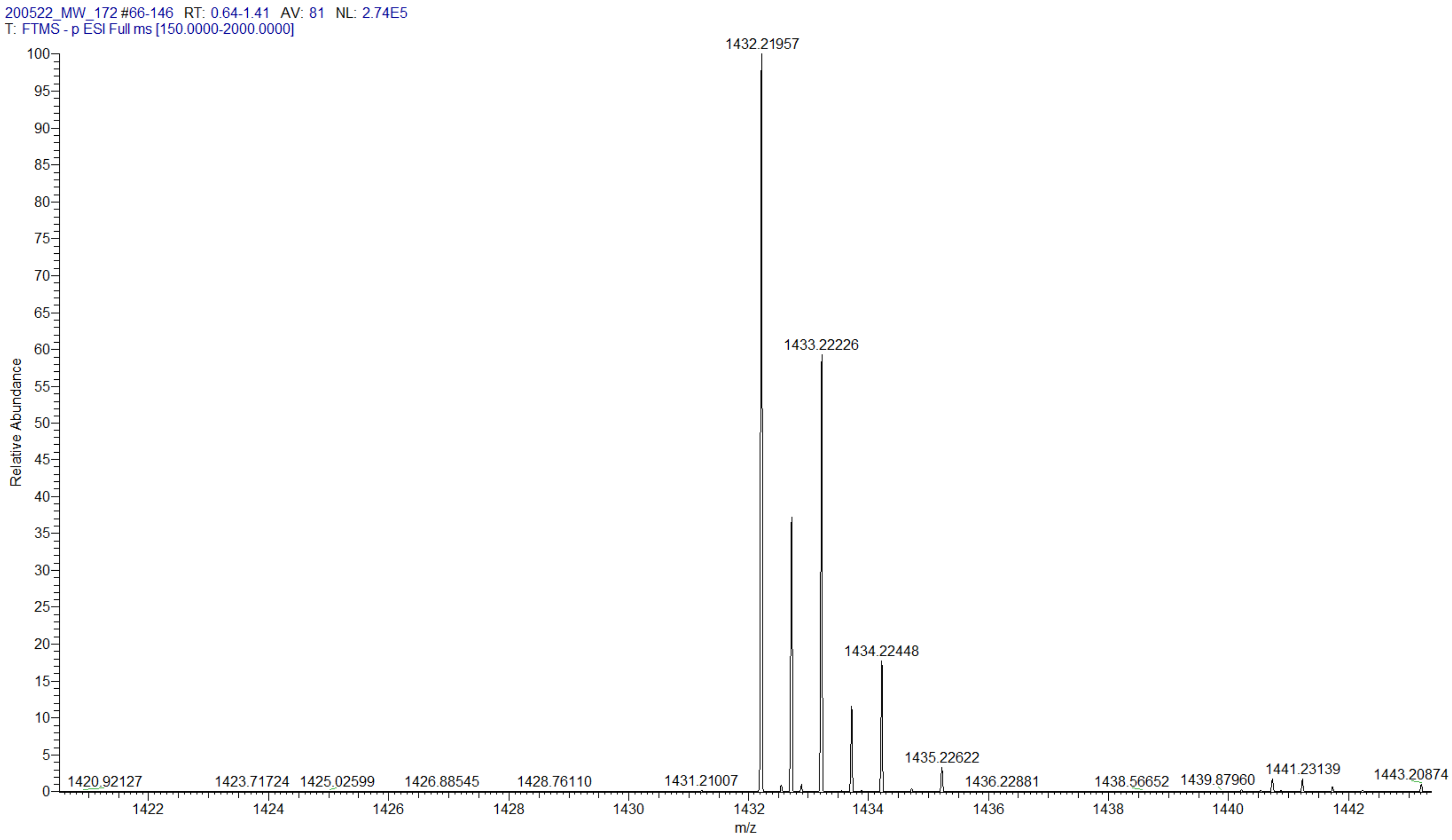

2b: NVA-L2$_{N}$-m^{7}GpppA$_{m}$pG_I2

Chemical structure

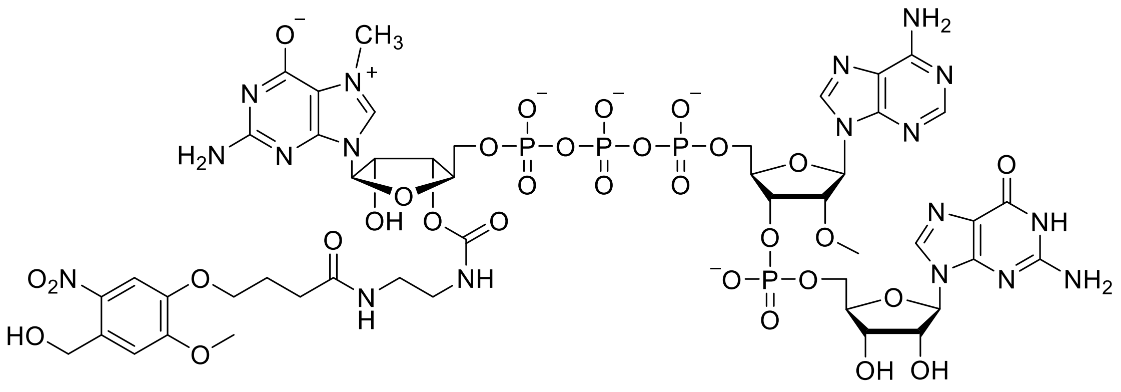

RP HPLC

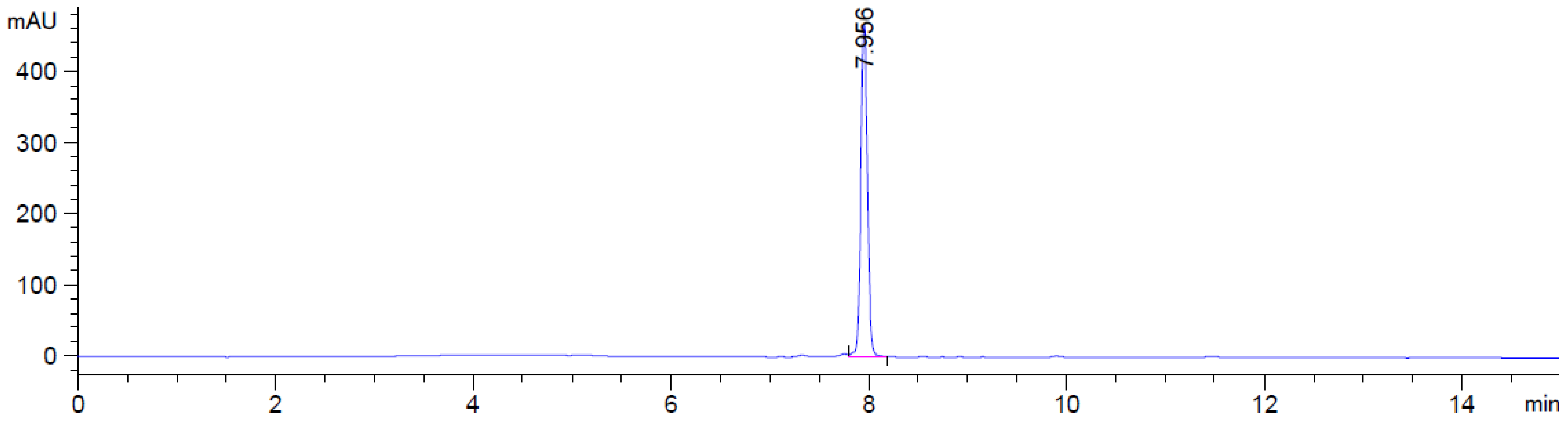

MS (-) ESI

(Calc. [M-H]$^-$ $C_{47}H_{61}N_{18}O_{31}P_4^-$ 1497.27061)

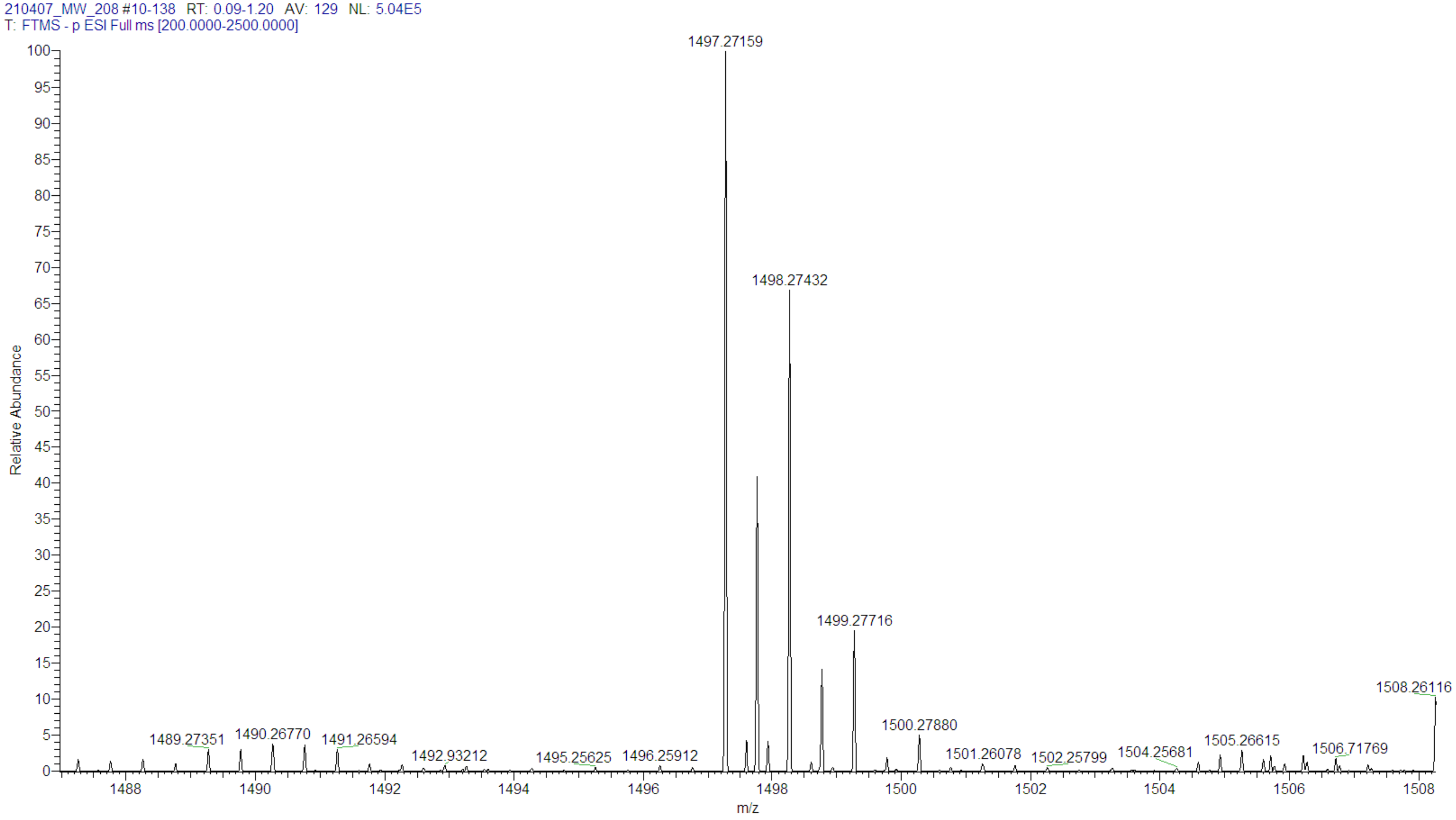

UV-Vis spectrum	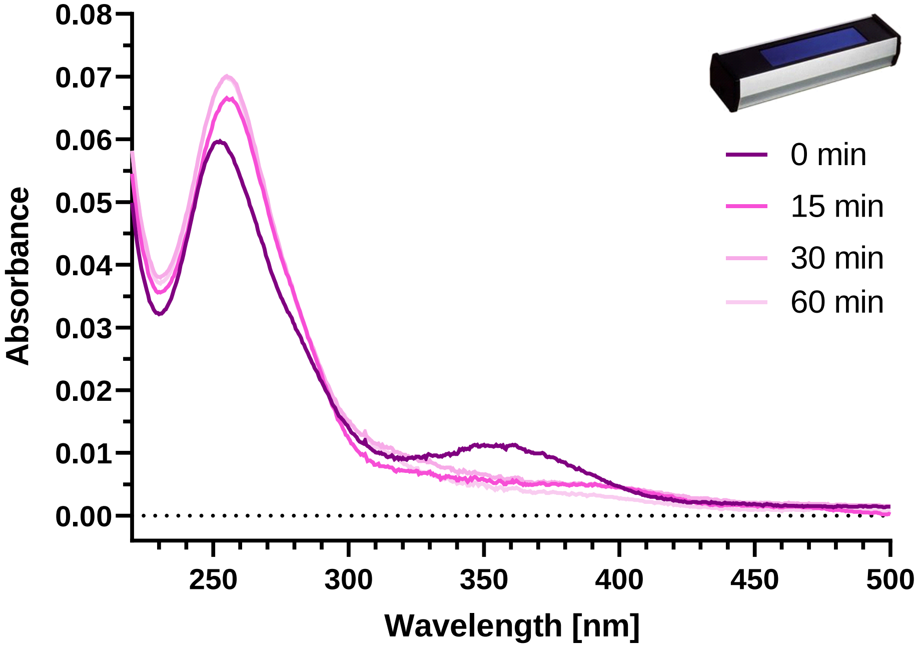

3: m^{7}Gppp^{L3N}A$_{m}$pG	
**Chemical structure**	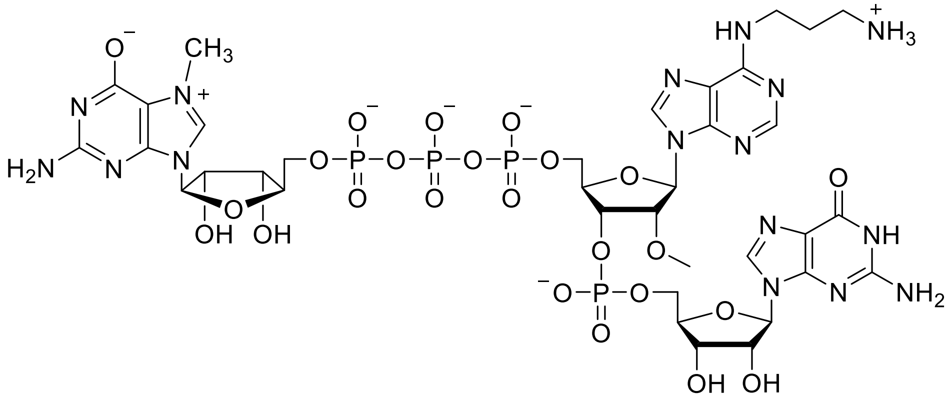
**RP HPLC**	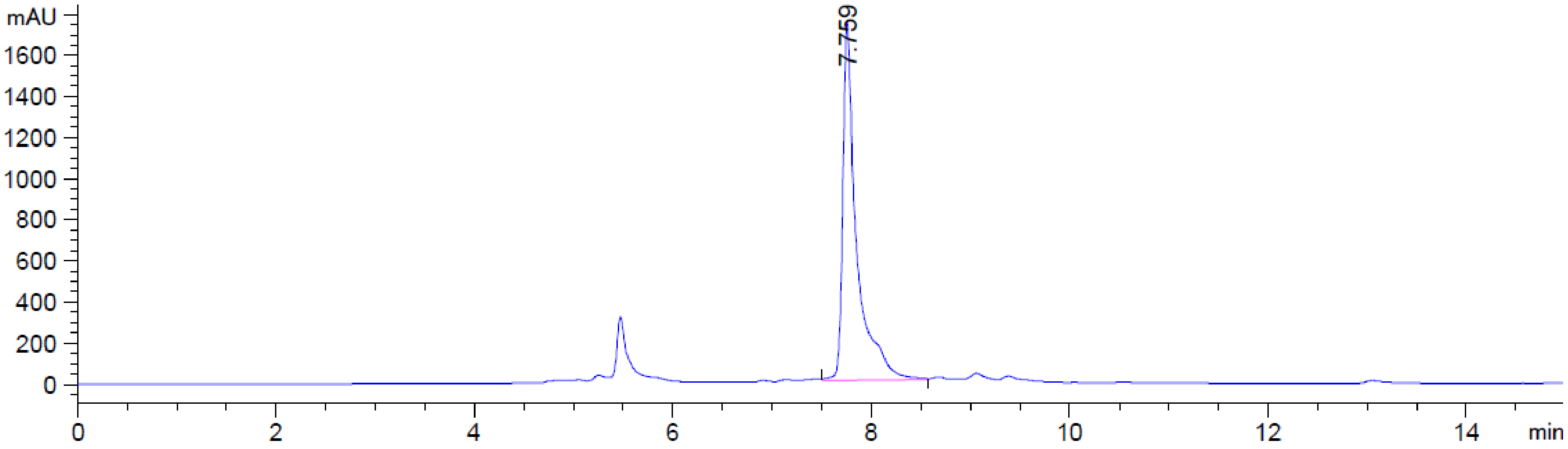

MS (-) ESI

(Calc. [M-H]$^-$ $C_{35}H_{49}F_3N_{16}O_{24}P_4^-$ 1201.20616)

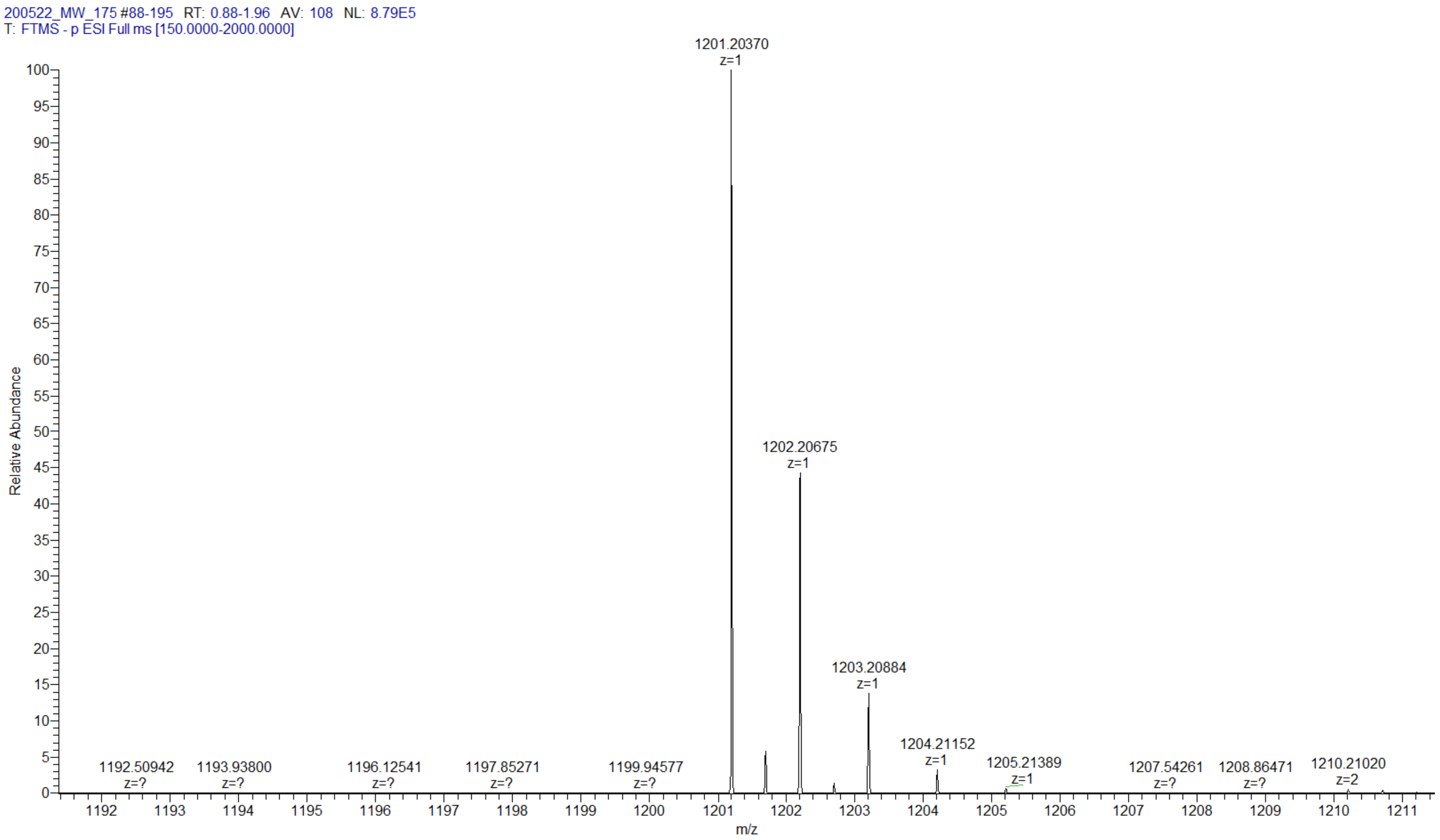

**3a: m^{7}Gppp$^{\text{Diaz-L3N}}$A$_{m}$pG**	
**Chemical structure**	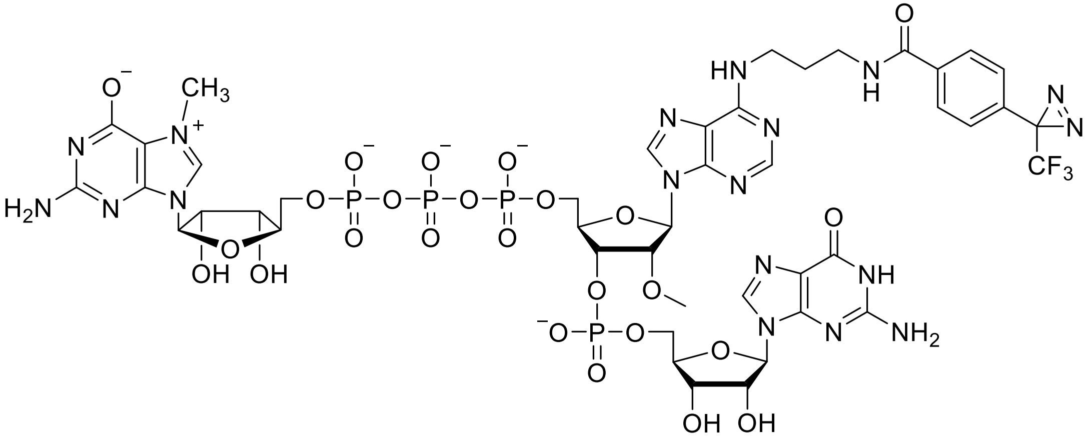
**RP HPLC**	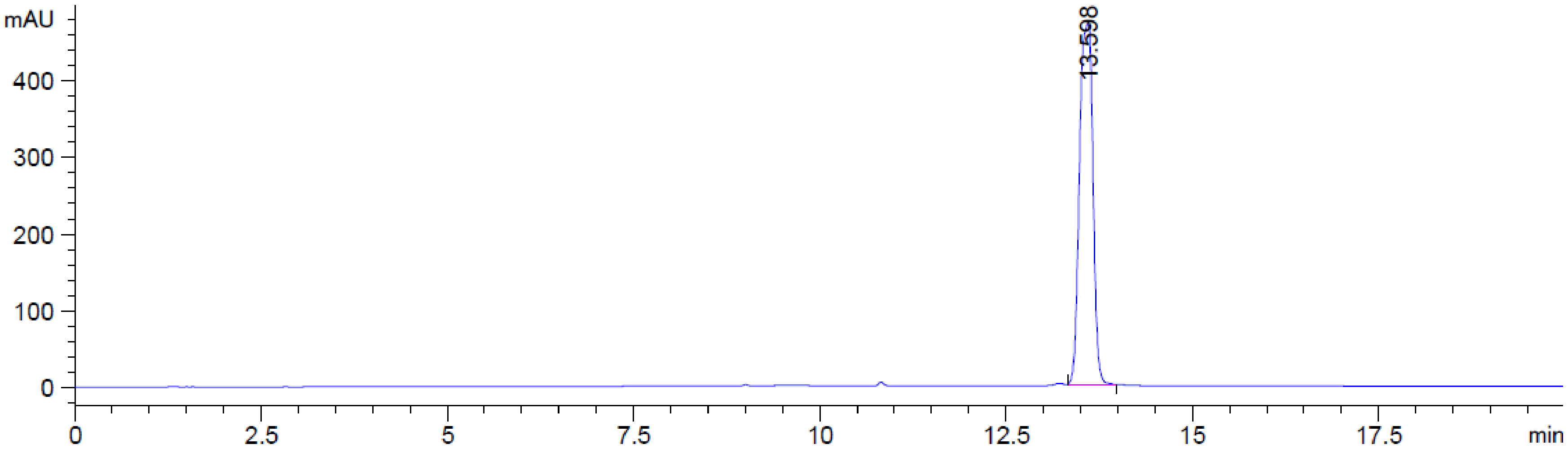

MS (-) ESI

(Calc. $[M-2H]^{2-}$ $C_{44}H_{51}F_{3}N_{18}O_{25}P_{4}^{2-}$ 706.10931)

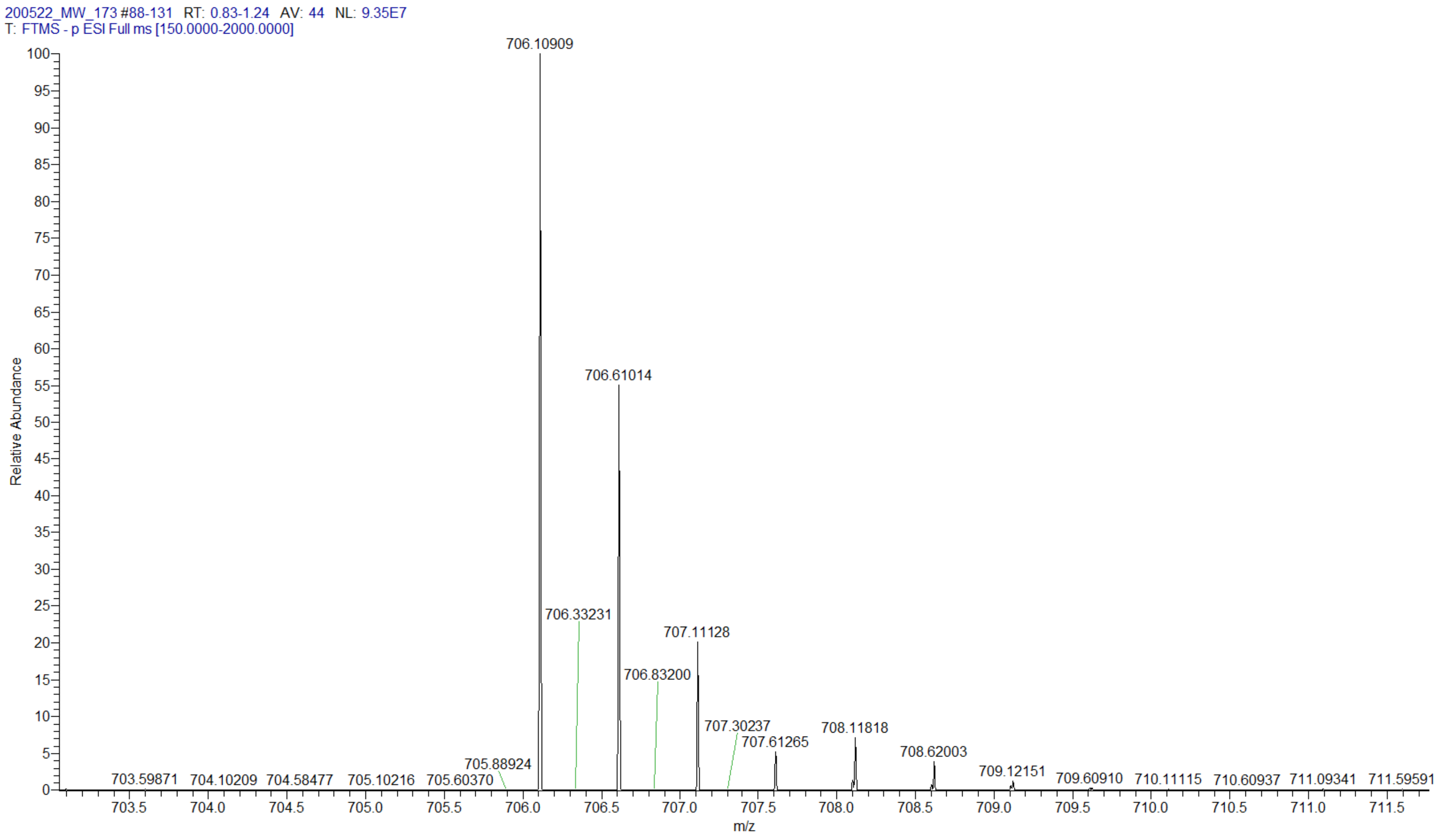

**UV-Vis spectrum**	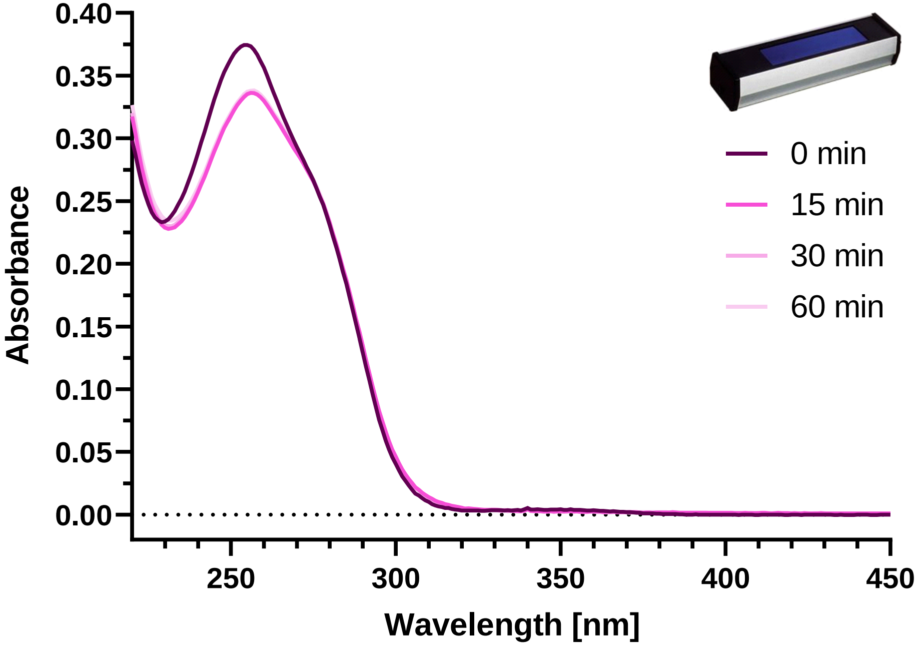

<table>
<tr><th colspan="2">3a_UV: UV-irradiated m^{7}Gppp$^{Diaz\text{-}L3N}$A$_m$pG</th></tr>
<tr><td>Chemical structure</td><td>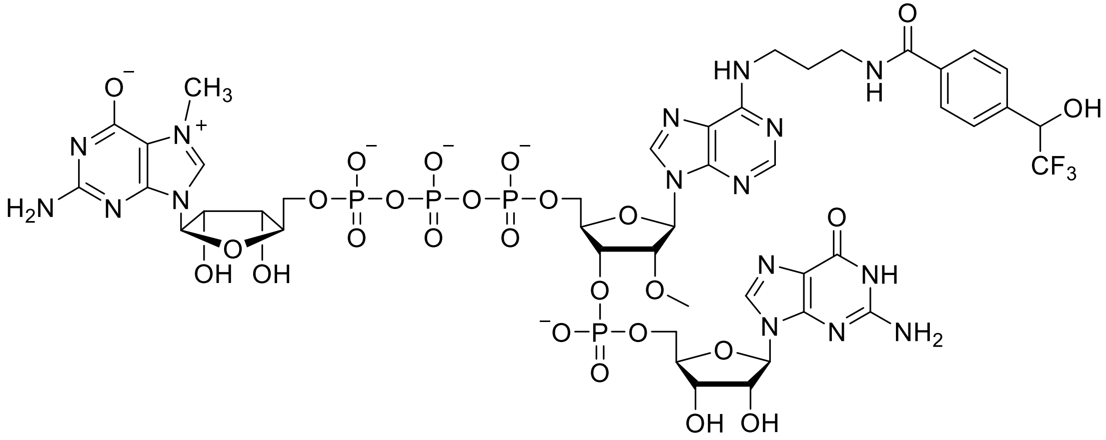
</td></tr>
<tr><td>RP HPLC</td><td>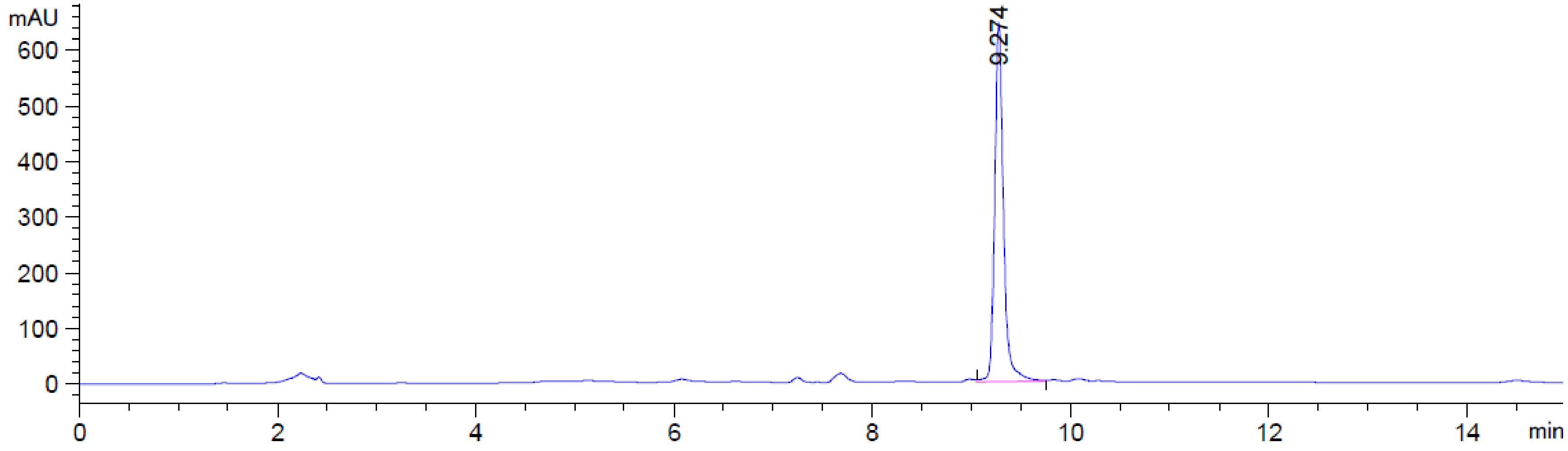
</td></tr>
</table>

MS (-) ESI

(Calc. $[M-2H]^{2-}$ $C_{44}H_{53}F_3N_{16}O_{26}P_4^{\,2-}$ 701.11152)

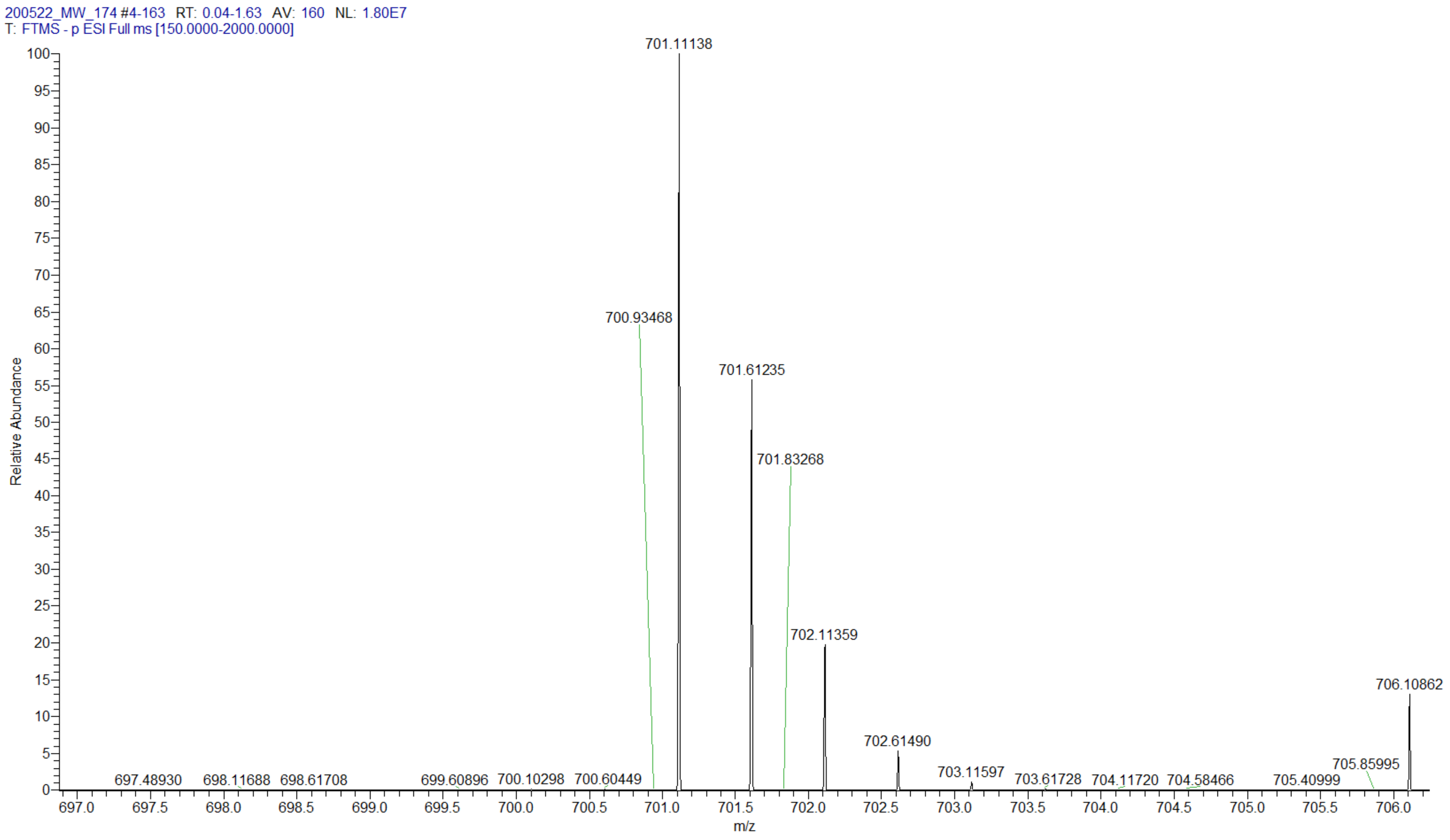

3b: m^{7}Gppp$^{NVA\text{-}L3N}$A$_{m}$pG

Chemical structure

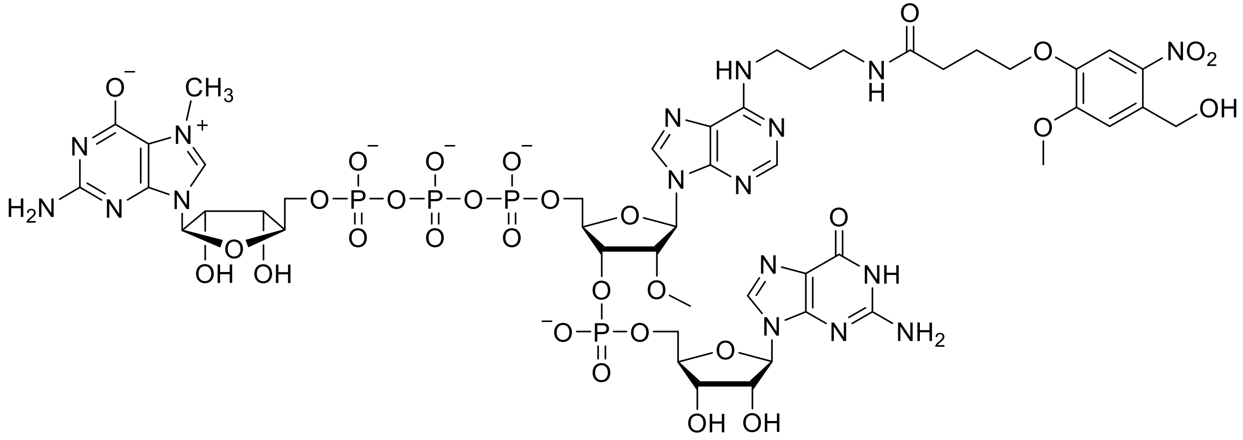

RP HPLC

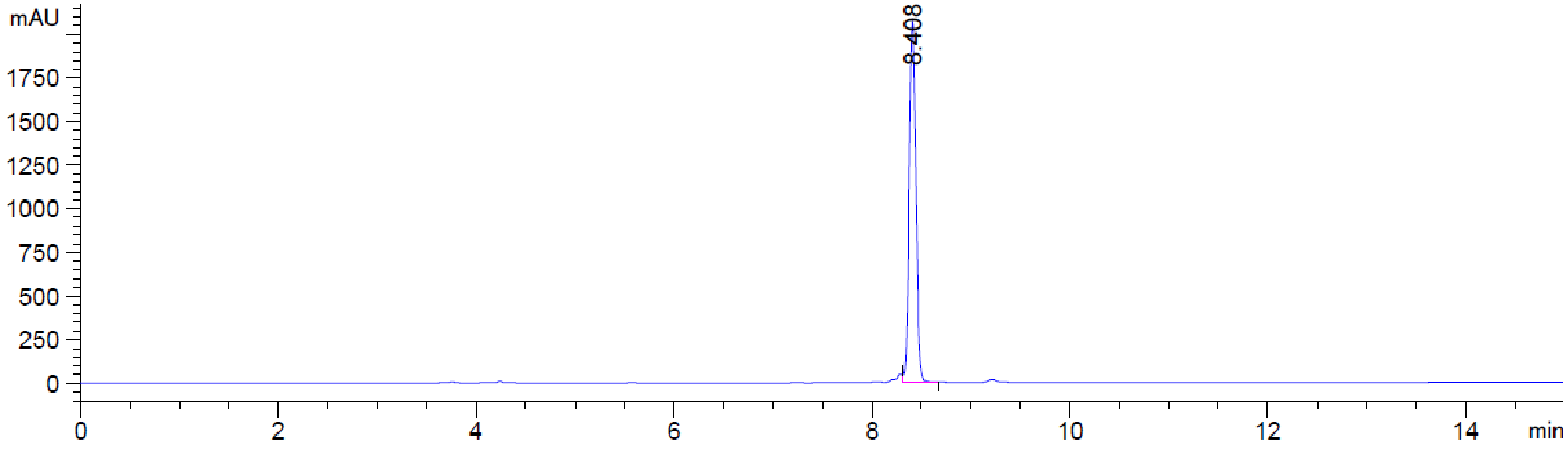

MS (-) ESI

(Calc. $[M-H]^-$ $C_{46}H_{60}N_{17}O_{30}P_4^-$ 1454.26479)

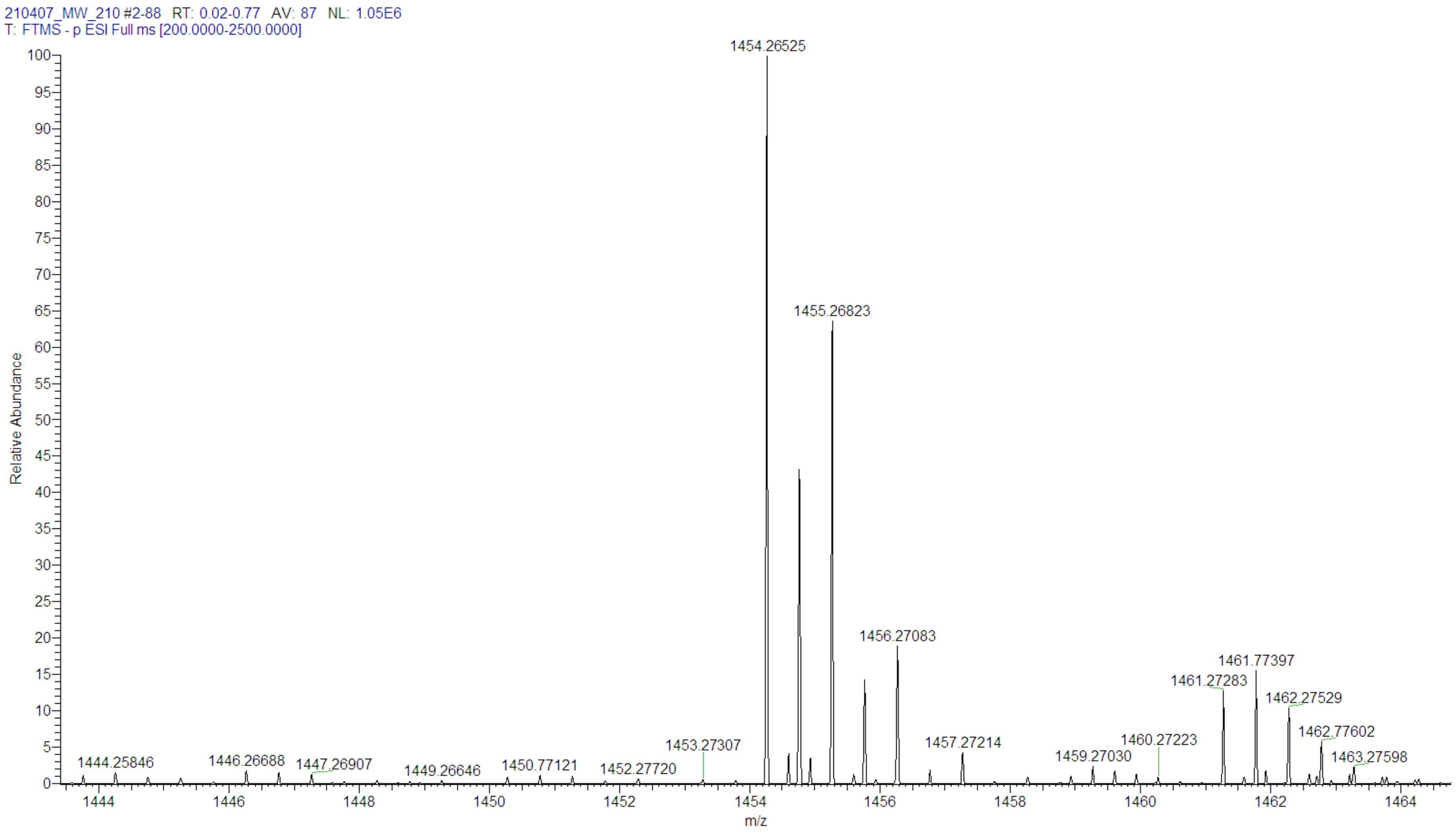

UV-Vis spectrum	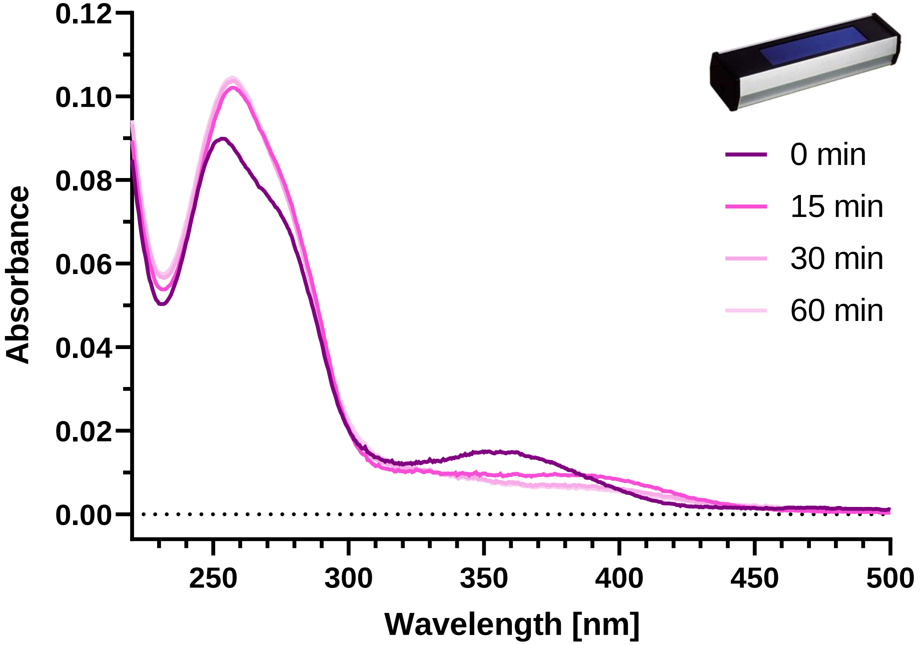

4: m^{7}GpppA$_{L3N}$pG

Chemical structure

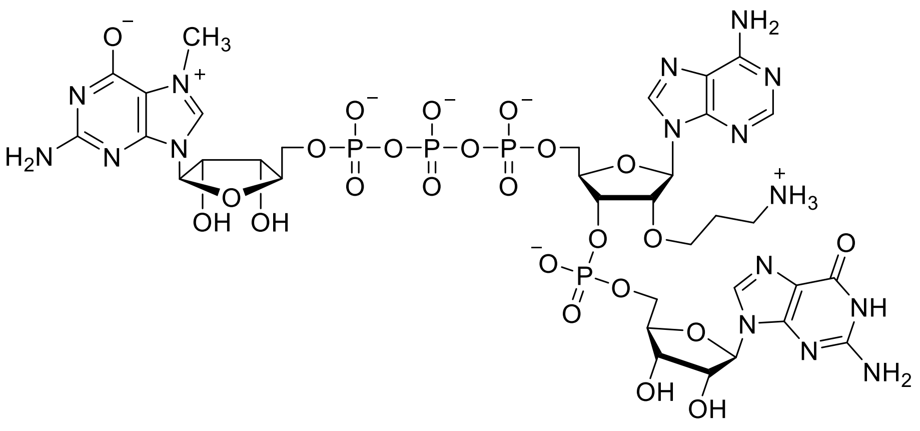

RP HPLC

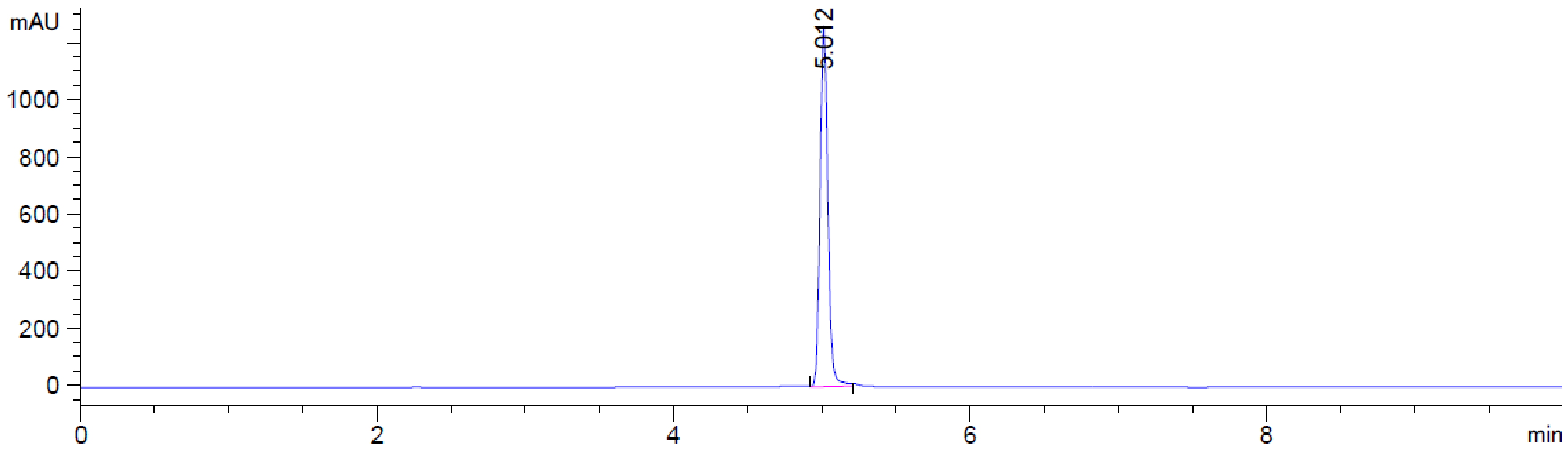

MS (-) ESI

(Calc. $[M-H]^-$ $C_{34}H_{47}N_{16}O_{24}P_4^-$ 1187.19051)

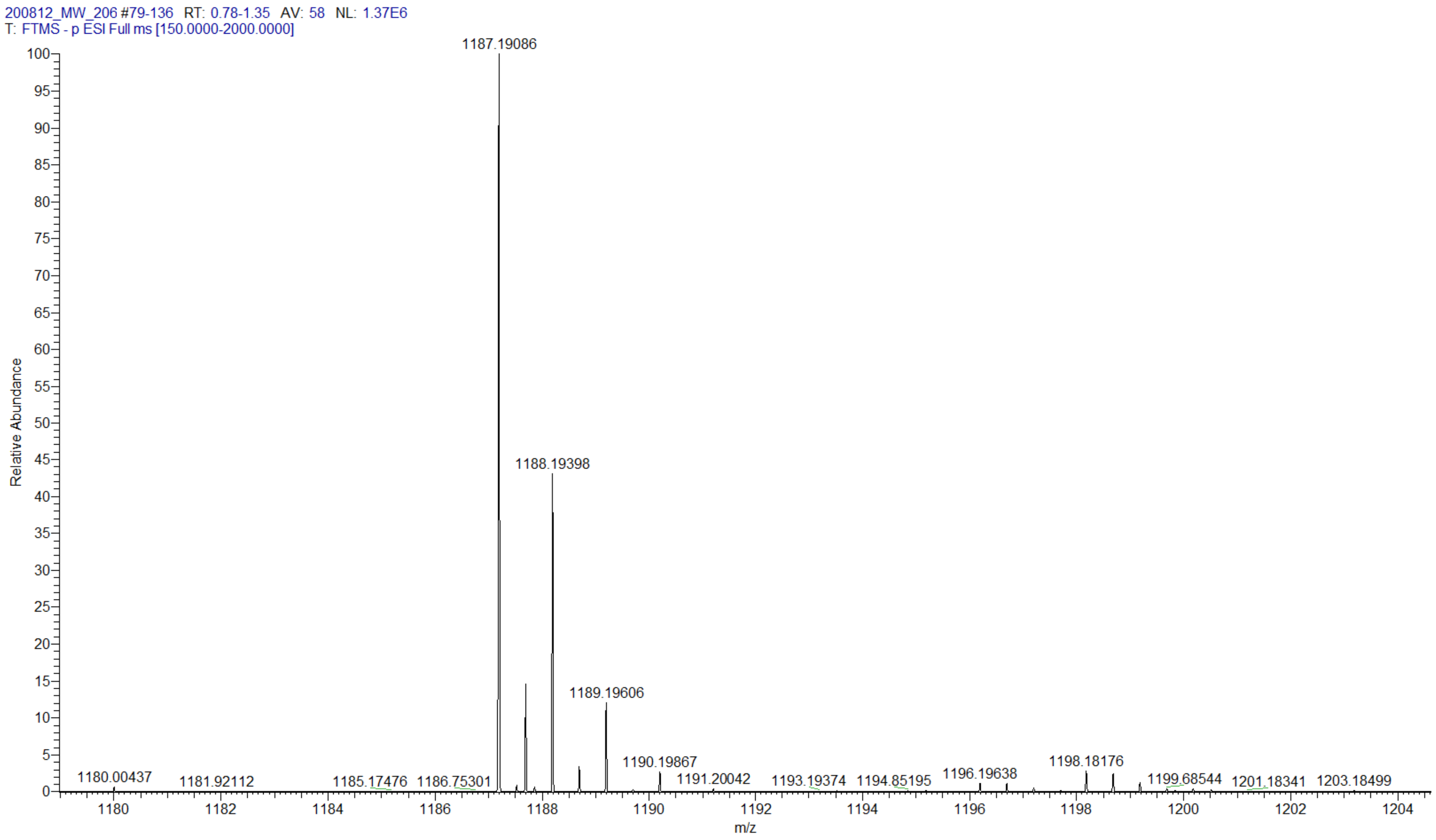

4a: m^{7}GpppA$_{L3N\text{-}Diaz}$pG

**Chemical structure**	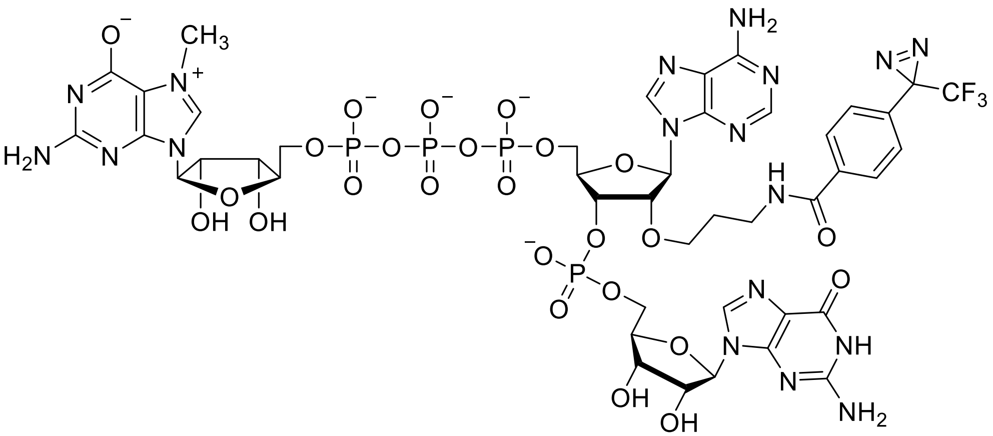
**RP HPLC**	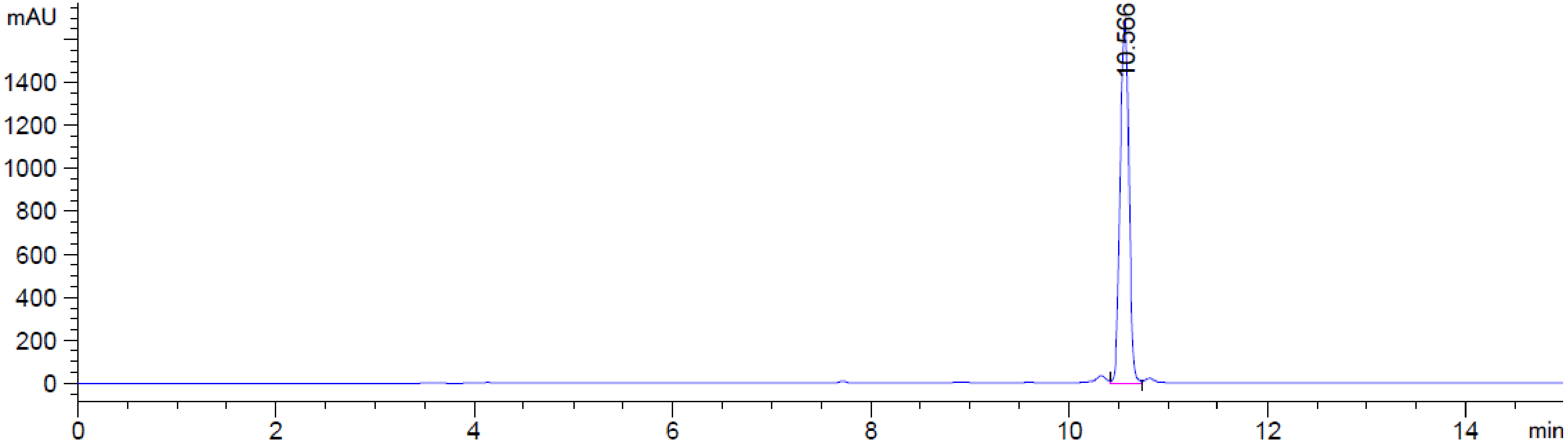

MS (-) ESI

(Calc. $[M-H]^-$ $C_{43}H_{50}F_3N_{18}O_{25}P_4^-$ 1399.21025)

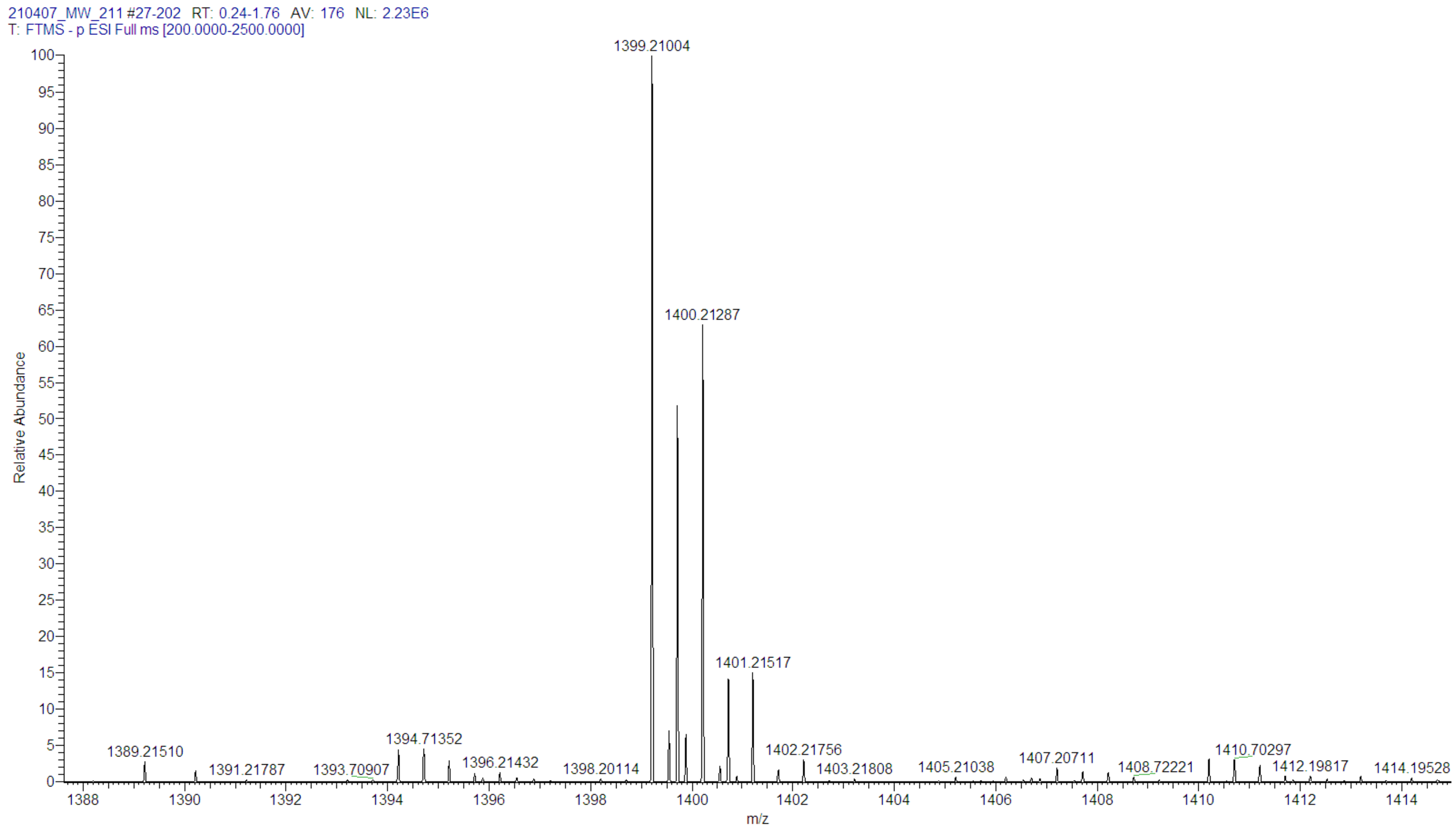

UV-Vis spectrum	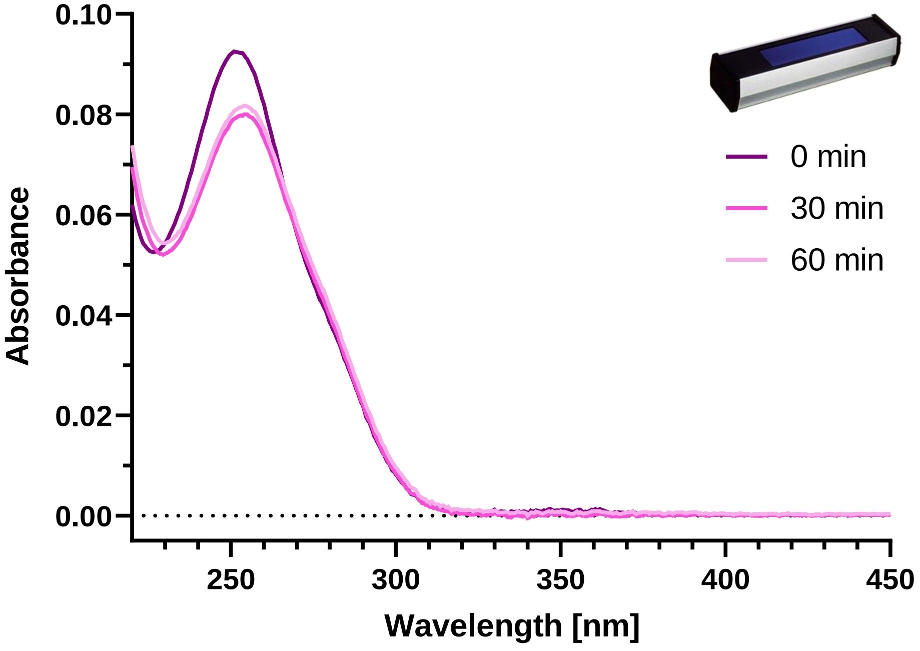

**4b: m⁷GpppA$_{L3N-NVA}$pG**	
**Chemical structure**	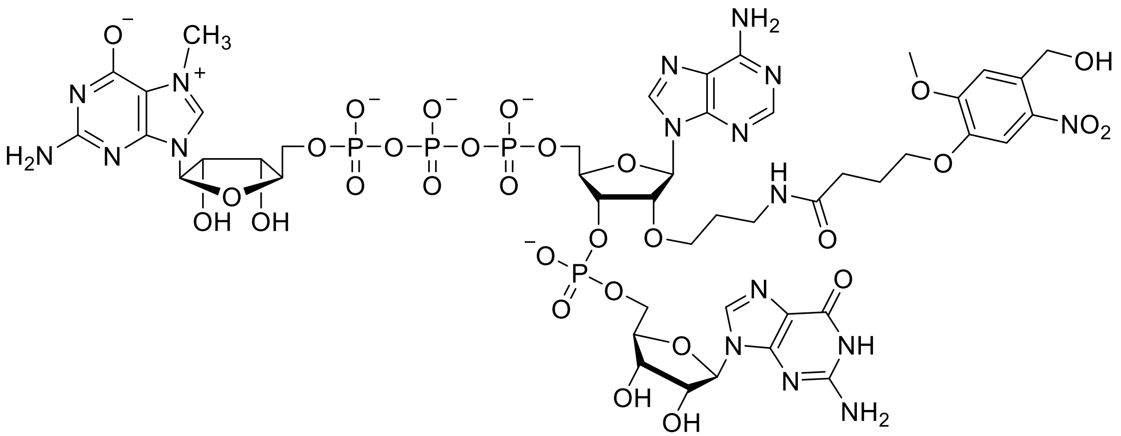
**RP HPLC**	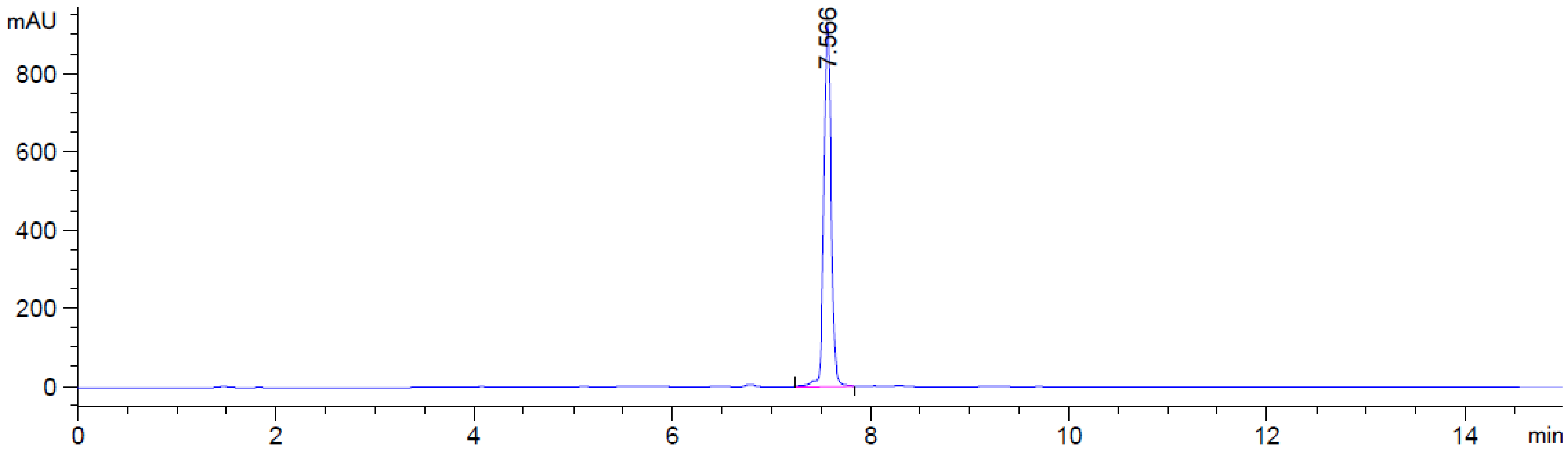

MS (-) ESI

(Calc. [M-H]$^-$ $C_{46}H_{60}N_{17}O_{30}P_4^-$ 1454.26479)

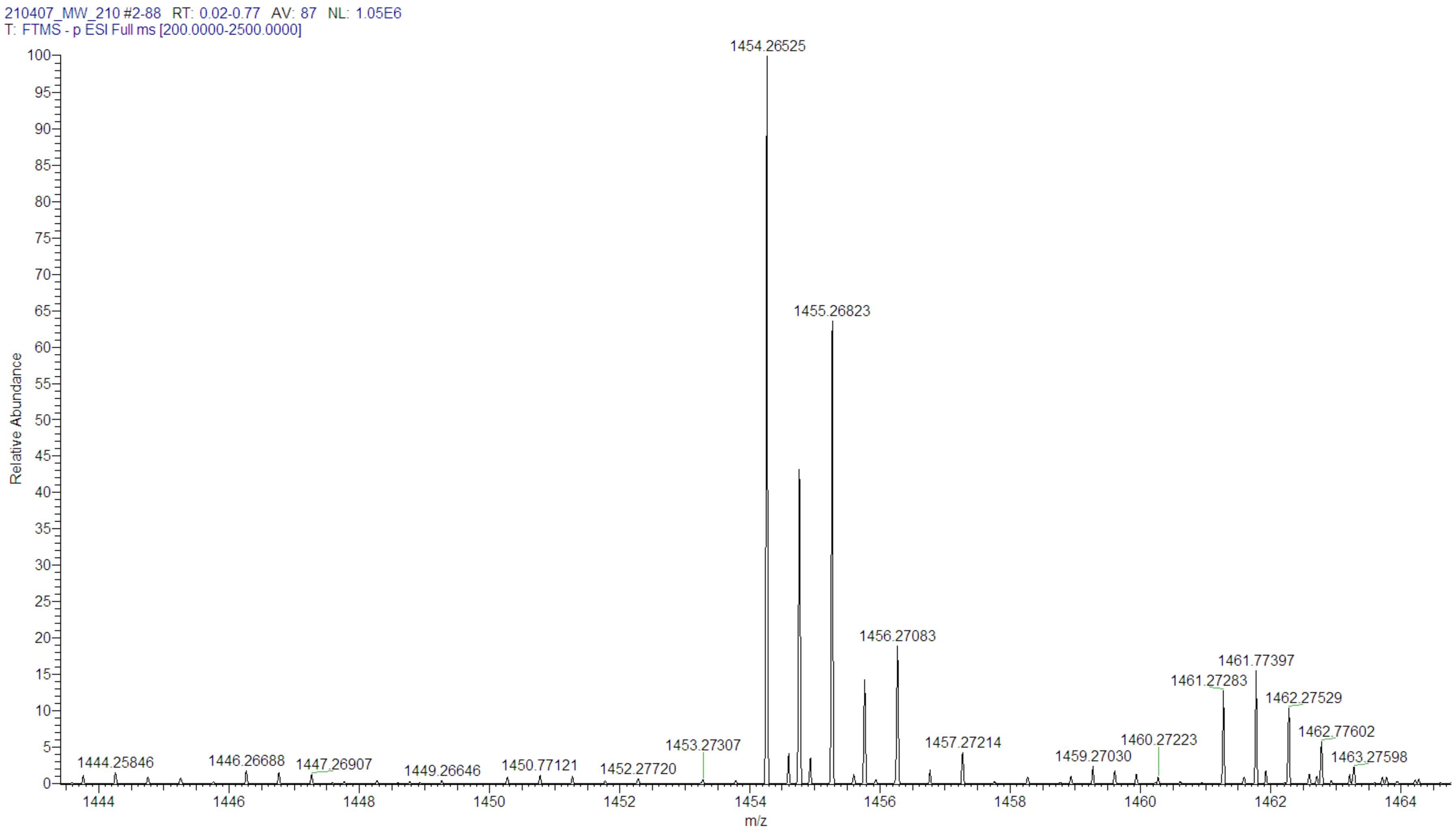

UV-Vis spectrum	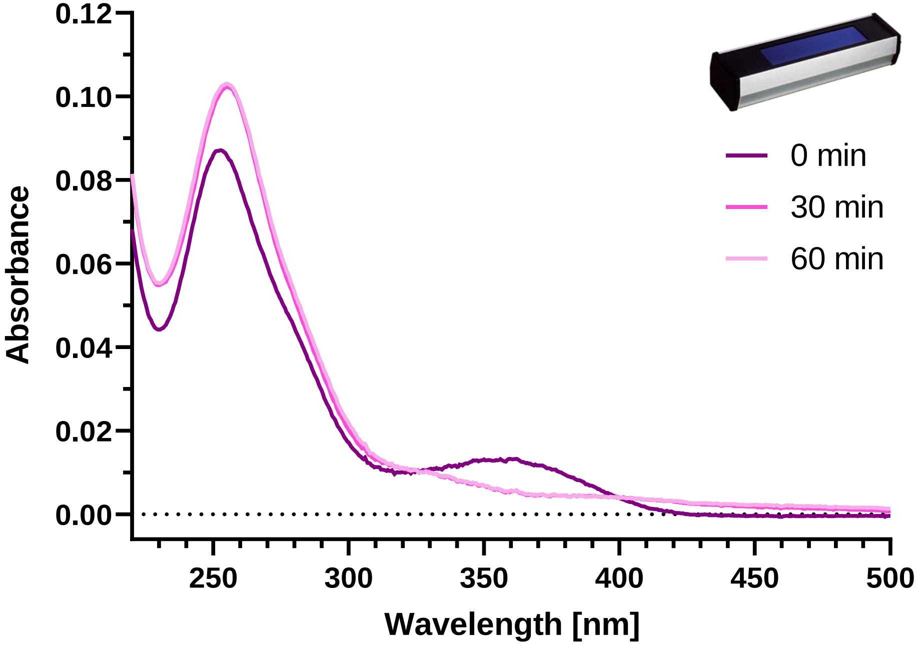

5: $L2_{N}$-GpppA_{m}pG	
**Chemical structure**	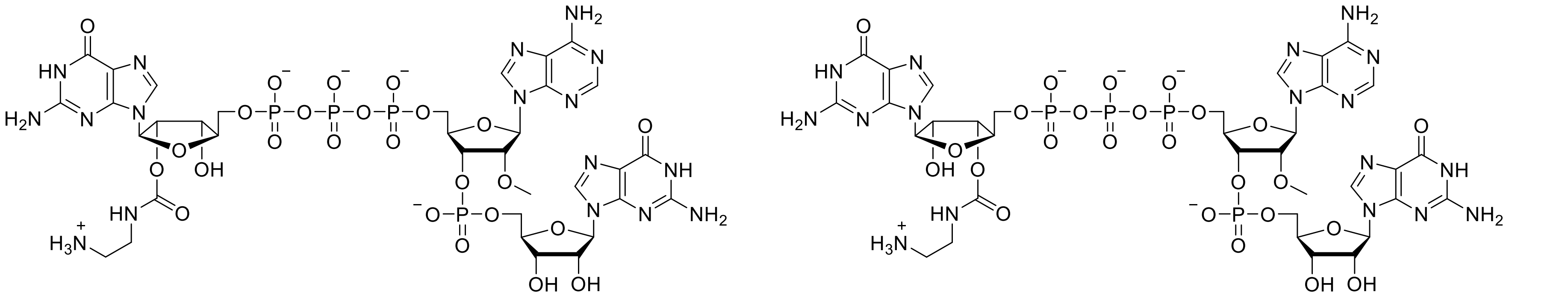
**RP HPLC**	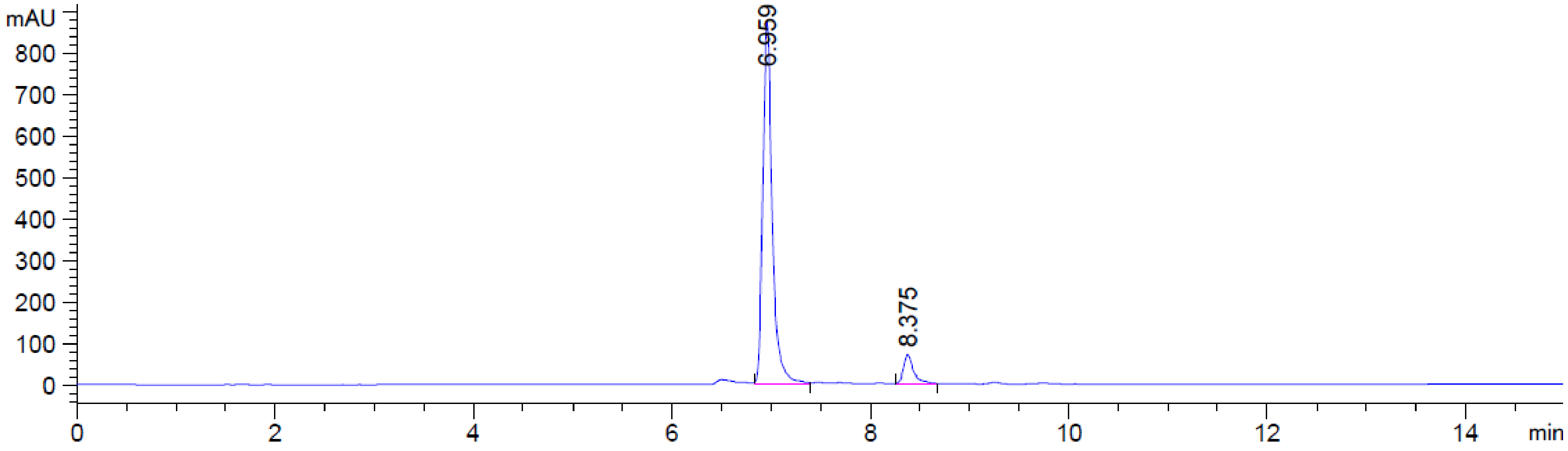

MS (-) ESI

(Calc. [M-H]$^-$ $C_{34}H_{46}N_{17}O_{25}P_4^-$ 1216.18067)

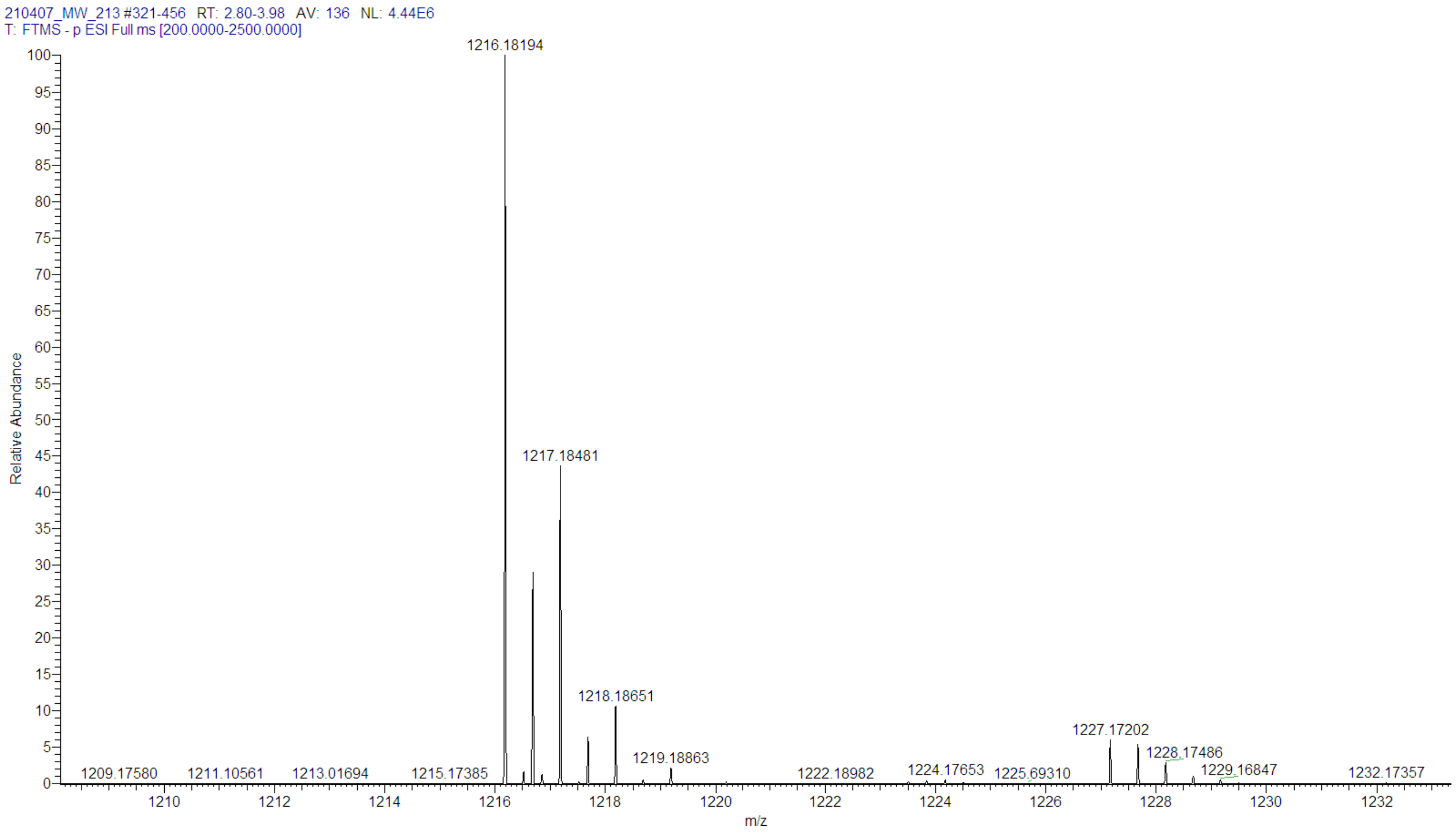

5b: NVA-L2N-GpppA$_m$pG

Chemical structure

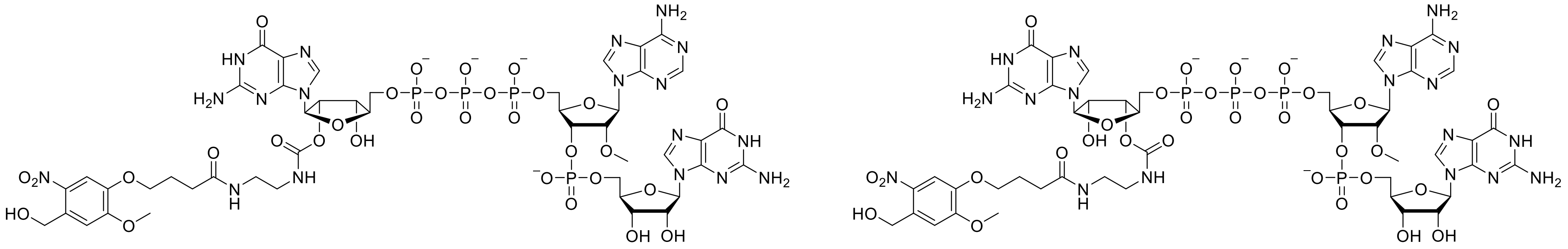

RP HPLC

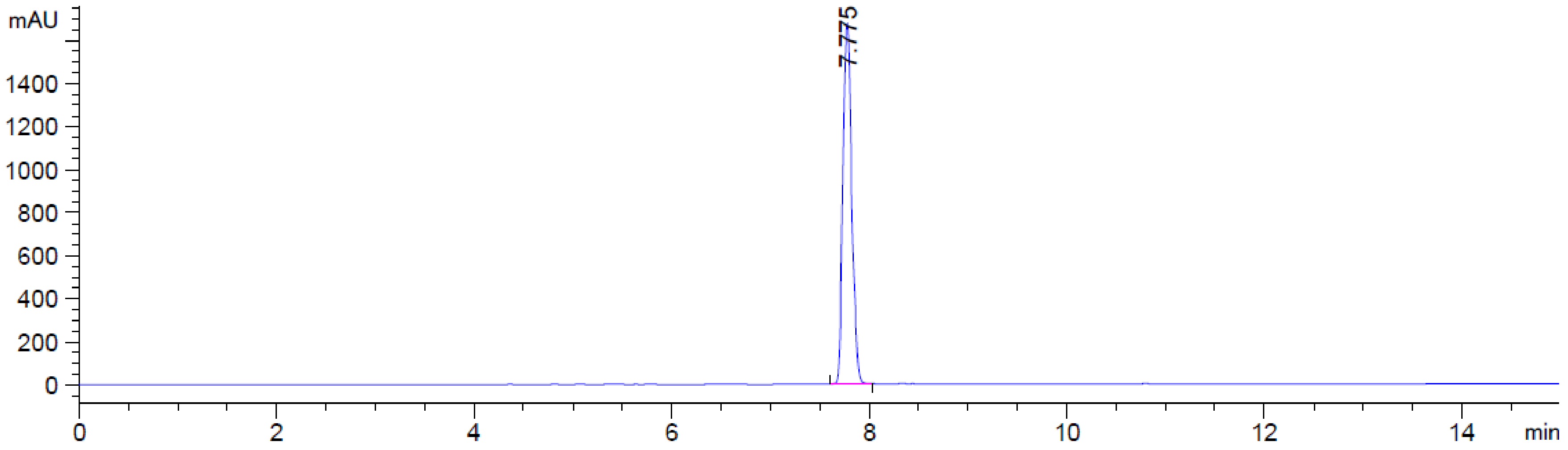

MS (-) ESI

(Calc. [M-H]$^-$ $C_{46}H_{59}N_{18}O_{31}P_4^-$ 1483.25496)

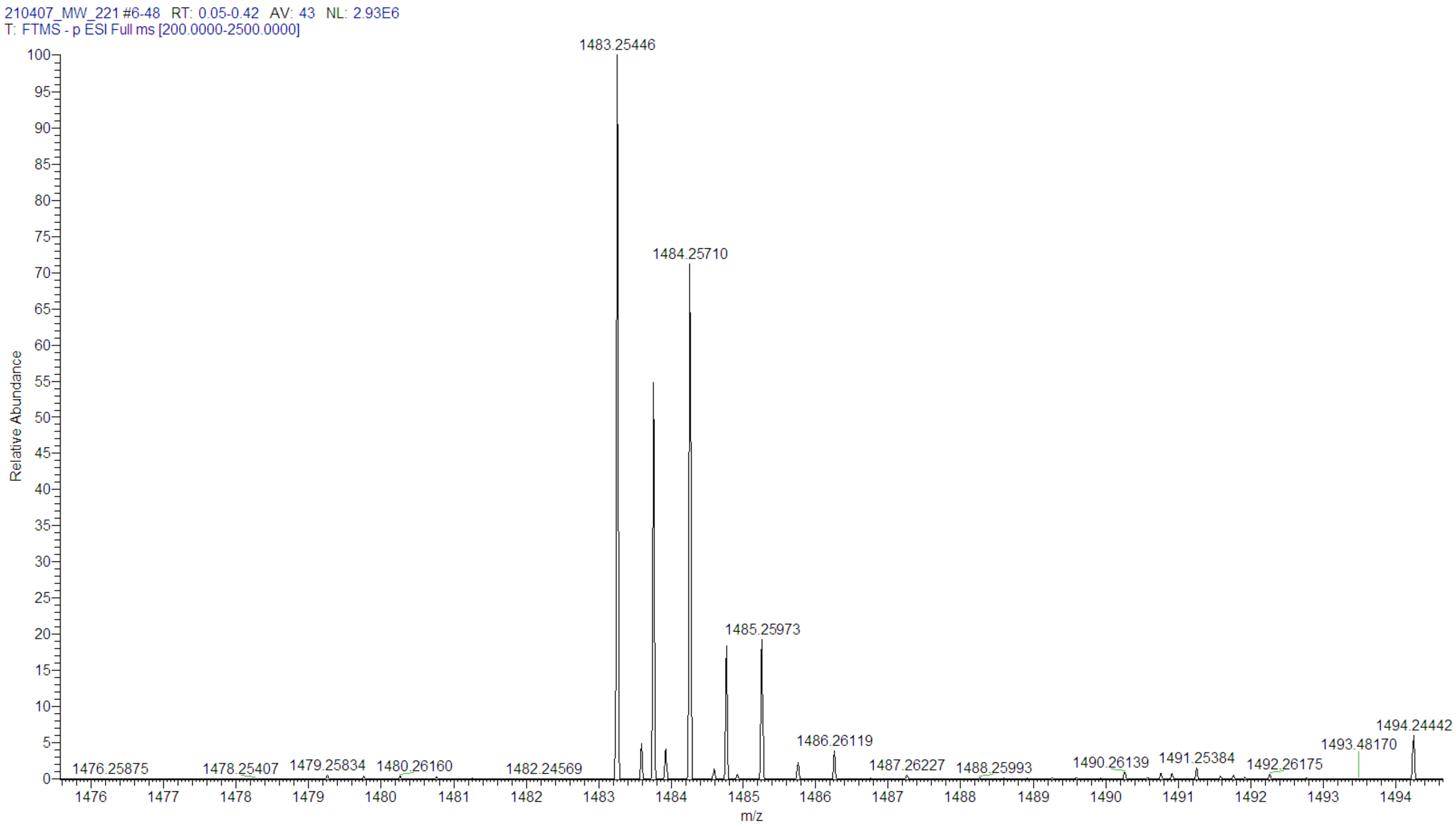

PAL-1a: Diaz-L2$_N$-GpppA$_m$pG-EDA

Chemical structure

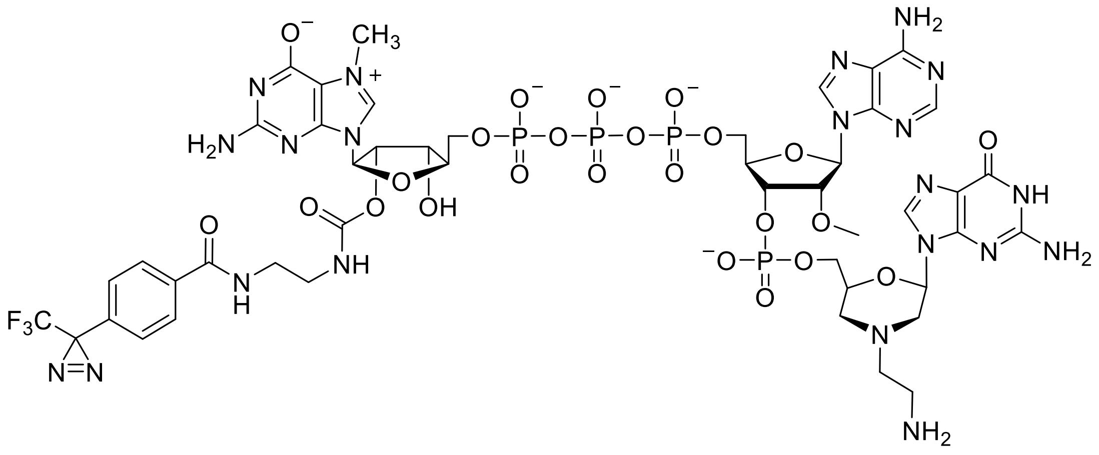

RP HPLC

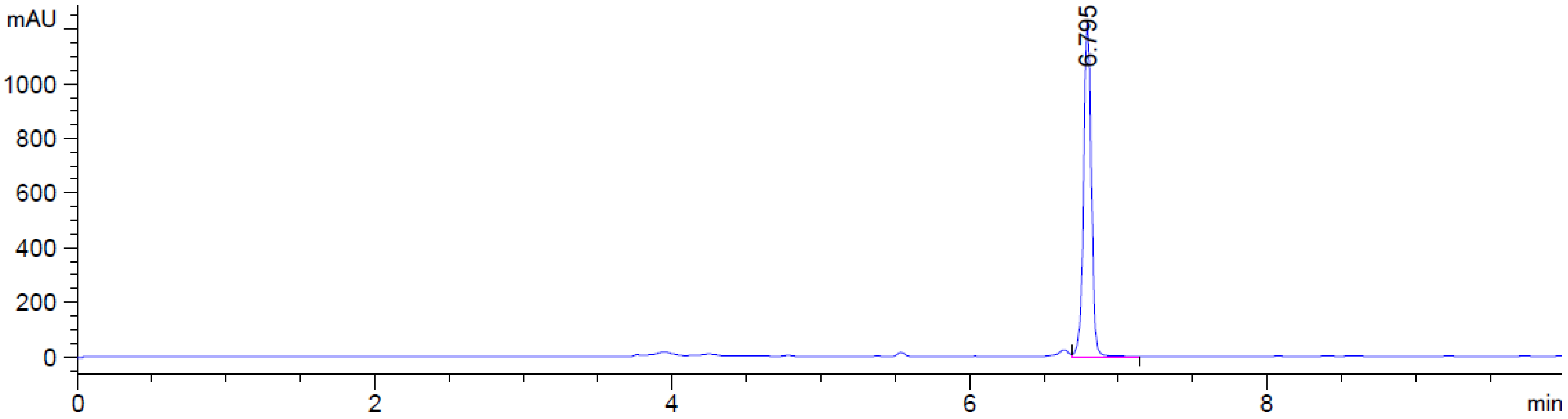

MS (-) ESI

(Calc. $[M-H]^-$ $C_{46}H_{57}F_3N_{21}O_{24}P_4^-$ 1468.27934)

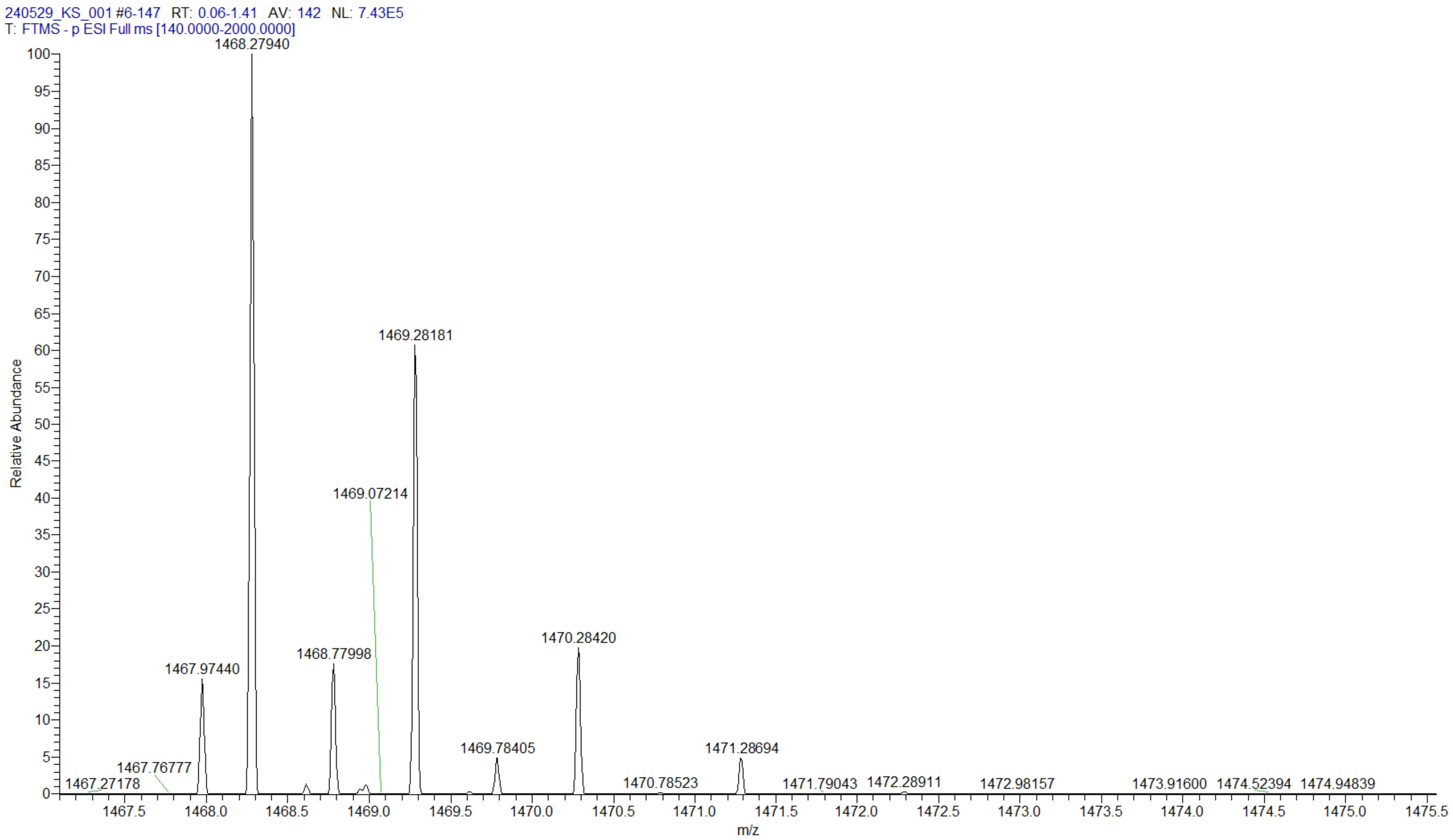

MS (+) ESI

(Calc. $[M+H]^+$ $C_{46}H_{59}F_3N_{21}O_{24}P_4^+$ 1470.29389)

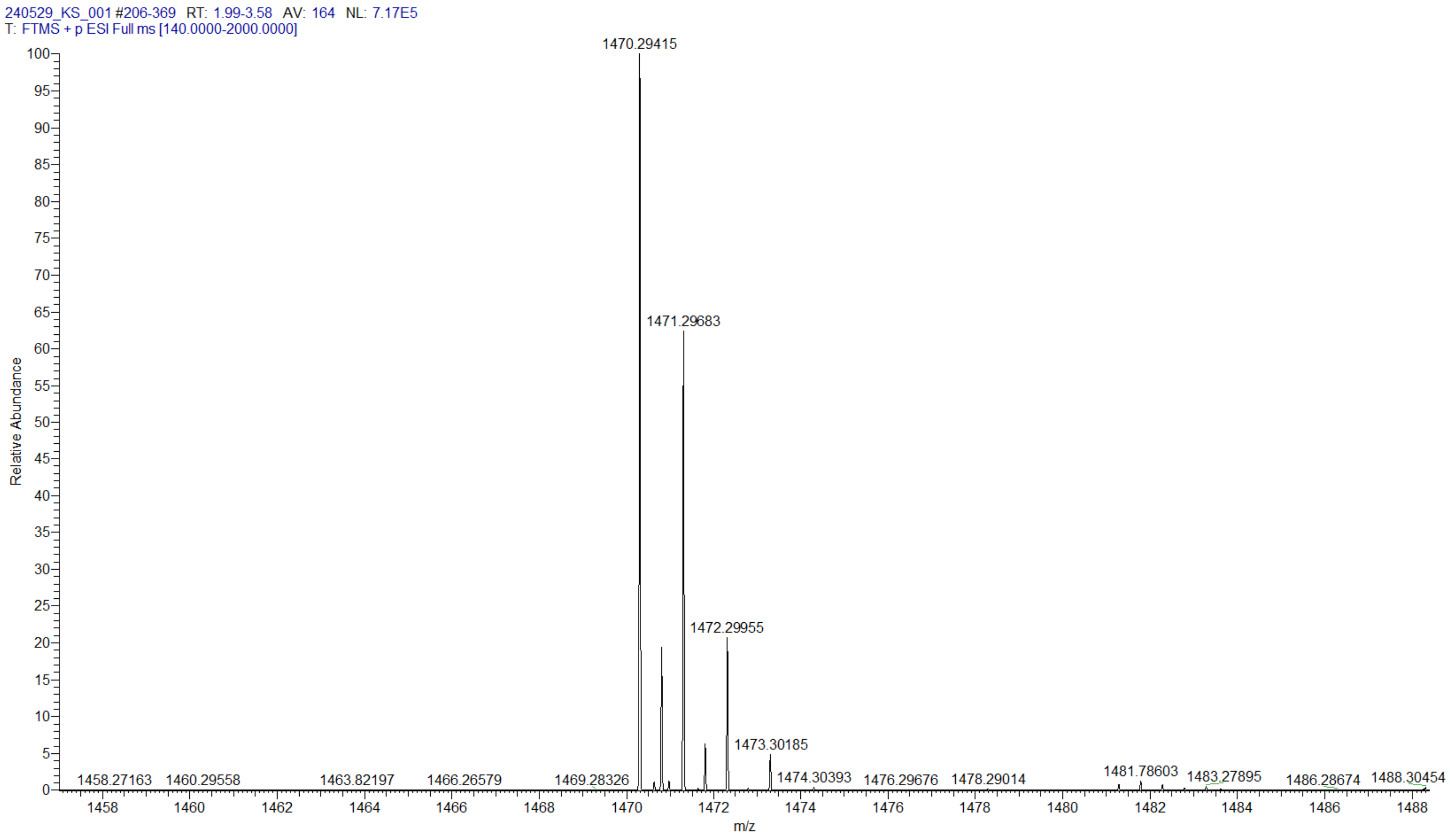

6: $p^{L3N}A_{m}pG$

Chemical structure

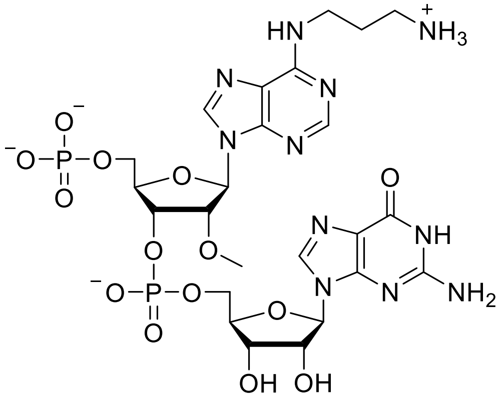

RP HPLC

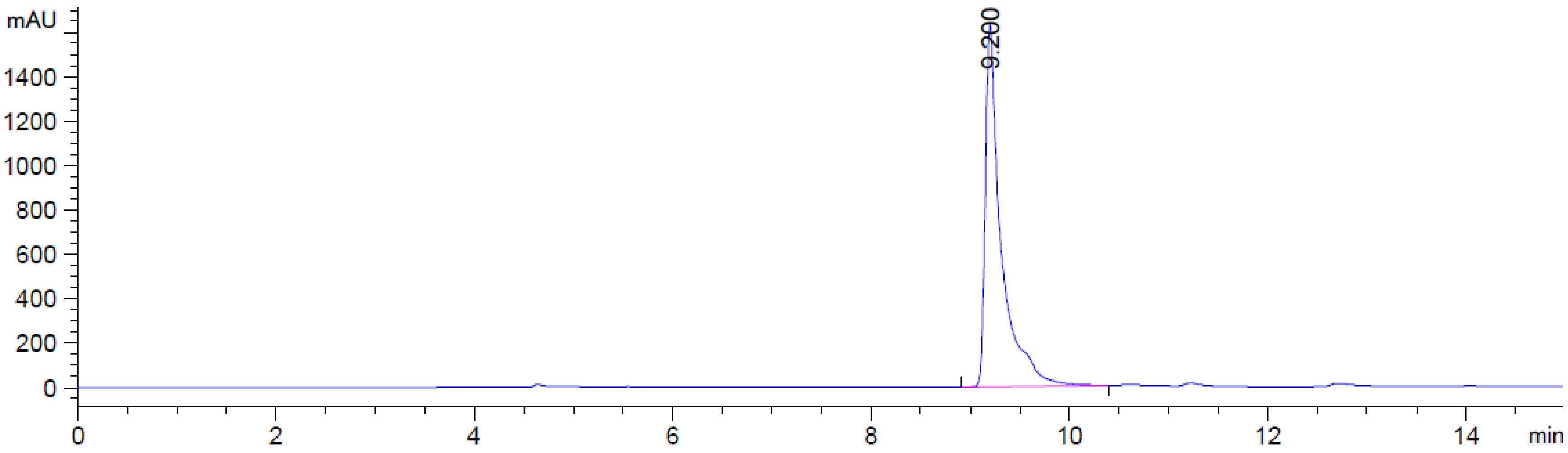

MS (-) ESI

(Calc. $[M-H]^-$ $C_{24}H_{34}N_{11}O_{14}P_2^-$ 762.17674)

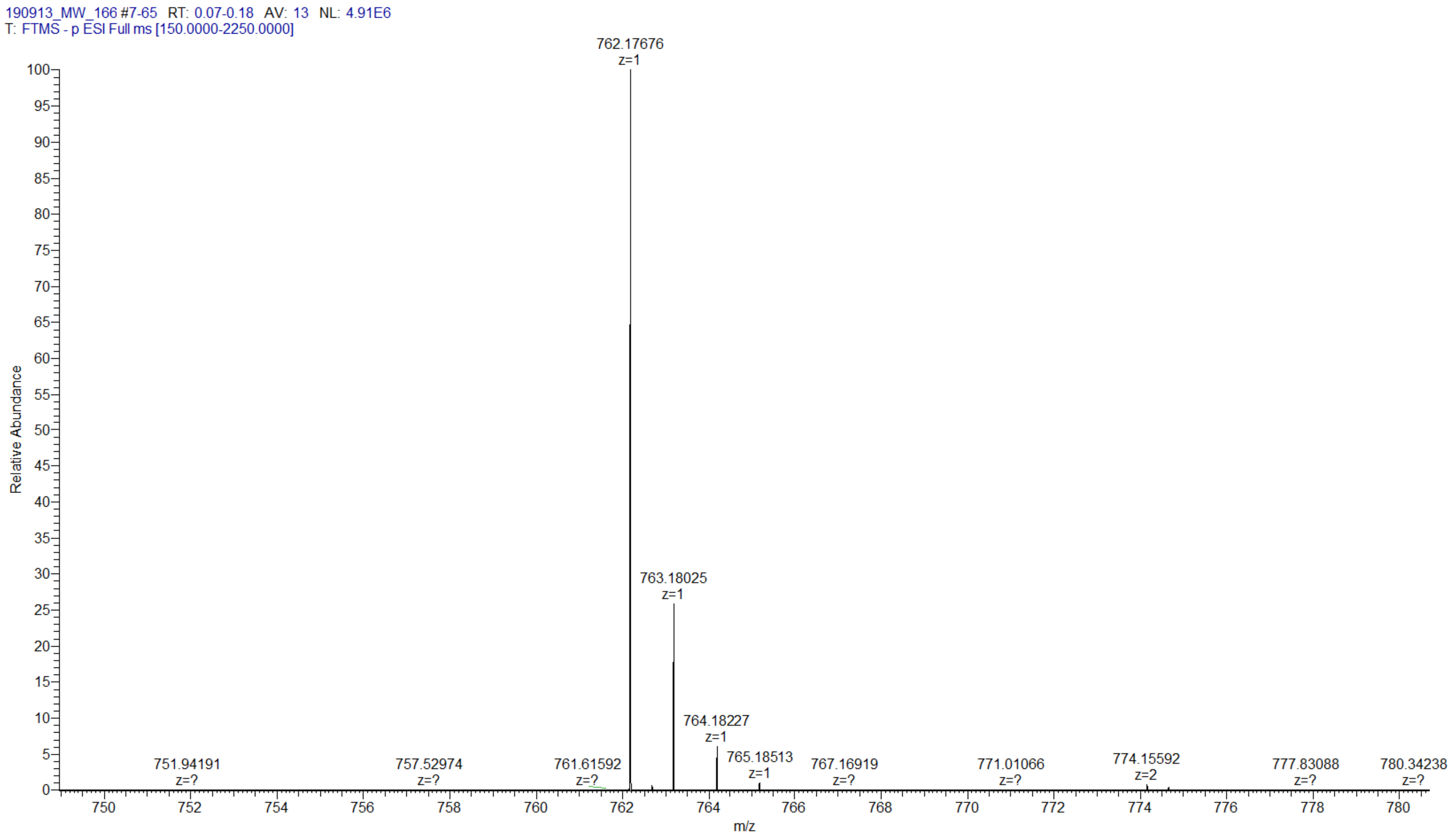

7: pA$_{L3N}$pG	
**Chemical structure**	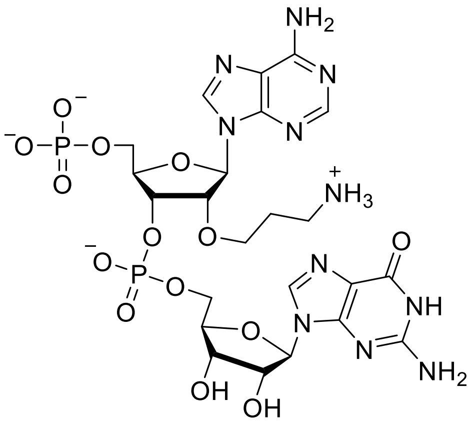
**RP HPLC**	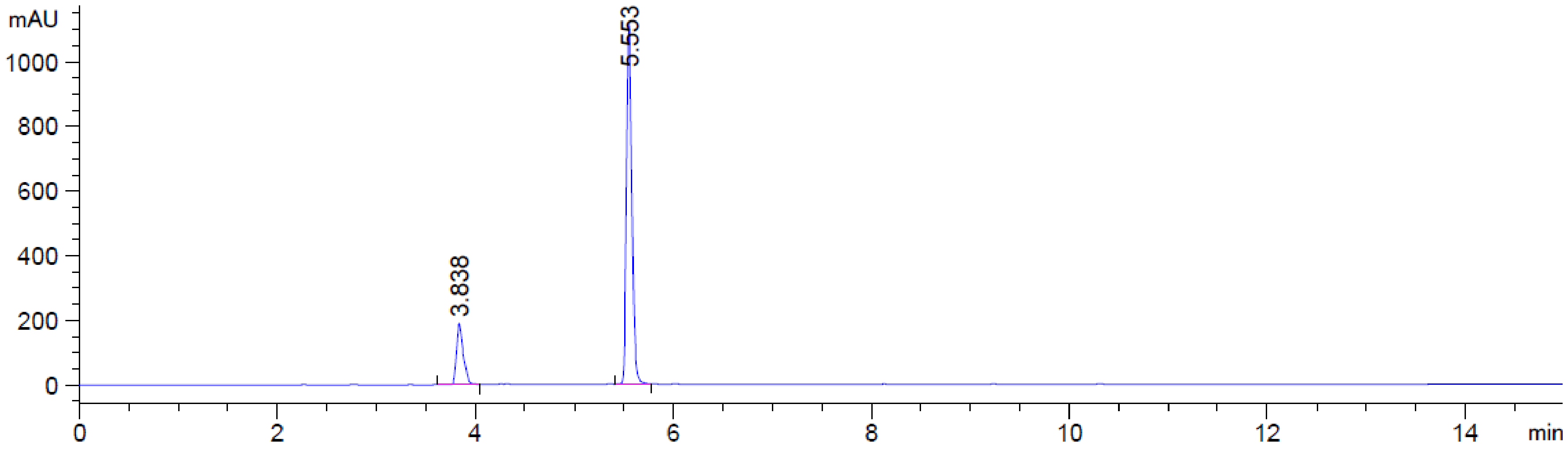

MS (-) ESI

(Calc. [M-H]$^-$ $C_{23}H_{32}N_{11}O_{14}P_2^-$ 748.16109)

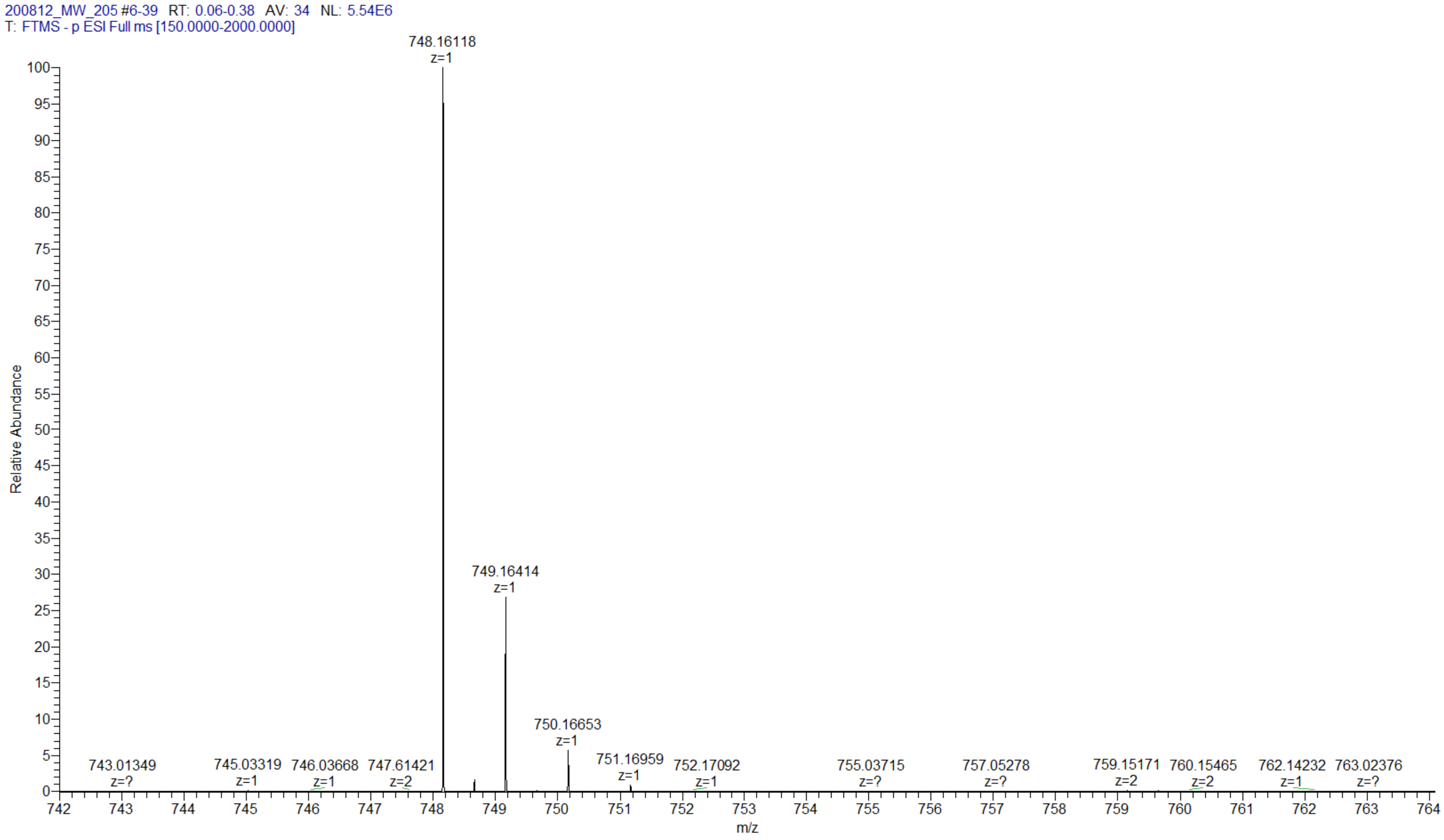

8: 3-methoxy-4-[3-(ethoxycarbonyl)propyloxy]benzalhedyde

^{1}**H NMR** (500 MHz, d-DMSO, 25°C)

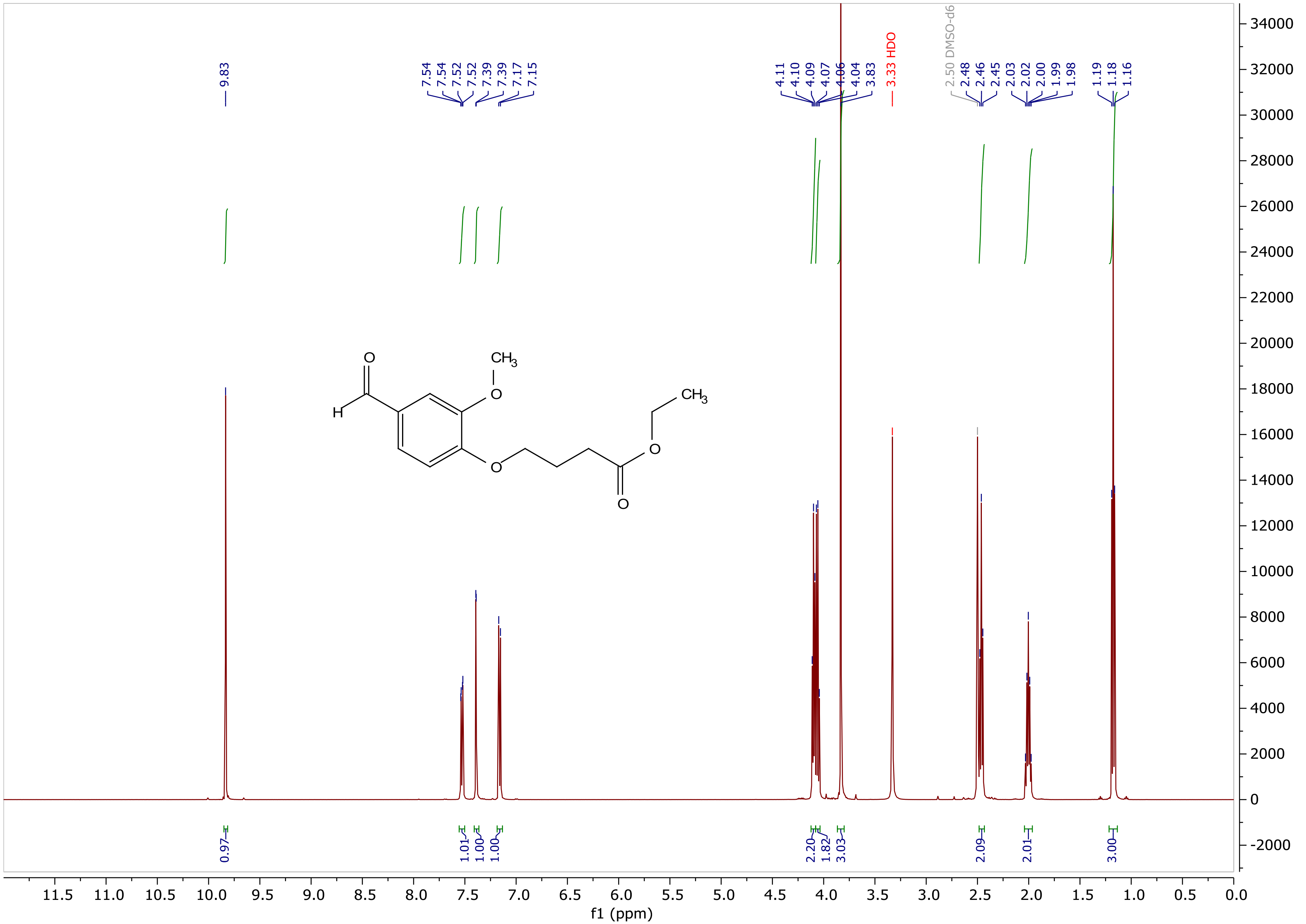

^{13}C NMR (126 MHz, d-DMSO, 25°C)

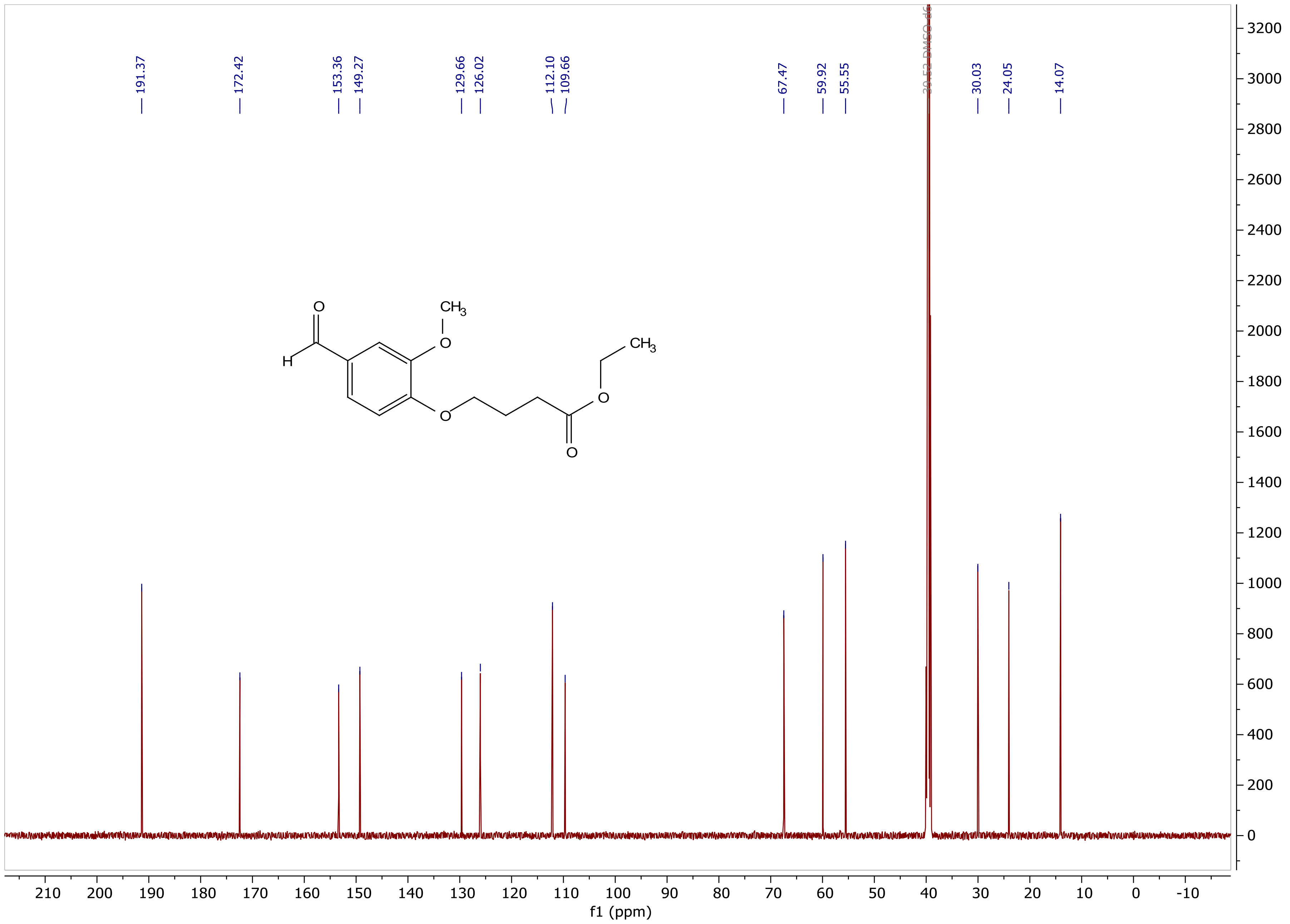

9: 3-methoxy-4-[3-(ethoxycarbonyl)propyloxy]-6-nitrobenzalhedyde

^{1}H NMR (500 MHz, d-DMSO, 25°C)

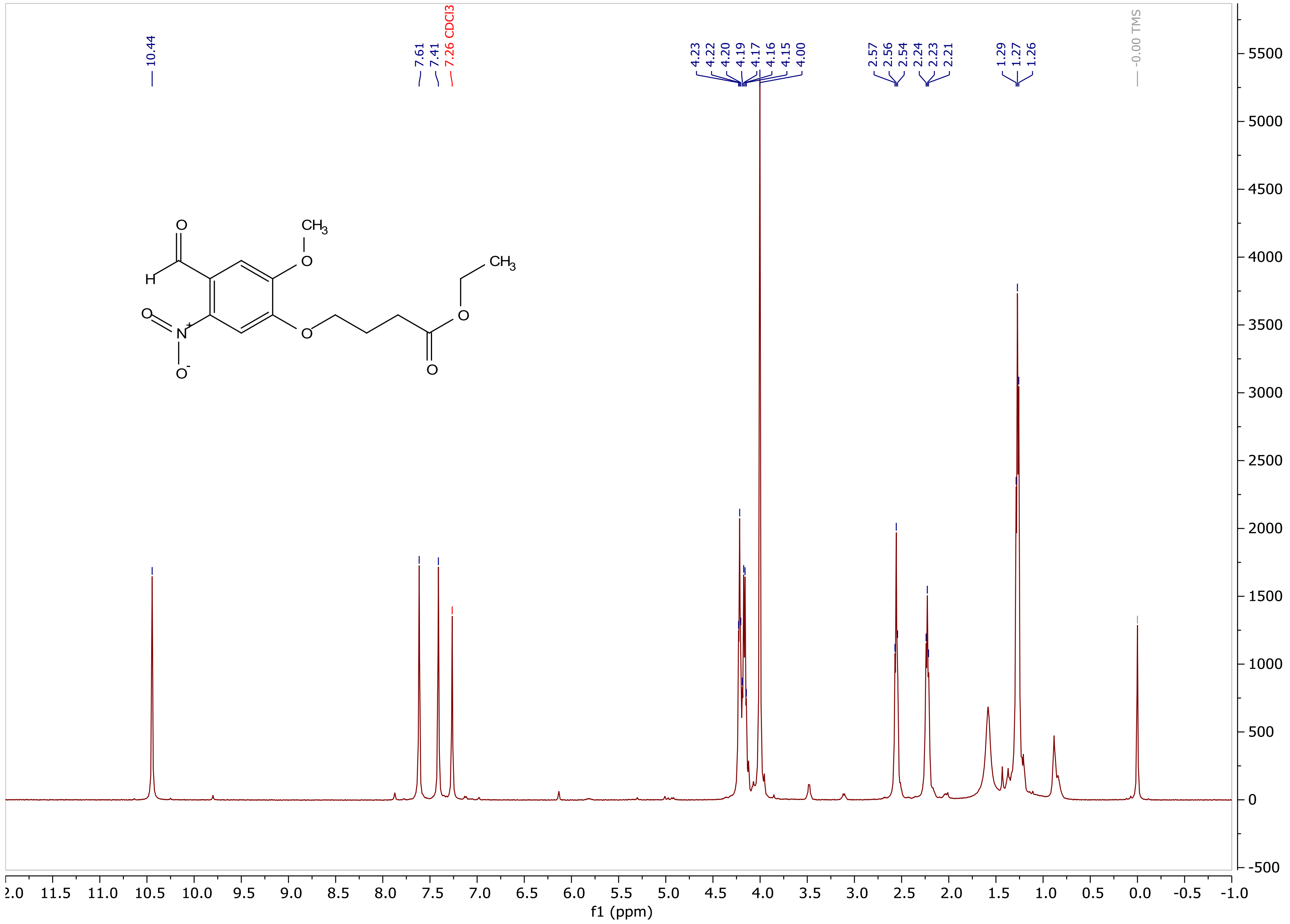

^{13}C NMR (126 MHz, d-DMSO, 25°C)

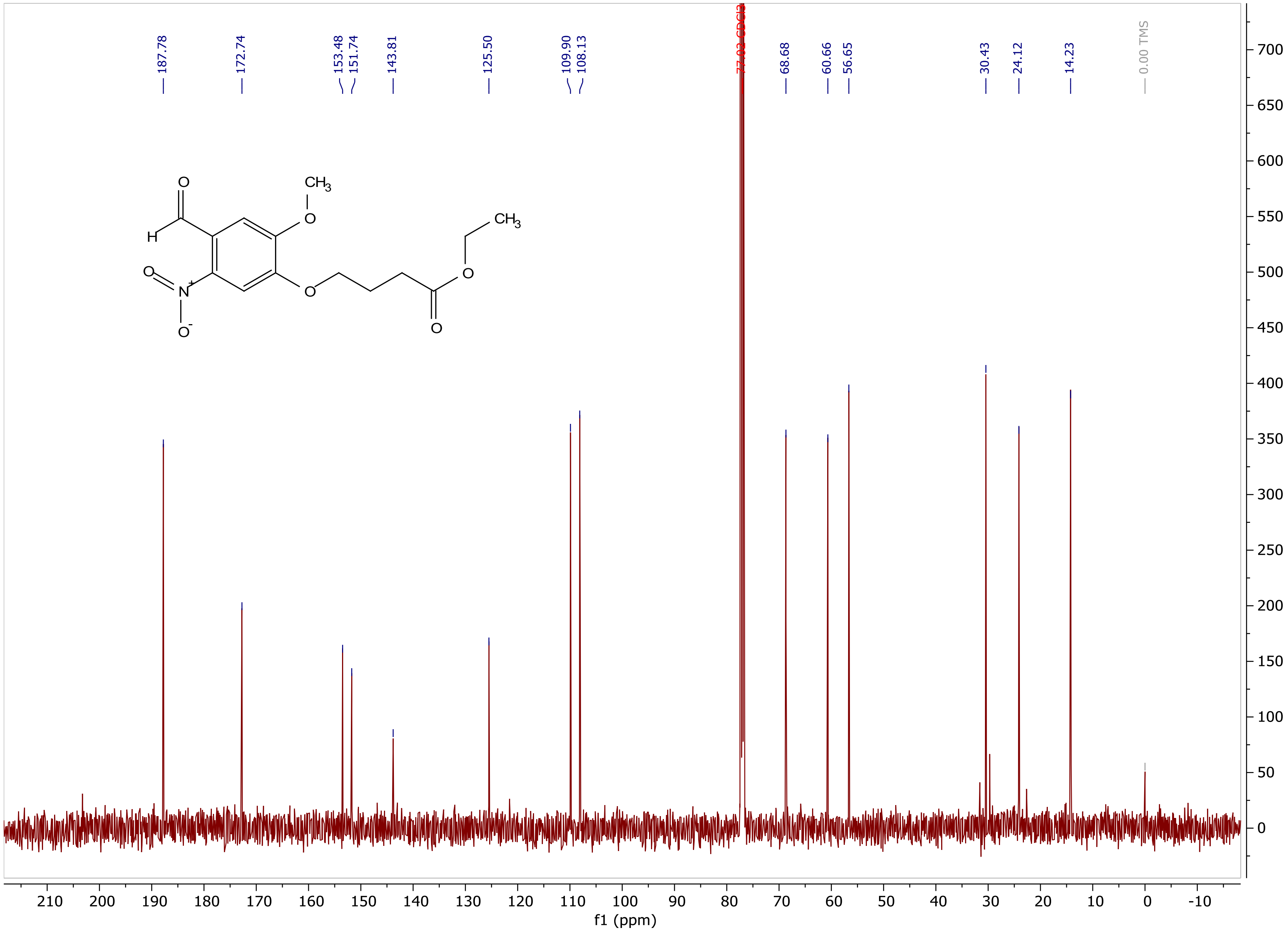

**10: 3-methoxy-4-(3-carboxypropyloxy)-6-nitrobenzalhedyde**	
**^{1}H NMR** (500 MHz, d-DMSO, 25°C)	

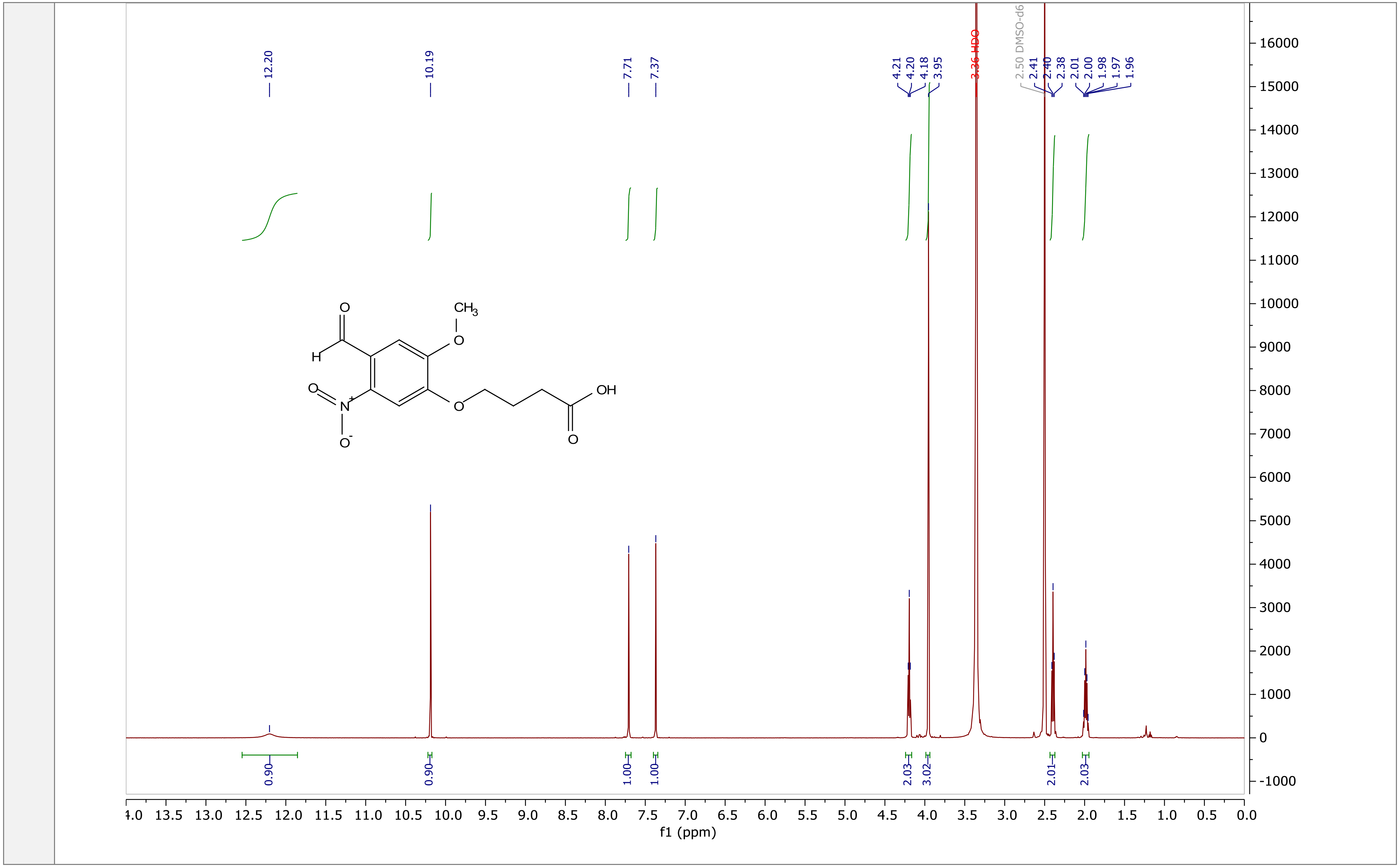
12.20
10.19
7.71
7.37
4.21
4.20
4.18
3.95
3.36 HDO
2.50 DMSO-d6
2.41
2.40
2.38
2.01
2.00
1.98
1.97
1.96
0.90
0.90
1.00
1.00
2.03
3.02
2.01
2.03
f1 (ppm)
O
H
CH3
O
OH
O
N+
O-
O

^{13}C NMR (126 MHz, d-DMSO, 25°C)

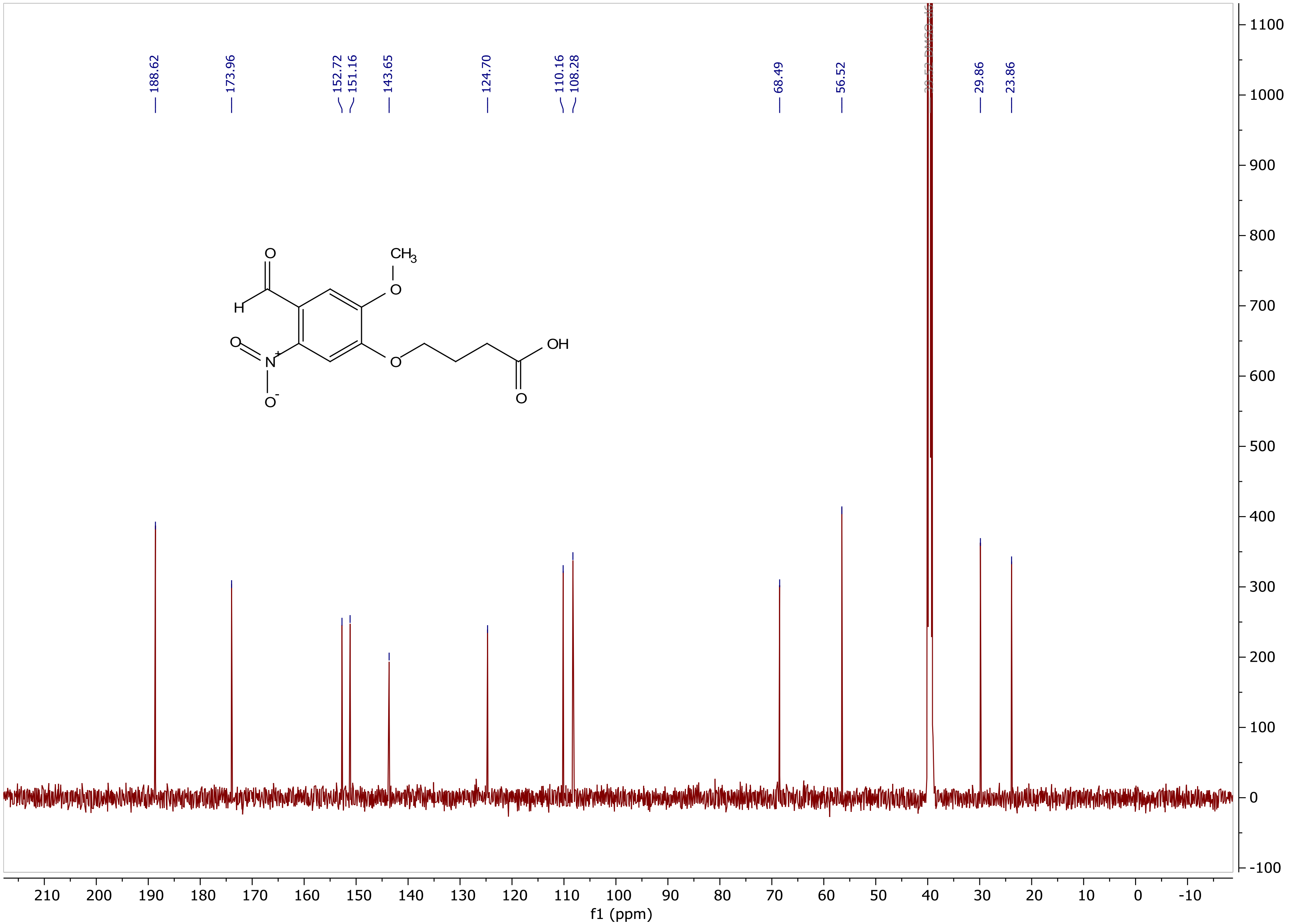

11: 3-methoxy-4-(3-carboxypropyloxy)-6-nitrobenzyl alcohol

^{1}H NMR (500 MHz, d-DMSO, 25°C)

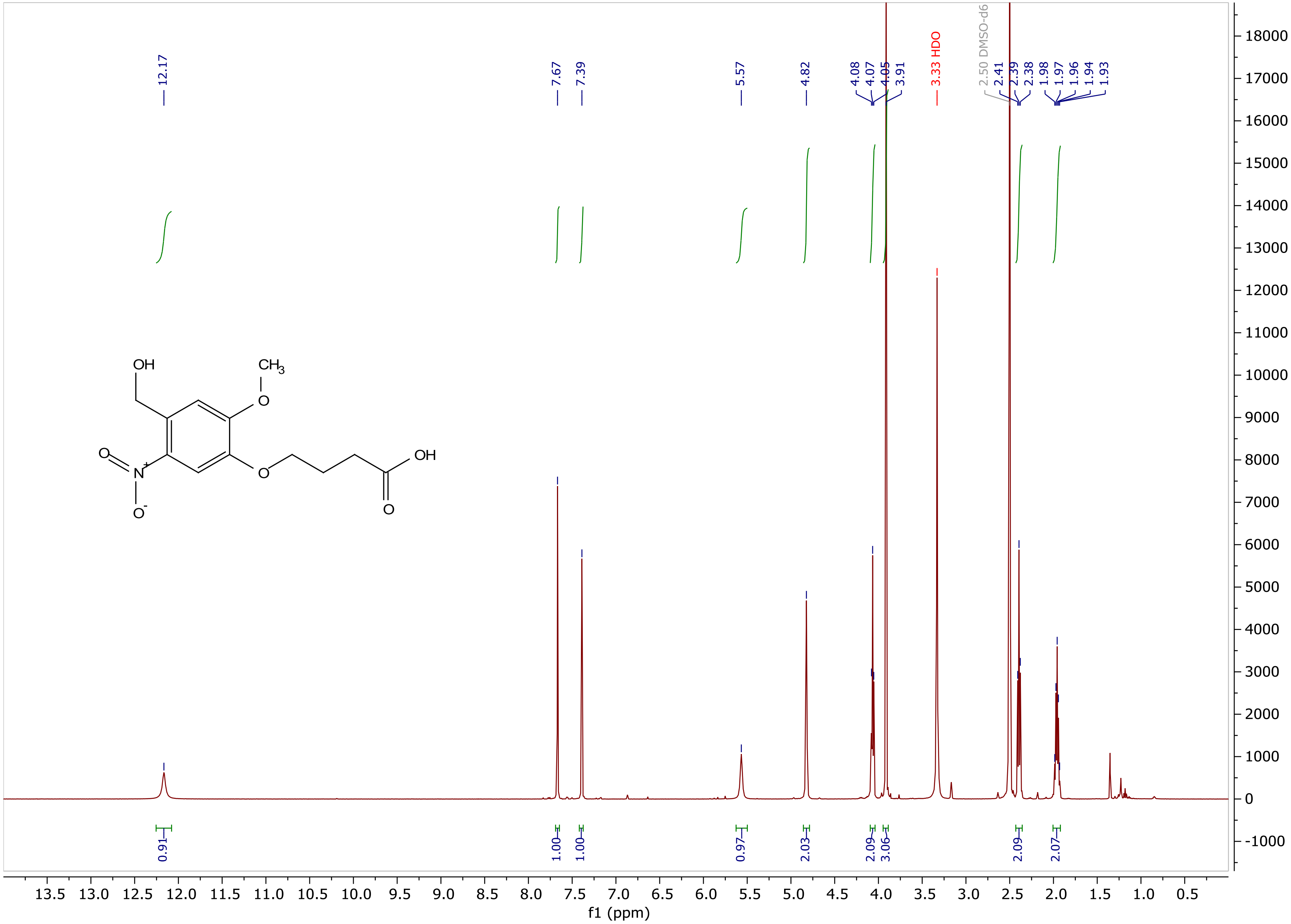

^{13}C NMR (126 MHz, d-DMSO, 25°C)

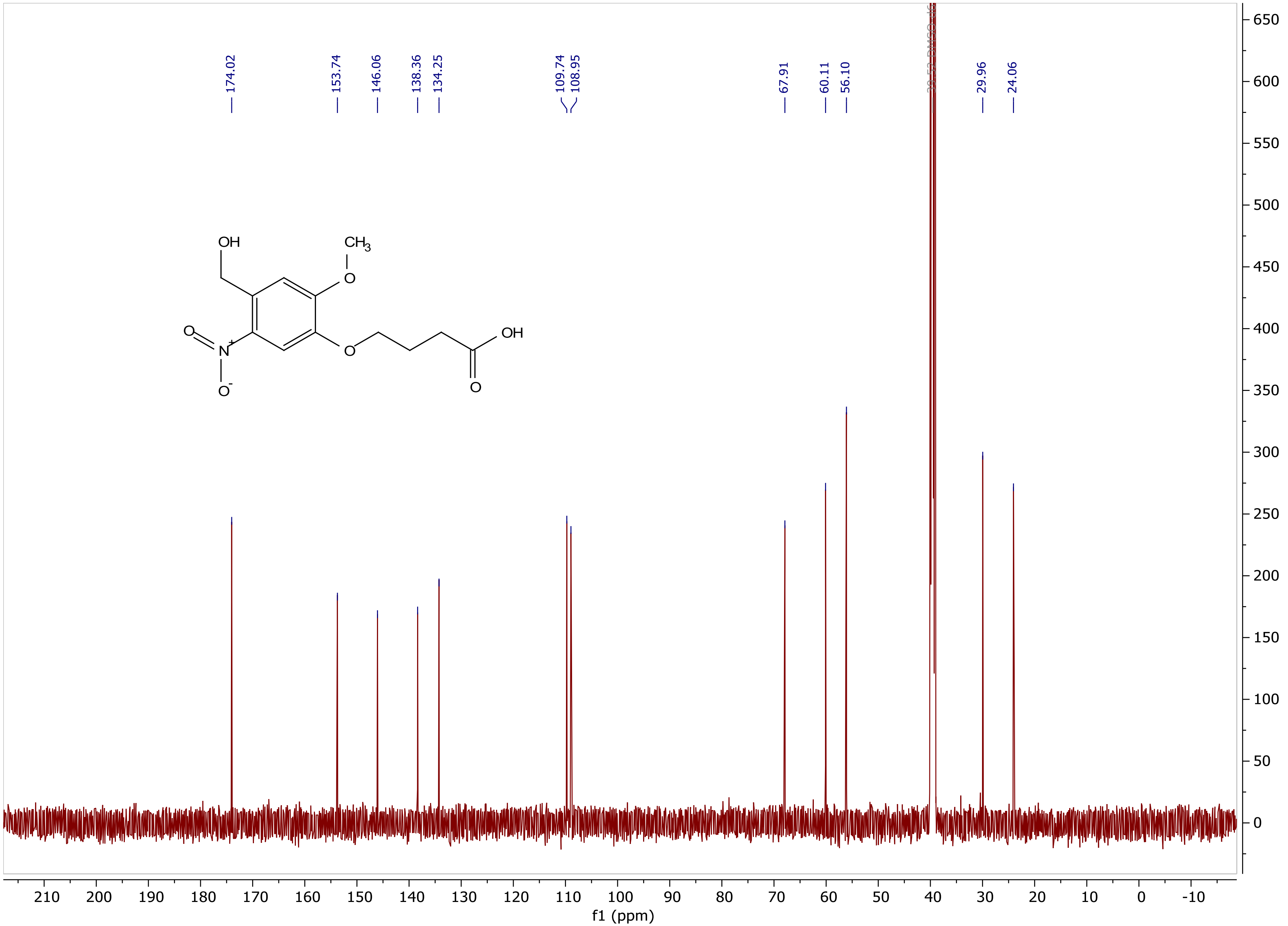

12: 3-methoxy-4-(3-succinimidylcarboxypropyloxy)-6-nitrobenzyl alcohol

^{1}H NMR (500 MHz, d-DMSO, 25°C)

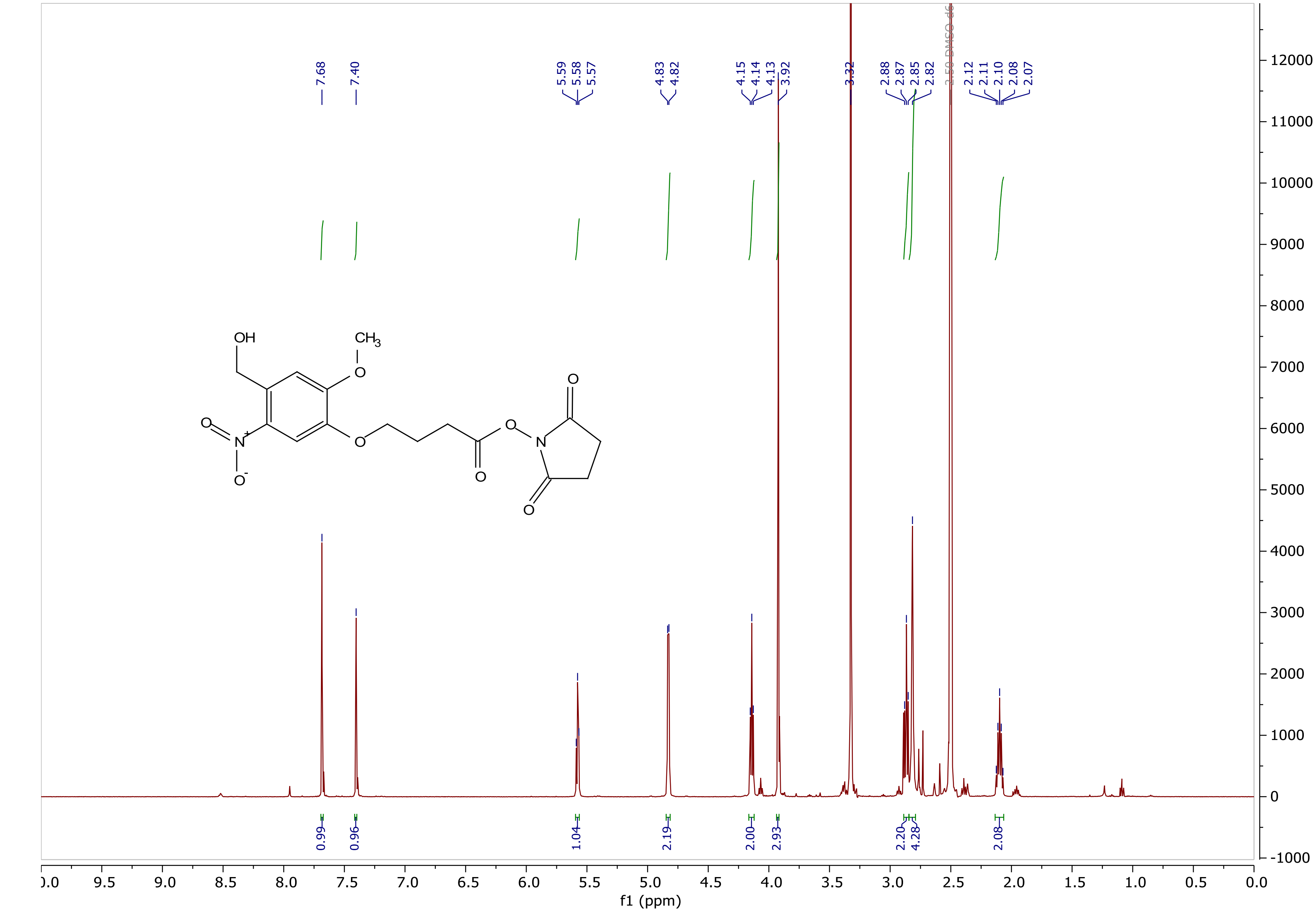

COSY NMR (d-DMSO, 25°C)

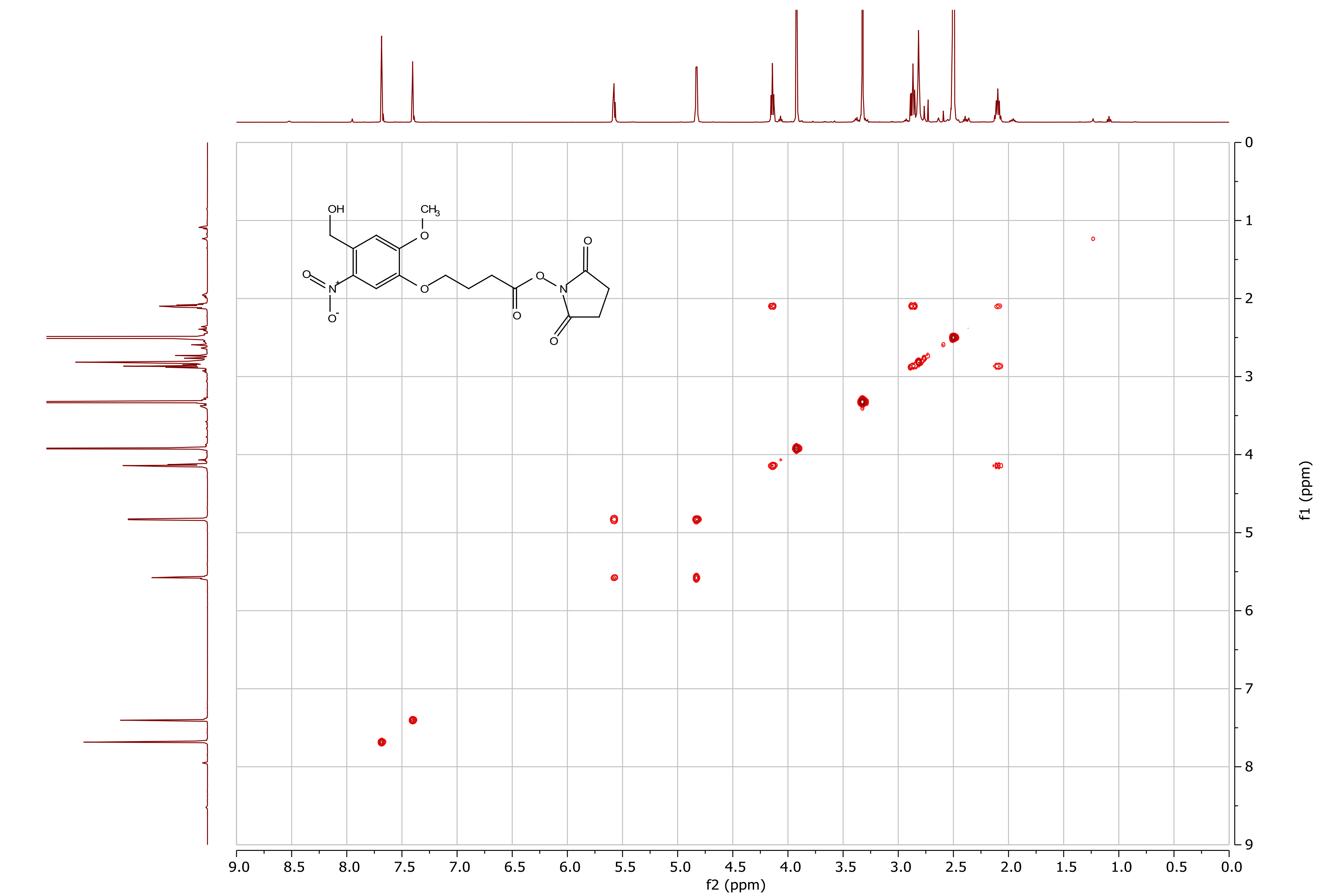

^{13}C HSQC MNR (d-DMSO, 25°C)

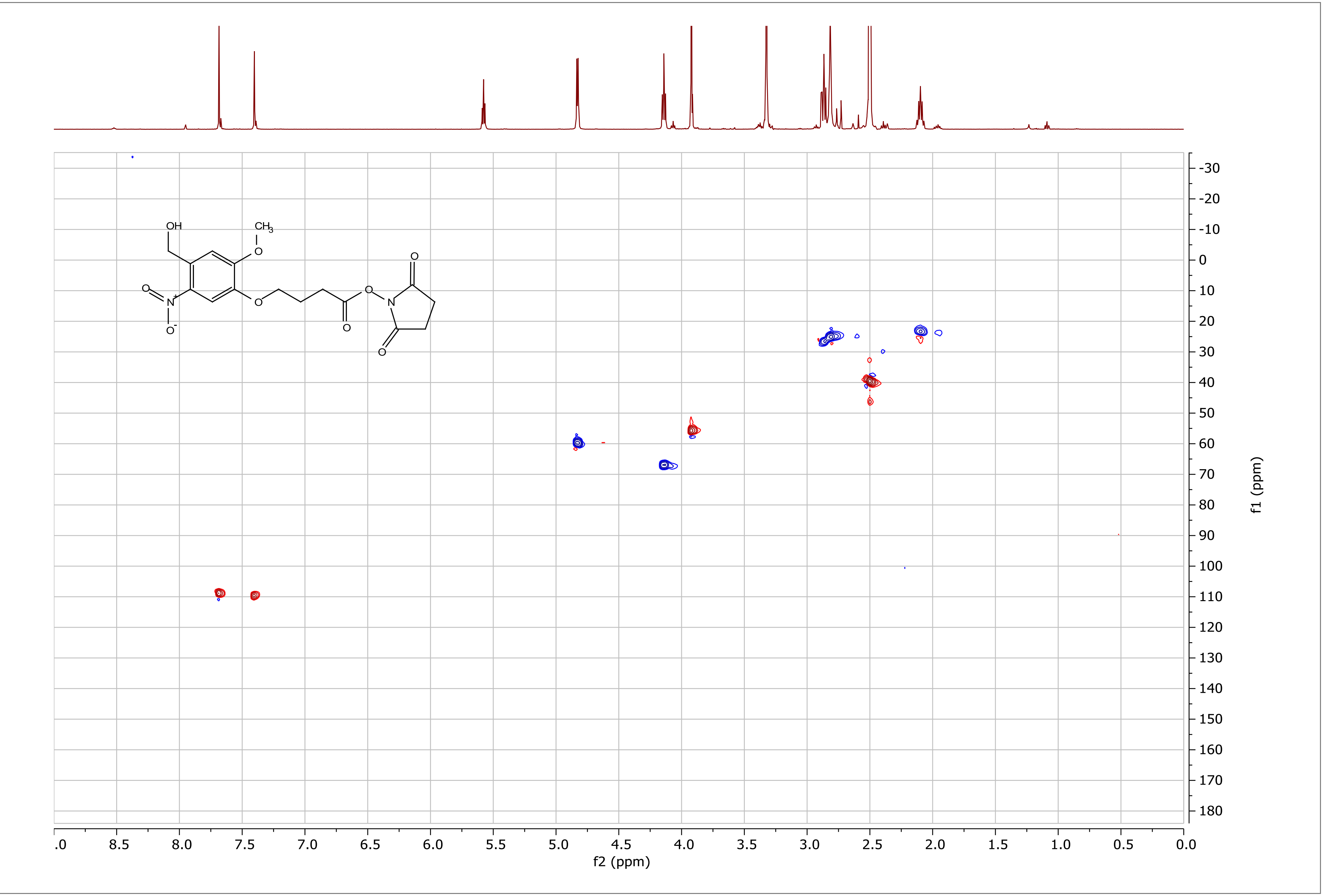

Supplement: Supplementary file 1 — Supporting Information [file ADVS-11-2400994-s001.pdf]
